# Druggable genome CRISPRi screen in hydrogels reveals regulators of cortactin-driven actin remodeling promoting glioblastoma invasion

**DOI:** 10.1172/jci.insight.196484

**Published:** 2026-08-10

**Authors:** Mufeng Hu, Anna Weldy, Isabella M. Lovalvo, Erin A. Akins, Saket Jain, Alexander Chang, Ankita Sati, Meeki Lad, Austin Lui, Akhil Rajidi, Ameya Kothekar, Erika A. Ding, Juan A. Oses Prieto, Pablo Estevez, Alma L. Burlingame, Sanjay Kumar, Manish K. Aghi

**Affiliations:** 1 UCSF Neurosurgery, UCSF, San Francisco, California, USA.; 2 UC Berkeley Chemical and Biomolecular Engineering,; 3 UC Berkeley Bioengineering, and; 4 UC Berkeley-UCSF Graduate Program in Bioengineering, University of California Berkeley, Berkeley, California, USA.; 5 UCSF Department of Pharmaceutical Chemistry, UCSF, San Francisco, California, USA.; 6 California Institute for Quantitative Biosciences at UC Bereley (QB3-Berkeley), Berkeley, California, USA.; 7 UCSF Bioengineering and Therapeutic Sciences, UCSF, San Francisco, California, USA.

**Keywords:** Cell biology, Oncology, Brain cancer, Cell migration/adhesion, Drug screens

## Abstract

To identify therapeutic targets limiting glioblastoma invasion, we applied druggable genome CRISPRi screens and multiomic analysis to patient-derived glioblastoma cells in micro-dissectible biomimetic 3D hydrogels that permitted separation and analysis of core versus invasive fractions. Of 2,550 genes screened, 12 encoded druggable targets whose suppression limited invasion, of which *AURKB* (encoding aurora kinase B) and *ACP1* (encoding low molecular weight protein tyrosine phosphatase, LMW-PTP) were validated in neurosphere assays and in vivo. Proximity labeling identified cortactin as a link between LMW-PTP and aurora B, and we observed that cortactin underwent serine phosphorylation by aurora B and tyrosine dephosphorylation by LMW-PTP. Targeting *ACP1* or *AURKB* via CRISPRi or inhibitors in culture and in vivo shifted the cortactin phosphorylation balance in glioblastoma, reducing levels of cortactin and the actin-related protein 2/3 (Arp2/3) complex that mediates cortactin-induced actin stabilization, thereby reducing actin-cortactin-Arp2/3 colocalization and subsequent actin polymerization. *AURKB* or *ACP1* targeting shifted actin from cytoplasm to the nucleus, reducing mesenchymal gene expression. Biophysical analysis implicated *AURKB* in glioblastoma cell adhesion and stiffness needed for initial migration and *ACP1* in mechanical stress resistance required for later migration. These findings revealed a targetable axis balancing kinase and phosphatase activities to regulate actin polymerization during glioblastoma invasion.

## Abstract 2

The poor prognosis of glioblastoma, a malignant brain cancer, arises from its invasiveness. We performed a druggable genome screen in glioblastoma cells invading brain-mimetic hydrogels, revealing Aurora B and LMW-PTP as invasion mediators.

## Graphical abstract

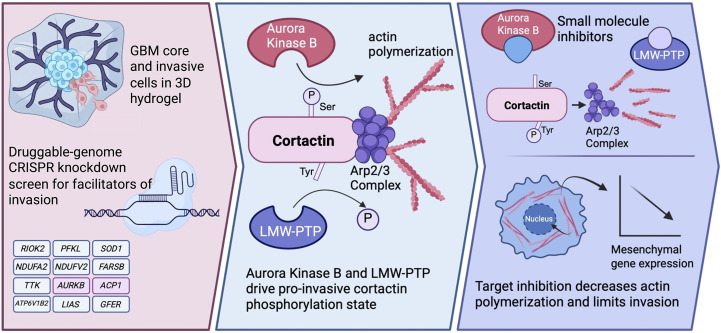

## Introduction

Glioblastoma (GBM), the most common primary malignant brain tumor (1), is characterized by robust invasive capacity (2). Standard treatment — maximal surgical resection followed by radiation and temozolomide chemotherapy (3) — fails to control the disease; median survival is 15 months from diagnosis (1). First, treatment modalities are impeded by tumor invasiveness, which precludes complete surgical resection. Second, invasive cells migrate beyond the radiation field, typically 2 cm beyond the enhancing tumor edge (4). Third, invasive GBM cells escape the MRI enhancement area, where the blood-brain barrier (BBB) is disrupted, and enter regions where the BBB remains intact, shielding cells from systemic chemotherapy. These factors facilitate the inevitable recurrence defining GBM (2).

Although targeting invasion has been proposed as a strategy for treating GBM, translational progress has been slowed by two limitations of most studies. First, studies have failed to recognize that infiltrating GBM cells extending beyond the tumor edge evolve unique adaptive cellular machinery from local stressors encountered during invasion. These include mechanical constraints of the brain extracellular matrix (ECM), interstitial fluid pressure in regions of peritumoral edema, and a shift from hypoxia in the tumor core toward intense metabolic activity driving higher levels of ROS toward the tumor edge (5, 6). Unfortunately, cells at the invasive tumor front are rarely those sampled in studies analyzing banked tumor tissue, typically from the more accessible tumor core. Second, most invasion studies have used 2D culture systems coated with ECM proteins, failing to capture the dimensionality, biomechanics, and heterogeneity of GBM invasion, as cells adherent to 2D surfaces experience a different mechanical microenvironment than cells fully encapsulated in a 3D biomaterial.

Consequently, we developed micro-dissectible biomimetic 3D hyaluronic acid–RGD hydrogels containing hyaluronic acid plus an RGD peptide sequence (Arg-Gly-Asp integrin-binding motif) enhancing cell adhesion and migration by interacting with cellular integrin receptors, replicating a key feature of the GBM microenvironment (5). We previously leveraged the micro-dissectible nature of these devices to separate GBM cells in the implanted core from those invading the hydrogels and performed metabolomics and transcriptomics on both sets of cells to validate these devices against site-directed biopsies from patient GBMs (5).

**Figure 1:**
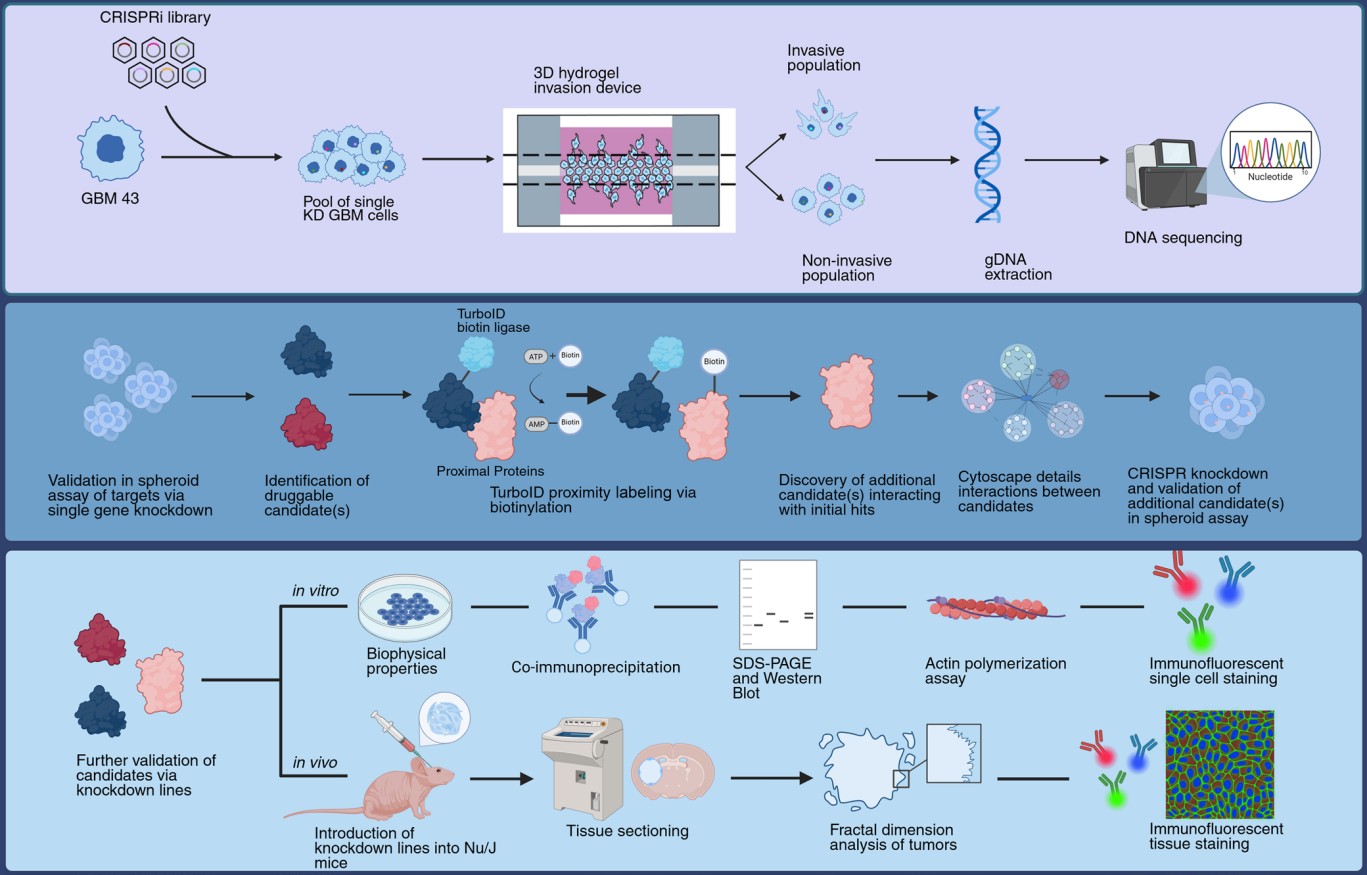
Overview of the multiomic workflow used to identify targetable mediators of invasion in GBM. Shown are the steps of the study’s discovery, proteomic, and functional assessment phases. During discovery, GBM43 cells expressing a CRISPRi library targeting the druggable human genome invaded 3D hyaluronic acid–RGD devices; after device disassembly, invasive versus core cells were sequenced to identify sgRNAs enriched in core cells. During proteomics, candidate sgRNAs were validated via spheroid assays; proximity labeling assays identified binding partners of the proteins encoded by the genes targeted by these sgRNAs, which can be targeted in follow-up studies. During functional assessment, studies were performed in vitro, in culture, and in vivo on candidates from earlier phases. Created in BioRender.

Here, we employed a broader multiomics approach involving a CRISPRi screen targeting the druggable human genome to identify targetable mediators of GBM invasion, followed by proteomics to identify binding partners of their encoded proteins (Figure 1). The druggable genome refers to 2,550 of nearly 30,000 genes in the human genome encoding a protein with precedent for binding a drug-like molecule (7), and mostly falls into 7 categories: G protein–coupled receptors (*n* = 802), serine/threonine and tyrosine protein kinases (*n* = 519), serine proteases (*n* = 503), protein phosphatases (*n* = 156), zinc metallopeptidases (*n* = 106), nuclear hormone receptors (*n* = 75), and phosphodiesterases (*n* = 24). Although less than half of these druggable genes are potential drug targets based on roles in human disease, and only 120 have been targeted by marketed drugs (8), druggable genome screens are additionally beneficial in identifying mechanisms of biological processes.

During this screen, we identified the tyrosine phosphatase acid phosphatase 1 (ACP1) encoding low molecular weight protein tyrosine phosphatase (LMW-PTP), encoded by the *ACP1* gene, and the serine/threonine kinase aurora kinase B or aurora B, encoded by the *AURKB* gene, as druggable mediators of GBM invasion. Proximity labeling and proteomics revealed that these 2 proteins share a common target: cortactin, encoded by the *CTTN* gene, an actin-binding protein regulating lamellipodia formation. This approach bridges the gap between experimental models and clinical realities, advancing our understanding of GBM invasion and facilitating development of effective therapies targeting this process.

## Results

### CRISPRi screen of the druggable genome identifies mediators of GBM invasion.

**Figure 2:**
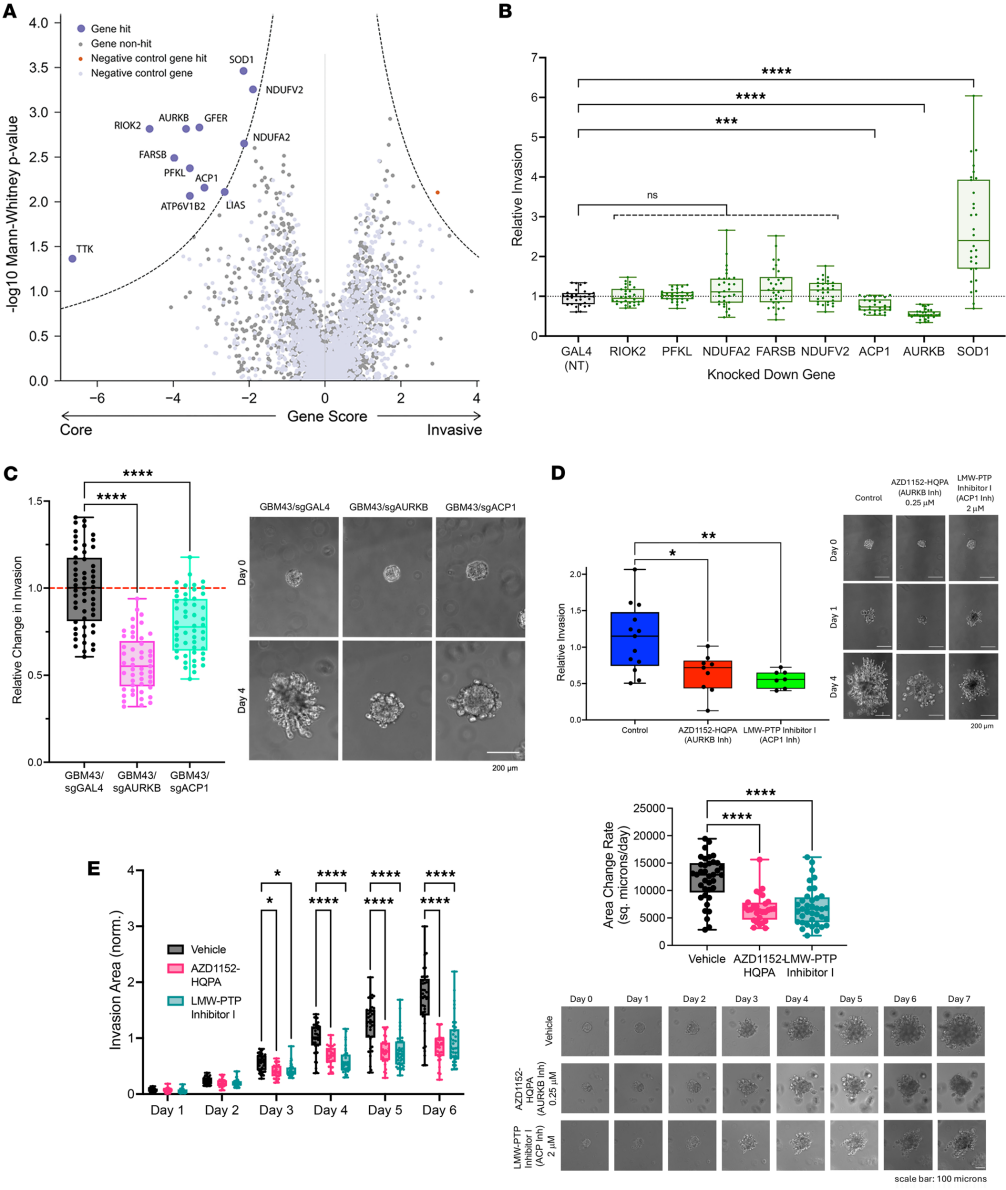
CRISPRi screen identifies druggable genes mediating GBM invasion in 3D hydrogels. (**A**) Volcano plot displaying statistical significance (*y* axis) versus gene score, quantifying the average of the top 3 enrichments among 5 sgRNAs targeting the gene in the invasive front versus core (*x* axis) of GBM43 cells expressing an sgRNA library targeting the druggable genome before invading 3D hydrogels. Dashed lines represent cutoff for hits (FDR = 0.01); genes left of the dashed lines had sgRNAs enriched in the invasive front versus core. (**B**) Quantification of spheroid invasion assays of 8 single-gene CRISPRi knockdown GBM43 cell lines from screen hits compared with control cells expressing dCas9 and sgRNA targeting GAL4 (NT, nontargeting control). Only targeting *AURKB* or *ACP1* slowed spheroid invasion (*P* < 0.0001; *n* = 30 spheres/group from 3 independent experiments). (**C**) Repeat quantification with larger sample size and representative images of GBM43 spheroid invasion assays with CRISPRi knockdown of *AURKB* and *ACP1*. (*P* < 0.0001; *n* = 49–56 spheres/group from 3 independent experiments). Original magnification, ×10; scale bar: 200 μm. (**D**) Quantification and representative images of spheroid invasion assays of GBM43 cells treated with aurora B inhibitor AZD1152-HQPA (barasertib) or LMW-PTP inhibitor I (MLS-0322825 Cmpd23); both slowed spheroid invasion (*P* = 0.01 AZD1152-HQPA, *P* = 0.006 LMW-PTP inhibitor; *n* = 7–13 spheres/group from 3 independent experiments). Original magnification, ×10; scale bar: 200 μm. (**E**) GBM43 spheroids treated with AZD1152-HQPA or LMW-PTP inhibitor I revealed a comparable timeline of slowed invasion beginning on day 3 (*P* < 0.05) and becoming robust from day 4 until day 6 (*P* < 0.0001); data assessment revealed that AZD1152-HQPA or LMW-PTP inhibitor I dramatically slowed invasive velocity over 6 days (*P* < 0.0001). *n* = 25–37 spheroids/group; ns, not significant; *P < 0.05; **P < 0.01; ***P < 0.001; ****P < 0.0001. For box and whisker plots, the horizontal line is the median; the box extends from 25th to 75th percentile and the whiskers from minimum to maximum value. One-way (**B**–**D** and right side of **E**) and 2-way (left side of **E**) ANOVA with Tukey’s pairwise post hoc comparisons.

To identify druggable mediators of GBM cell invasion in an unbiased manner, we performed a CRISPRi knockdown screen with a 13,250 sgRNA library targeting the 2,550 genes in the druggable human genome (Supplemental Table 1; supplemental material available online with this article; https://doi.org/10.1172/jci.insight.196484DS1). GBM43 cells (MGMT unmethylated/proneural subtype) (9) expressing dCas9/KRAB/MeCP2 and the sgRNA library were seeded in 3D hyaluronic acid–RGD hydrogels (*n* = 6) and cultured for 28 days. Afterward, devices were disassembled and microdissected to isolate invasive and core cells for DNA sequencing (Supplemental Figure 1, A and B). sgRNAs enriched in the core versus the invasive fraction (indicating genes whose knockdown disrupted invasion) and in the invasive fraction versus the core (indicating genes whose knockdown enabled invasion) were scored based on abundance versus nontargeting sgRNAs in the library (Supplemental Table 2). Of the 2,550 genes screened, 12 genes showed significant enrichment of targeting sgRNAs in the core (Figure 2A and Supplemental Table 2): (a) RIO kinase 2 (*RIOK2*; average phenotype of strongest 3 = –4.6; *P* = 0.002), an ATPase contributing to cell growth and protein synthesis in oral squamous cell carcinoma (10); (b) 6-phosphofructokinase (*PFKL*; average phenotype of strongest 3 = –3.56; *P* = 0.004), a glycolytic enzyme regulating lipolysis (11); (c) superoxide dismutase type 1 (*SOD1*; average phenotype of strongest 3 = –2.11; *P* = 0.0003), an antioxidant enzyme promoting proliferation and invasion in lung cancer (12); (d and e) NADH ubiquinone oxidoreductase subunit A2 (*NDUFA2*; average phenotype of strongest 3 = –2.1; *P* = 0.002) and *NDUF* core subunit V2 (*NDUFV2*; average phenotype of strongest 3 = –1.88; *P* = 0.0006), both elements of the electron transport chain; (f) phenylalanyl-tRNA synthetase β chain (*FARSB*; average phenotype of strongest 3 = –3.97; *P* = 0.003), which activates the mTORC1 signaling pathway, facilitating hepatocellular carcinoma progression (13); (g) monopolar spindle 1 (Mps1) kinase (*TTK*; average phenotype of strongest 3 = –6.6; *P* = 0.04), previously implicated in breast cancer metastasis (14); (h) aurora kinase B or aurora B (*AURKB*; average phenotype of strongest 3 = –3.66; *P* = 0.002), which mediates chemoresistance in GBM (15); (i) LMW-PTP/ACP1 (*ACP1*; average phenotype of strongest 3 = –2.98; *P* = 0.008), which mediates chemotherapy resistance and migration in colorectal cancer cells (16); (j) ATPase H^+^ transporting V1 subunit B2 (*ATP6V1B2*; average phenotype of strongest 3 = –3.6; *P* = 0.008), which mediates intracellular organelle acidification without previously defined oncological roles; (k) lipoic acid synthetase (*LIAS*; average phenotype of strongest 3 = –2.6; *P* = 0.008), which promotes apoptosis in lung adenocarcinoma cells (17); and (l) growth factor, ERV1-like (*GFER;* average phenotype of strongest 3 = –3.3; *P* = 0.001), implicated in the human mitochondrial disulfide relay system without known oncological function.

We performed single-gene knockdowns on these genes to validate their contributions to slowing invasion. Substantive knockdown was achieved for 8 of the 12 candidates using single gene–targeting CRISPRi (Supplemental Figure 2A). Compared with control GBM43 cells expressing dCas9, only 2 of 8 knockdown cell lines, those targeting *AURKB* or *ACP1,* exhibited decreased invasion in complementary validatory spheroid assays performed over 4 days in hyaluronic acid–RGD hydrogels (*P* < 0.0001; Figure 2, B and C). The 2 knockdown lines exhibited no viability changes after 72 hours in 2D culture (Supplemental Figure 2B).

### Validation of druggable genome CRISPRi hits with pharmacological targeting.

We then assessed the effect of pharmacologically inhibiting the proteins encoded by *AURKB and ACP1* on GBM43 spheroid invasion. We chose 2 previously investigated anticancer agents (18, 19): AZD1152-HQPA (barasertib), a ligand with 1,000-fold selectivity for aurora kinase B over A (20), and LMW-PTP inhibitor I (MLS-0322825 Cmpd23), an inhibitor of *ACP1*’s product LMW-PTP with over 50-fold preference for LMW-PTP over a large panel of PTPs (21). Treatment of GBM43 spheroids with AZD1152-HQPA or LMW-PTP inhibitor I at the highest nontoxic concentrations (Supplemental Figure 2C) reduced invasion at the 4-day time point used for KO cells (*P* = 0.01 AZD1152, *P* = 0.006 LMW-PTP inhibitor I, Figure 2D). Daily assessment of GBM43 spheroids treated with AZD1152-HQPA or LMW-PTP inhibitor I revealed comparable time course, with both first slowing invasion on day 3 (*P* < 0.05) in a robust and durable manner from day 4 until spheroids filled the field on day 6 (*P* < 0.0001). AZD1152-HQPA and LMW-PTP inhibitor I dramatically slowed invasion over 6 days (*P* < 0.0001; Figure 2E). Combined treatment of GBM43 spheroids with AZD1152-HQPA and LMW-PTP inhibitor I slowed invasion more than either agent alone (*P* < 0.0001; Supplemental Figure 2D), suggesting a complementary effect. These findings were corroborated in 2 additional GBM patient-derived xenograft lines: GBM102 (MGMT hypermethylated/classical subtype) and GBM28 (MGMT unmethylated/mesenchymal subtype) (9). Treating these 2 lines with AZD1152-HQPA (*P* < 0.0001 GBM102; *P* = 0.0004 GBM28) or LMW-PTP inhibitor I (*P* = 0.02 GBM102; *P* = 0.02 GBM28) slowed 3D spheroid invasion (Supplemental Figure 2, E and F).

### Proximity labeling assays implicate cortactin as a shared target of both aurora B and LMW-PTP in GBM.

**Figure 3:**
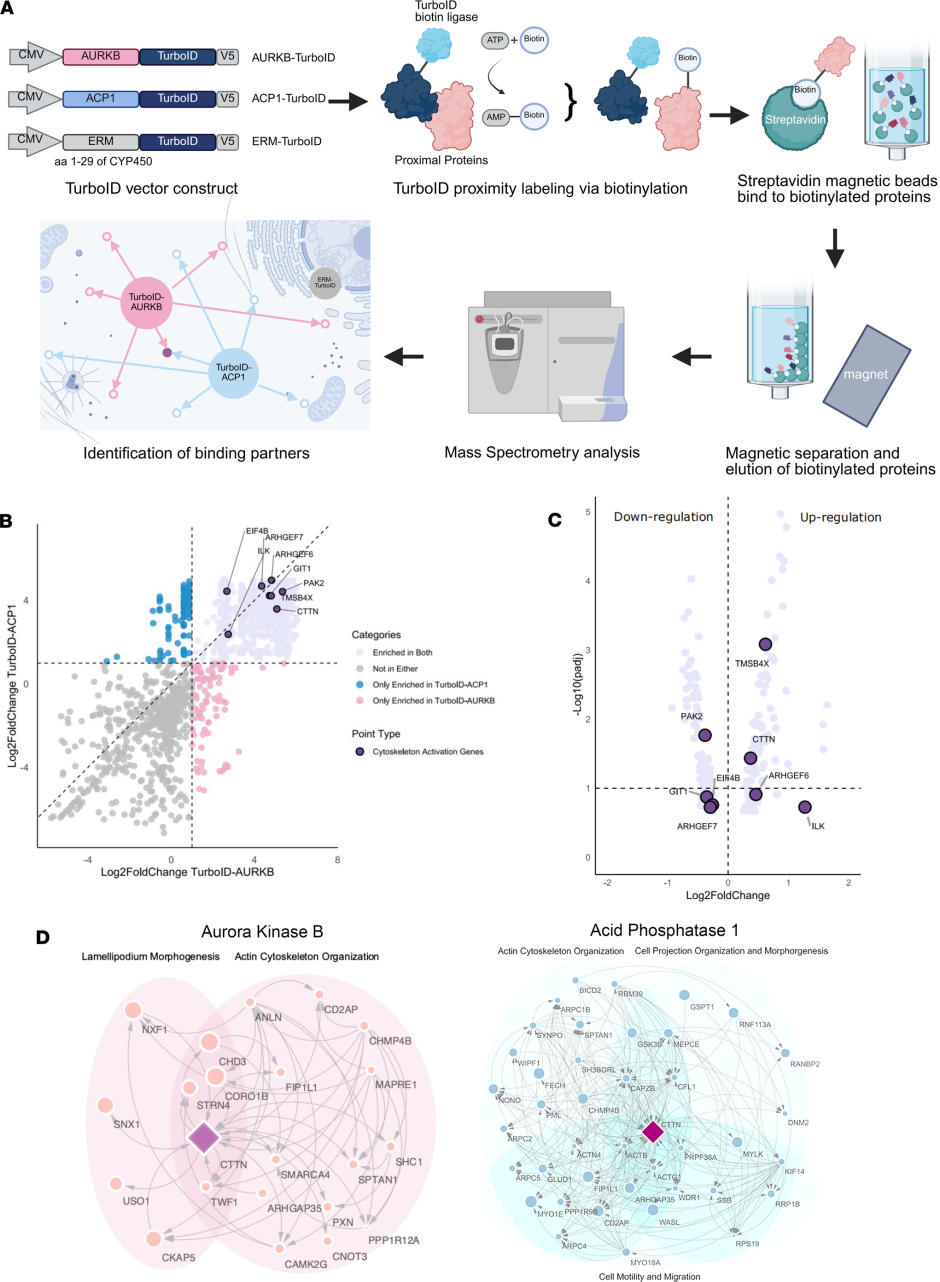
Proximity labeling identifies cortactin as a binding partner for aurora B and LMW-PTP, and a key regulator of GBM invasion, whose expression increases in invasive GBM cells. (**A**) Workflow for TurboID *AURKB* and *ACP1*. *AURKB*, *ACP1*, and control sequence encoding aa 1–29 of cytochrome P450 were fused to TurboID sequence, an engineered biotin ligase that converts biotin into biotin-AMP, covalently labeling proximal proteins. Biotinylated proteins were isolated using streptavidin magnetic beads and analyzed using mass spectrometry to identify binding partners. Created in BioRender. Lovalvo I. (2026) (https://BioRender.com/xudh77w). (**B**) Proteins bound to aurora B (*AURKB)* or LMW-PTP (*ACP1*) based on log_2_fold change of binding to aurora B versus cytochrome P450 (*x* axis) and the log_2_fold change of binding to LMW-PTP versus cytochrome P450 (*y* axis). Gray proteins did not significantly bind to either protein relative to cytochrome P450. Cyan proteins significantly bind to LMW-PTP. Pink proteins significantly bind to aurora B. Light purple proteins significantly bind to both targets versus control. The 8 proteins identified are part of the invasion-related KEGG pathways highlighted in **C**. (**C**) Genes for proteins significantly binding aurora B and LMW-PTP, stratified by gene expression in invasive versus core fractions of 3D hydrogels. (**D**) CellSCAPE analysis of cortactin (indicated by a purple diamond) interactions with known binding partners of aurora B (left) and LMW-PTP (right).

To understand how *AURKB* and *ACP1* drive GBM invasion, we sought to identify binding partners of their protein products, aurora B and LMW-PTP, that could enable them to drive GBM invasion. We performed proximity labeling through biotinylation and mass spectrometry (MS), an unbiased technique for capturing protein-protein interactions within living cells. We used the TurboID system (22), which fuses the protein of interest to a biotin ligase, using ATP to convert biotin into biotin-AMP, which covalently labels proximal proteins. We performed streptavidin bead pulldown of lysates from GBM43 cells expressing TurboID aurora B and LMW-PTP fusion proteins and a TurboID cytochrome P450 fusion protein (Figure 3A), followed by MS analysis of pulled-down biotinylated proteins. Of 2,814 proteins identified in pulldowns, 903 and 1,437 bound to TurboID aurora B and LMW-PTP fusion proteins, and had greater than 2-fold more abundance in aurora B and LMW-PTP TurboID pulldowns versus control TurboID cytochrome P450 fusion protein. Of these significantly enriched proteins, 703 were shared binding partners of aurora B and LMW-PTP (Figure 3B, Supplemental Figure 3, A and B, and Supplemental Table 3). Among enriched KEGG pathways in these binding proteins, 3 regarded cancer invasion: proteoglycans in cancer, actin cytoskeleton regulation, and focal adhesion (Supplemental Figure 3C). Among 703 proteins interacting with both targets, 8 were found in these 3 invasion-relevant KEGG pathways: eukaryotic translation initiation factor 4B (*EIF4B*), integrin-linked kinase (*ILK*), Rac/Cdc42 guanine nucleotide exchange factor 6 (*ARHGEF6*), rho guanine nucleotide exchange factor 7 (*ARHGEF7*), G protein–coupled receptor kinase interactor-1 (*GIT1*), p21 protein-activated kinase 2 (*PAK2*), thymosin-β4 (*TMSB4X*), and cortactin (*CTTN*) (Figure 3B). We then refined this list by identifying which of these genes were upregulated in our previous RNA-Seq of invasive versus core GBM43 cells invading 3D hydrogels (23) resulting in 2 genes, each encoding actin-binding proteins: *TMSB4X* and *CTTN* (Figure 3C). Because thymosin-β4 (encoded by *TMSB4X*) only has serine and threonine phosphorylation sites (hence only actable on by aurora B), while cortactin (encoded by *CTTN*) has serine, threonine, and tyrosine phosphorylation sites (24) that could be acted on by aurora B and LMW-PTP, we focused on cortactin (Supplemental Figure 3D). Cortactin is an actin-binding protein with 2 known roles in cell morphology and migration: (a) regulating interactions between components of adherens-type junctions and (b) organizing the actin cytoskeleton and cell adhesion structures, like lamellipodia, in epithelial and cancer cells, including GBM cells (25). Further rationale for focusing on cortactin emerged from in silico analysis performed using Cytoscape, an open-source database of protein-protein interaction networks, revealing cortactin interactions with numerous proteins that interact with aurora B and LMW-PTP (Figure 3D), and from 3D spheroid assays in which *CTTN* knockdown (Supplemental Figure 3E) reduced GBM43 invasion (Supplemental Figure 3F) without altering proliferation (Supplemental Figure 3G), similar to *AURKB* or *ACP1* knockdown.

### AURKB and ACP1 regulate cortactin phosphorylation in GBM.

**Figure 4:**
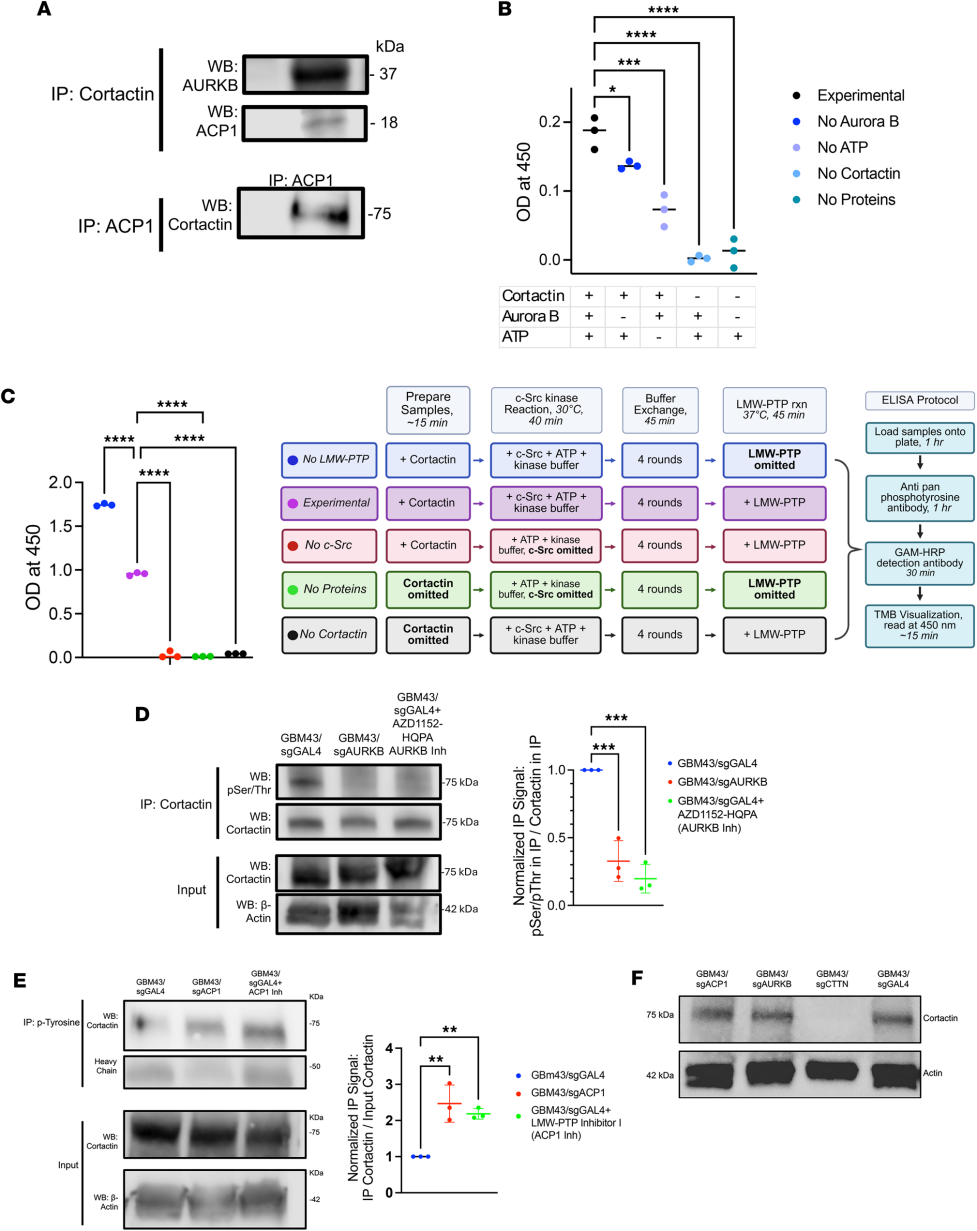
Serine phosphorylation of cortactin by aurora B (*AURKB)* and tyrosine dephosphorylation of cortactin by LMW-PTP (*ACP1)*. (**A**) IP of cortactin from GBM43 cell lysates, then blotting for aurora B (upper row) and LMW-PTP (middle row) confirmed cortactin binds both proteins. Given the lower intensity of the LMW-PTP band in immune precipitate relative to aurora B, binding of LMW-PTP to cortactin was verified by precipitating LMW-PTP from the same lysates and blotting for cortactin (lower row). *n* = 3 technical replicates. (**B**) A sandwich ELISA used an anti-cortactin capture antibody and an anti-phosphorylated serine/threonine detection antibody. Incubating recombinant cortactin with aurora B and ATP revealed increased serine/threonine phosphorylation of cortactin (*P* < 0.05) versus reactions without aurora B. Performed with 3 technical replicates. (**C**) A sandwich ELISA used an anti-cortactin capture antibody and an anti-phosphorylated tyrosine detection antibody. Incubating recombinant cortactin with c-Src and ATP led to increased tyrosine phosphorylation of cortactin (*P* < 0.0001) versus reactions without c-Src (Supplemental Figure 4A). Incubating this tyrosine-phosphorylated cortactin with LMW-PTP decreased tyrosine phosphorylation of cortactin (*P* < 0.0001) versus reactions without LMW-PTP; the schematic above explains each group’s reactions. *n* = 3 technical replicates. Created in BioRender. (**D**) Targeting *AURKB* with CRISPRi in GBM43/sgAURKB cells or via AZD1152-HQPA revealed reduced cortactin serine phosphorylation, determined by IP of cortactin, blotting for phosphorylated serine/threonine (first row) and for cortactin (second row) or blotting the lysates without precipitation for cortactin (third row) or β-actin (fourth row). Quantification resulted from band intensities in 3 technical replicates in Supplemental Figure 4B. (**E**) Targeting *ACP1* with CRISPRi in GBM43/sgACP1 cells or via LMW-PTP inhibitor I revealed increased cortactin tyrosine phosphorylation, determined by IP for phosphorylated tyrosine, blotting for cortactin (first row), and blotting for heavy chain antibody (second row), as well as blotting the non-precipitated lysates for cortactin (third row) and β-actin (fourth row). Quantification resulted from band intensities in 3 technical replicates in Supplemental Figure 4C. (**F**) Western blot for total cortactin levels confirmed *CTTN* knockdown in GBM43/sgCTTN cells versus GBM43/sgGAL4 control cells and revealed decreased cortactin levels in GBM43/sgAURKB cells relative to control cells, determined by quantification of band intensities in 3 technical replicates in Supplemental Figure 4D. *P < 0.05; **P < 0.01; ***P < 0.001; ****P < 0.0001. One-way ANOVA with Tukey’s pairwise post hoc comparisons. Scatter dot plots show mean (horizontal bar) with SD (vertical bar).

We interrogated interactions between cortactin and the aurora B and LMW-PTP proteins. We confirmed using IP that cortactin bound aurora B and LMW-PTP (Figure 4A). We then established sandwich ELISA reactions to detect phosphorylated cortactin using a cortactin capture antibody and phosphorylated serine/threonine or tyrosine detection antibodies. We found that incubating recombinant cortactin and aurora B led to increased serine/threonine phosphorylation of cortactin (*P* < 0.05; Figure 4B). A search of all serine and threonine phosphorylation sites in cortactin (http://www.phosphosite.org) revealed that 2 of 36 potential serine phosphorylation sites in cortactin had moderate to high scores as aurora B phosphorylation sites: serine 81 (26) (log_2_(score) = 1.242, site percentile = 91.43, percentile rank = 23) and serine 109 (27) (log_2_(score) = 0.9846, site percentile = 90.55, percentile rank = 30). We found that incubating cortactin with c-Src, a tyrosine kinase previously shown to phosphorylate cortactin (28), increased tyrosine phosphorylation of cortactin (*P* < 0.0001; Supplemental Figure 4A); subsequent incubation of tyrosine-phosphorylated cortactin with LMW-PTP decreased tyrosine phosphorylation of cortactin (*P* < 0.0001; Figure 4C).

To determine whether aurora B-driven serine/threonine phosphorylation and LMW-PTP–driven tyrosine dephosphorylation of cortactin also occurred intracellularly, we immunoprecipitated cortactin and blotted with an antibody targeting phosphorylated serine/threonine residues to show that GBM43 cells with *AURKB* knockdown (*P* = 0.0004) or treated with AZD1152-HQPA (*P* = 0.0002) exhibited decreased serine/threonine phosphorylation of cortactin (Figure 4D and Supplemental Figure 4B), consistent with aurora B acting as a serine/threonine kinase on cortactin intracellularly. We then investigated tyrosine phosphorylation of cortactin in GBM43 cells with *ACP1* knockdown or with LMW-PTP inhibitor I treatment, which (*P* = 0.008) increased tyrosine phosphorylation of cortactin (Figure 4E and Supplemental Figure 4C). Analysis of total cortactin levels by Western blot in cells revealed that GBM43/sgAURKB cells (*P* = 0.01) had less cortactin than control GBM43/sgGAL4 cells (Figure 4F and Supplemental Figure 4D), suggesting that cortactin’s phosphorylation status affected its stability.

### Cortactin drives actin polymerization in GBM lamellipodia and associated morphological changes based on its AURKB/ACP1-regulated phosphorylation.

**Figure 5:**
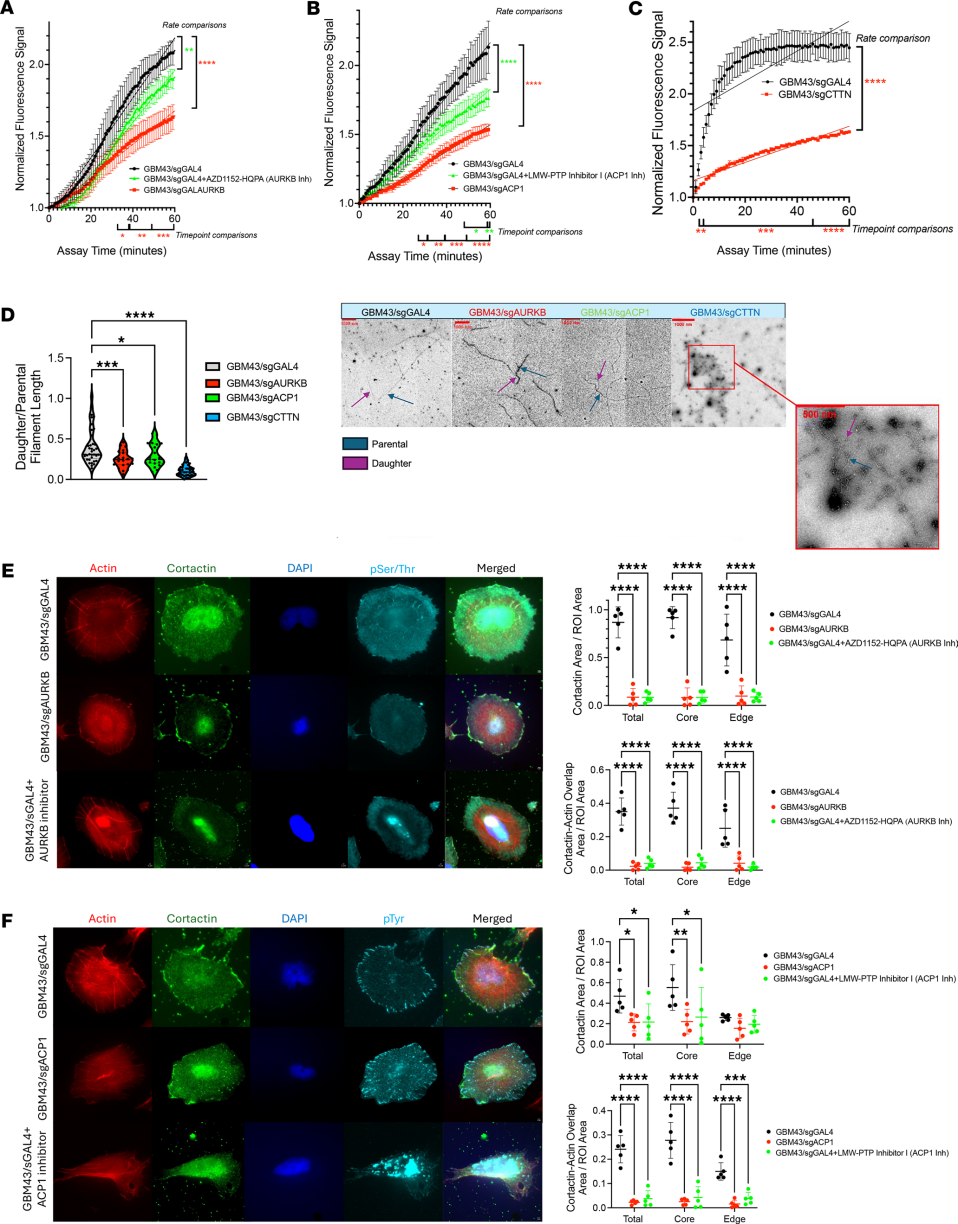
*AURKB* and LMW-PTP (*ACP1*) affect cortactin phosphorylation, altering actin polymerization, cortactin levels, and actin-cortactin overlap. (**A**) Lysates from GBM43/sgGAL4 cells treated with AZD1152-HQPA (P =0.003) and GBM43/sgAURKB cells (*P* <0.0001) slowed polymerization rate versus GBM43/ sgGAL4 lysates. Time point differences occurred for GBM43/sgAURKB lysates versus GBM43/sgGAL4 lysates (red asterisks). Polymerization between lysates of GBM43/sgGAL4 cells+AZD1152-HQPA versus GBM43/sgGAL4 lysates was not significant. *n* = 3/group. Points, means; data shown as mean ± SEM. (**B**) Lysates from GBM43/sgGAL4 cells+LMW-PTP inhibitor I (*P* < 0.0001) and GBM43/sgACP1 cells (*P* < 0.0001) slowed polymerization rate versus GBM43/sgGAL4 lysates. Time point differences occurred for GBM43/sgACP1 lysates versus GBM43/sgGAL4 lysates (red asterisks). Green asterisks, polymerization differences between lysates of GBM43/sgGAL4 cells+LMW-PTP inhibitor I versus GBM43/sgGAL4 lysates. *n* = 3/group. Points, means; data shown as mean ± SEM. (**C**) GBM43/sgCTTN lysates slowed polymerization rate versus GBM43/ sgGAL4 lysates (*P* < 0.0001). No differences in polymerization at individual time points occurred but might eventually occur given polymerization rates. *n* = 3/group. (**D**) Reactions from A–C visualized by electron microscopy (EM), with daughter/parental filament length ratio a branching marker. Lysates from GBM43/sgAURKB (*P* = 0.0009), GBM43/sgACP1 (P = 0.03), and GBM43/sgCTTN (*P* < 0.0001) cells lowered ratio versus control GBM43/sgGAL4 cells. *n* = 21–37 parent filaments/group. Right: EM images with cyan parent filament and purple daughter filament; original magnification, ×6,500; scale bar: 1,000 nm for 4 main photos, 500 nm for zoomed-in photo demonstrating diminished branching. Violin plots show all points, horizontal bars at median and quartiles. (**E**) Targeting AURKB with CRISPRi or drug (*n* = 5/group) reduced cortactin levels (*P* < 0.0001, upper graph) and cortactin-actin overlap (*P* < 0.0001; lower graph) normalized to cell area. Original magnification, ×60; scale bar: 2 mm. (**F**) Targeting ACP1 with CRISPRi or drug (*n* = 5/group) reduced total (*P* = 0.042 CRISPRi and P = 0.045 inhibitor) and core region (*P* = 0.006 CRISPRi and *P* = 0.02 inhibitor) but not cell edge (*P* = 0.6–0.8) cortactin immunostaining, reduced cortactin-actin overlap (*P* < 0.0001 total, core, and edge) normalized to cell area. Original magnification, ×60; scale bar: 2 mm. Scatter dot plots show mean (horizontal bar) with SD (vertical bar) (**E**–**F**). *P < 0.05; **P < 0.01; ***P < 0.001; ****P < 0.0001. Linear regression for slope comparisons (**A**–**C**), t test for time point comparisons (**A**–**C**), and 1-way ANOVA with Tukey’s pairwise post hoc comparisons (**D**–**F**).

Given cortactin’s role in actin polymerization (29), we investigated the impact of targeting *AURKB*, *ACP1*, or *CTTN* on actin polymerization using an in vitro assay where lysates of GBM43 cells with *AURKB*, *ACP1*, or *CTTN* knockdown were incubated with pyrene-conjugated actin, which exhibits increased fluorescence in its F-actin form versus its monomeric G-actin form, increasing fluorescent signal during actin polymerization. We found that *AURKB* (Figure 5A) or *ACP1* (Figure 5B) knockdown or pharmacological targeting reduced actin polymerization rates (*P* < 0.0001) and levels. Similarly, *CTTN* knockdown in GBM43 cells reduced actin polymerization rates (*P* < 0.0001) and levels compared with GBM43/sgGAL4 control cells (Figure 5C). Adding recombinant cortactin elevated rates (*P* < 0.0001) and levels of actin polymerization in cells with *AURKB*, *ACP1*, or *CTTN* knockdown without affecting actin polymerization in GBM43/sgGAL4 control cells (Supplemental Figure 5A). Moreover, electron microscopy revealed decreased branching, assessed by the ratio of daughter to parental filament length, of actin filaments incubated with lysate from knockdown of GBM43 cells with *AURKB* (*P* = 0.0009), *ACP1* (*P* = 0.03), or *CTTN* (*P* < 0.0001) compared with lysate from control GBM43/sgGAL4 cells (Figure 5D).

These effects facilitated morphological changes in cultured GBM43 cells; *AURKB* or *ACP1* knockdown lowered aspect ratio, a morphological metric elevated in elongated spindle-like cells more adept at invasion compared with rounder cells (30) (*P* < 0.0001; Supplemental Figure 5B). Additionally, GBM43 cells with *AURKB* (*P* < 0.0001) or *ACP1* (*P* = 0.002) knockdown displayed smaller starting volume than control GBM43/sgGAL4 cells upon encapsulation in 3D hydrogels (Supplemental Figure 5C). We also found that GBM43 cells with *AURKB* (*P* = 0.007) or *ACP1* (*P* < 0.0001) knockdown had proportionally smaller nuclear areas (Supplemental Figure 5D), reducing the elevated nuclear to cytoplasmic ratio that characterizes malignant cells (31), including GBM43 cells (32). This cellular contraction seen with *AURKB* or *ACP1* knockdown did not portend cell death; the frequency of viable nonapoptotic cells remained comparably high in cultured GBM43 cells with knockdown of *AURKB* (*P* = 0.1), *ACP1* (*P* = 0.7), or *CTTN* (*P* = 0.3) compared with GBM43/sgGAL4 cells (Supplemental Figure 5E).

### Cortactin phosphorylation changes associated with AURKB or ACP1 targeting lead to reduced levels of cortactin and Arp3.

Given the importance of cellular localization of actin and cortactin for lamellipodia formation at the edge of invading cancer cells, we used immunofluorescent analysis of cultured cells to determine any regional intracellular variations in the phosphorylated cortactin changes noted by Western blot with *AURKB* or *ACP1* targeting in GBM43 cells (Figure 4, D and E). We found that diminished cortactin serine phosphorylation achieved with *AURKB* knockdown or aurora B drug treatment was demonstrable by immunostaining throughout the cell core or within 3 μm of the cell edge (*P* < 0.0001 with CRISPRi or drug in total cell and cell core; *P* = 0.008 with CRISPRi, *P* = 0.007 with drug within 3 mm of the cell edge; Supplemental Figure 5F). Conversely, increased tyrosine phosphorylation from *ACP1* knockdown or pharmacological targeting of LMW-PTP was demonstrated in total throughout the cell (*P* = 0.01 with CRISPRi, *P* = 0.0005 with inhibitor) and in the cell core (*P* = 0.01 with CRISPRi, *P* < 0.0001 with inhibitor), but with no change within 3 mm of the cell edge (*P* = 0.1 with CRISPRi, *P* = 0.06 with inhibitor) due to a high background level of cortactin tyrosine phosphorylation at the cell edge (Supplemental Figure 5G). We found that aurora B targeting by *AURKB* knockdown or AZD1152 drug treatment reduced total cellular cortactin levels in both the core and edge (*P* < 0.0001; Figure 5E), while LMW-PTP targeting by *ACP1* knockdown or LMW-PTP inhibitor I drug treatment only reduced cortactin at the cell core (*P* = 0.006 CRISPRi and *P* = 0.02 inhibitor), not the cell edge (*P* = 0.6–0.8; Figure 5F), consistent with regions experiencing increased cortactin tyrosine phosphorylation with LMW-PTP targeting.

Given these effects on actin polymerization, we performed immunofluorescent analysis of cultured cells to investigate the impact of aurora B or LMW-PTP targeting on cellular actin-cortactin colocalization. We found that aurora B or LMW-PTP targeting with gene knockdown or drug treatment disrupted actin-cortactin colocalization throughout the cell core and edge (*P* < 0.0001; Figure 5, E and F), suggesting that alterations in cortactin’s phosphorylation status from targeting aurora B or LMW-PTP affected cortactin-actin binding, likely explaining the reduced actin polymerization noted when targeting aurora B or LMW-PTP. We then investigated the role of the Arp2/3 complex containing actin-related protein 2 and 3 (Arp2 and Arp3), recently implicated as a mediator of cortactin-induced actin stabilization (33), in actin stabilization by cortactin when it is serine phosphorylated/tyrosine dephosphorylated. Immunostaining for Arp3, the component of the complex binding cortactin (33), revealed decreased intracellular Arp3 levels in GBM43 cells with *AURKB* (*P* = 0.03 whole cell; *P* = 0.02 cytoplasm; *P* = 0.1 nuclei) or *ACP1* (*P* < 0.0001 in whole cell, cytoplasm, or nuclei) knockdown compared with control GBM43/sgGAL4 cells (Supplemental Figure 5H). GBM43 cells with *AURKB* or *ACP1* knockdown also exhibited decreased Arp3-cortactin overlap (*P* < 0.0001; Supplemental Figure 5H) and decreased Arp3-actin overlap (*P* = 0.009 *AURKB*, *P* = 0.0003 *ACP1*; Supplemental Figure 5H) versus control GBM43/sgGAL4 cells, demonstrating downstream effects of changes in cortactin phosphorylation associated with *AURKB* or *ACP1* targeting.

### AURKB or ACP1 targeting leads to actin redistribution and decreased mesenchymal gene expression.

We found that *AURKB* or *ACP1* knockdown shifted actin distribution in cultured GBM43 cells from even distribution between the nucleus and cytoplasm in GBM43/sgGAL4 cells (*P* = 0.9) heavily toward the nucleus (*P* = 0.002–0.004; Supplemental Figure 5I), which was intriguing given recent findings that nuclear to cytoplasmic actin shifting in cancer cells may facilitate invasion by altering the cell’s mechanical properties and influencing gene expression through modulated chromatin accessibility (34). Consistent with potential transcriptional effects of this shift in cellular actin distribution, *AURKB* or *CTTN* knockdown in GBM43 cells lowered expression of mesenchymal GBM transcriptional signature genes *FOXC1* (*P* = 0.0007 *AURKB*, *P* = 0.04 *CTTN*), *FOXC2* (*P* < 0.0001 *AURKB*, *P* = 0.0007 *CTTN*) *SLUG* (*P* < 0.0001 *AURKB* and *CTTN*), *SNAIL* (*P* = 0.0008 *AURKB*, *P* = 0.0003 *CTTN*), and *ZEB2* (*P* = 0.0003 *AURKB*, *P* = 0.01 *CTTN*) compared with control GBM43/sgGAL4 cells, while *ACP1* knockdown lowered expression of *SLUG* (*P* < 0.0001) and *FOXC1* (*P* = 0.009) compared with control GBM43/sgGAL4 cells (Supplemental Figure 5J).

### AURKB and ACP1 mediate distinct aspects of GBM invasion.

We demonstrated shared functionality between aurora B and LMW-PTP in cultured GBM cells, like stabilizing cortactin to promote colocalization with Arp2/3 and actin, promoting actin polymerization, and rendering pro-invasive morphological and transcriptional alterations. The potent anti-invasive effects when targeting both aurora B and LMW-PTP compared with either alone (Supplemental Figure 2C) prompted us to identify complementary aspects of GBM cell invasion affected differently by these 2 proteins.

**Figure 6:**
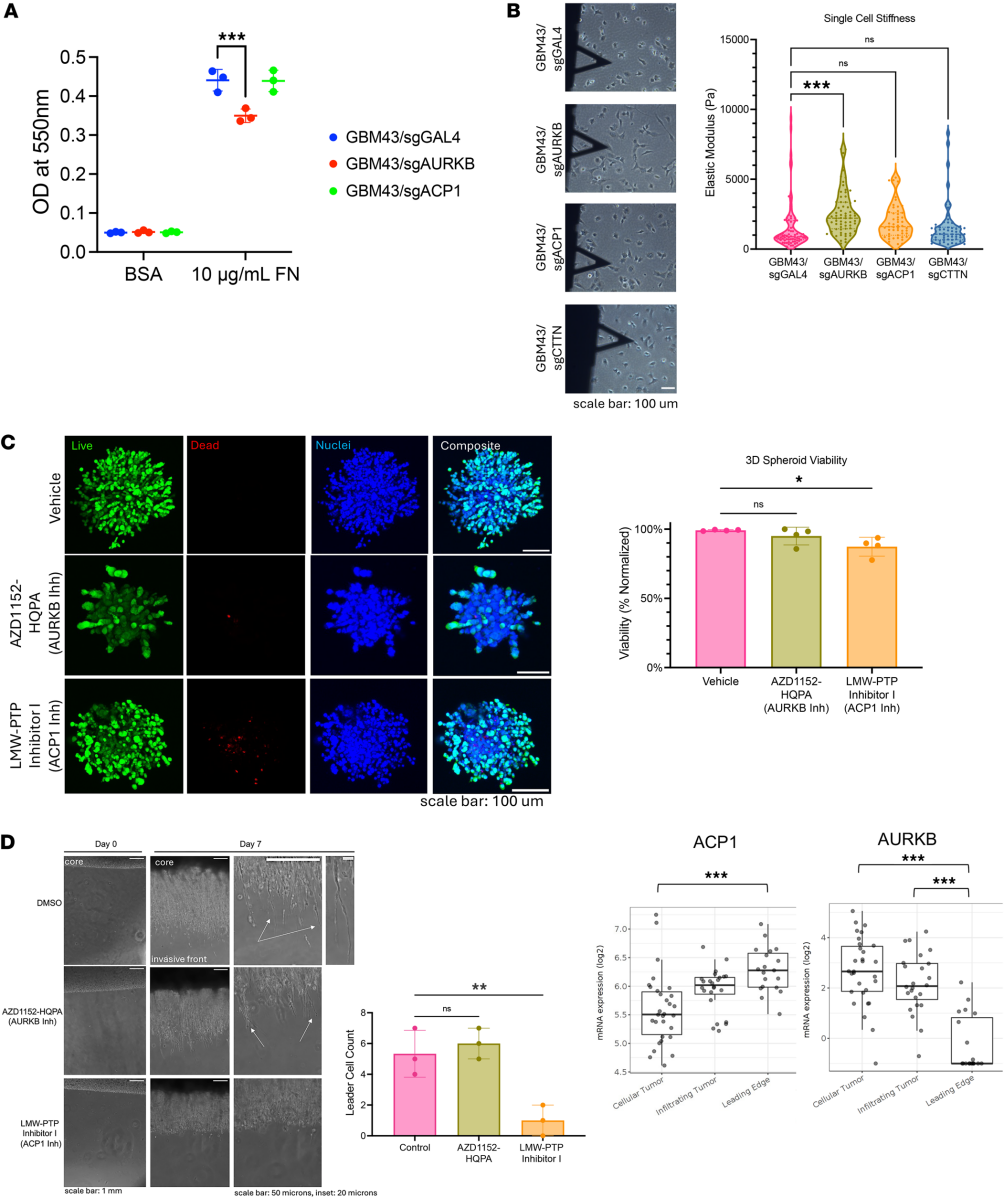
*AURKB* and *ACP1* exert distinct effects on different aspects of GBM invasion. (**A**) GBM43/sgAURKB cells exhibited reduced fibronectin adhesion versus GBM43/sgGAL4 (*P* = 0.0001), while GBM43/sgACP1 cells had unchanged fibronectin adhesion (*P* = 0.9) (*n* = 3/group). (**B**) Atomic force microscopy revealed decreased single-cell stiffness in GBM43/sgAURKB cells versus GBM43/sgGAL4 cells (*P* = 0.0006), no changes in GBM43/sgACP1 or GBM43/sgCTTN cells (*n* = 50–75 individual cells/condition). Original magnification, ×10; scale bar: 100 μm. (**C**) Live/dead cell staining revealed that inhibiting LMW-PTP (*ACP1*) with LMW-PTP inhibitor I decreased cell viability/increased cell death in GBM43 cells attempting to invade during neurosphere assays (*P* = 0.04), with no changes with aurora B inhibitor AZD1152-HQPA (*n* = 4 spheres/condition). Original magnification, ×10; scale bar: 100 μm. (**D**) Light microscopy revealed that, in U87 GBM cells, inhibition with LMW-PTP inhibitor I altered (*P* = 0.008) leader/follower dynamics identified in 3D hydrogel assays; no changes noted with aurora B inhibitor AZD1152-HQPA (*P* = 0.7) based on counting leader cell number across 3 biological replicates. Original magnification, ×10; scale bar for day 0 and leftmost day 7 images:1 mm; scale bar for rightmost day 7 images: 50 μm; inset scale bar: 20 μm. (**E**) *ACP1* expression increased from core cellular tumor to infiltrating tumor to leading edge (*P* = 4.4 × 10^–6^ cellular tumor versus leading edge), while *AURKB* expression decreased from core cellular tumor to infiltrating tumor to leading edge (*P* = 4.4 × 10^–13^ cellular tumor versus leading edge; *P* = 4.9 × 10^–9^ infiltrating tumor versus leading edge), assessed using the Ivy Glioblastoma Atlas Project, which transcriptomically profiled site-directed biopsies in newly diagnosed GBMs from 10 patients. ns, not significant; *P < 0.05; **P < 0.01; ***P < 0.001. One-way ANOVA with Tukey’s pairwise post hoc comparisons. Scatter dot plots show mean (horizontal bar) with standard deviation (vertical bar). For box and whisker plots, horizontal line in the box is the median; box extends from 25th to 75th percentile and whiskers from minimum to maximum value.

We first investigated adhesion to ECM, the critical first step in invasion. Compared with GBM43/sgGAL4 control cells, GBM43 cells with *AURKB* knockdown exhibited decreased adhesion to fibronectin (*P* = 0.0001), a critical component of the GBM ECM (35), while GBM43 cells with *ACP1* knockdown had unchanged adhesion to fibronectin (*P* = 0.9; Figure 6A). We found that GBM43 cells with *AURKB* but not *ACP1* knockdown exhibited increased individual cell stiffness measured by atomic force microscopy (*P* = 0.0006; Figure 6B), suggesting a role for aurora B in reducing cell stiffness, required for cancer cells to squeeze through tight spaces during invasion (36).

Conversely, GBM43 cells treated with LMW-PTP inhibitor I, but not those treated with aurora B inhibitor AZD1152-HQPA, exhibited cell death in the core of tumor spheroid 3D neurosphere assays (*P* = 0.04; Figure 6C), sometimes observed in this platform when cells cannot escape the tumor to access nutrients. This suggests that LMW-PTP may facilitate GBM cells exiting the tumor core. Conversely, tumor spheroids treated with AZD1152-HQPA were slower to invade than control, but did not experience cell death, suggesting that AZD1152-HQPA slowing of invasion occurs after initial egress from the tumor core. To corroborate the role of LMW-PTP in GBM cell launch in a context more sensitive to individual cell migration, we investigated its role in leader/follower dynamics we previously identified through encapsulating U87 GBM cells (MGMT methylated/proneural subtype) in high molecular weight (HMW) hyaluronic acid formulation 3D hydrogels (Supplemental Figure 6, A and B) (37), in which GBM cells invade rapidly, characterized by leader cells extending long protrusions that bifurcate to sense the approaching matrix. As hypothesized, pharmacological inhibition of the *ACP1* gene product with LMW-PTP inhibitor I (*P* = 0.008), but not aurora B with AZD1152-HQPA (*P* = 0.7), in U87 GBM cells disrupted leader/follower mechanics, evidenced by counting leader cells invading HMW 3D hydrogels (Supplemental Figure 6C and Figure 6D) and supported by time-lapse videos of HMW 3D hydrogel invasion, revealing that U87 GBM cells treated with LMW-PTP inhibitor escape the channel (i.e., tumor core) much slower than U87 GBM cells treated with vehicle or AZD1152-HQPA (Supplemental Video 1).

These findings suggested that aurora B supports GBM cells in anchoring themselves and regulating their stiffness, functions attributed to myosin motors (38) and focal adhesions, and LMW-PTP supports cellular ability to withstand external stressors while launching from the core. Human GBM gene expression data analysis from Ivy GAP revealed decreased *AURKB* expression while transitioning from core to edge in patient GBMs, and *ACP1* expression increased during this transition (*P* < 0.0001; Figure 6E), supporting a role for *AURKB* in earlier stages of invasion and a role for *ACP1* in later stages.

### Effects of AURKB, ACP1, and CTTN on actin remodeling and GBM invasion in vivo.

**Figure 7:**
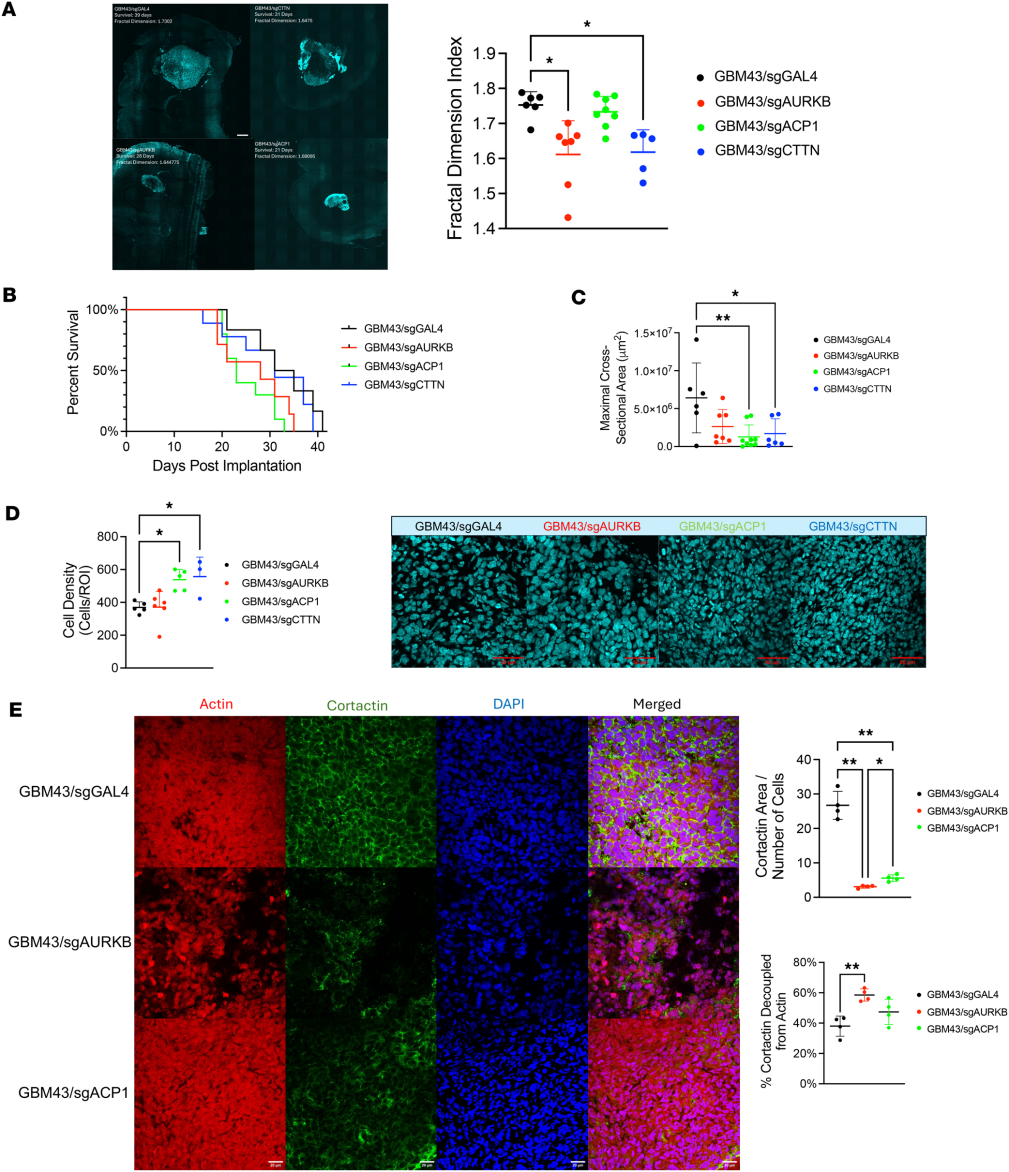
Targeting *AURKB* and *ACP1* in orthotopic GBM xenografts in vivo slows invasion and uncouples cortactin from actin in tumor cells without affecting survival. GBM43/sgGAL4, GBM43/sgAURKB, GBM43/sgACP1, and GBM43/sgCTTN cells were implanted into right frontal lobes of athymic mice and grew until mice reached endpoint. (**A**) GBM43/sgAURKB (*P* = 0.02; *n* = 7) and GBM43/sgCTTN (*P* = 0.02; *n* = 4) xenografts were less invasive than GBM43/sgGAL4 (*n* = 6) in image fractal analysis of tumors and surrounding brain, yielding fractal dimension, a measure of invasive tumor growth between 1 and 2; higher numbers represent greater invasiveness. GBM43/sgACP1 (*n* = 6) xenografts exhibited no fractal dimension change versus GBM43/sgGAL4 xenografts (*P* = 0.8). Image composites at original magnification ×20; scale bar: 500 mm. (**B**) No difference noted in survival of mice with GBM43/sgGAL4, GBM43/sgAURKB, GBM43/sgACP1, and GBM43/sgCTTN xenografts (*P* = 0.6, *n* = 6–10/group). (**C**) Maximal cross-sectional tumor area at endpoint was smaller in GBM43/sgACP1 (*n* = 9, *P* = 0.007) and GBM43/sgCTTN (*n* = 6, *P* = 0.03) xenografts versus GBM43/sgGAL4 (*n* = 6) xenografts, but unchanged in GBM43/sgAURKB (*n* = 7) xenografts versus GBM43/sgGAL4 (*P* = 0.08). One-way ANOVA with Tukey’s pairwise post hoc comparisons. (**D**) Intratumoral cell density, assessed by counting cell number in 3–5 identical fields, was higher in GBM43/sgACP1 (*P* = 0.02) or GBM43/sgCTTN (*P* = 0.03) xenografts versus GBM43/sgGAL4 xenografts, but unchanged in GBM43/sgAURKB (*P* = 0.9) xenografts. *n* = 5 tumors/group, original magnification, ×60; scale bar: 50 mm. (**E**) Xenograft endpoint immunofluorescence revealed decreased immunopositive cortactin area normalized to total cellular tumor area in GBM43/sgAURKB (*P* = 0.003) and GBM43/sgACP1 (*P* = 0.005) xenografts versus GBM43/sgGAL4 xenografts (upper graph) and increased cortactin-actin decoupling in GBM43/sgAURKB (*P* = 0.004) xenografts, but not in GBM43/sgACP1 xenografts (*P* = 0.2) (lower graph). *n* = 4/group, original magnification, ×60; scale bar: 20 mm. ns, not significant; *P < 0.05; **P < 0.01. One-way ANOVA with Tukey’s pairwise post hoc comparisons. Scatter dot plots show mean (horizontal bar) with standard deviation (vertical bar).

We then investigated whether the effects of aurora B, LMW-PTP, or cortactin on GBM invasion in culture could be recapitulated in vivo. Invasiveness in vivo was assessed by fractal analysis of images of tumors and surrounding brain, yielding fractal dimension, a measure of invasive tumor growth between 1 and 2, with higher numbers representing greater invasiveness (5). This revealed that GBM43 cells with *CTTN* (*P* = 0.02) or *AURKB* (*P* = 0.02) knockdown, but not those with *ACP1* knockdown (*P* = 0.8), exhibited decreased invasion in vivo (Figure 7A). Kaplan-Meier analysis revealed that none of the 3 knockdowns altered survival versus GBM43/sgGAL4 control xenografts (*P* = 0.6; Figure 7B), but GBM43/sgACP1 (*P* = 0.007) and SGBM43/sgCTTN (*P* = 0.03) xenografts achieved smaller cross-sectional area than GBM43/sgGAL4 xenografts (Figure 7C), suggesting that although targeting *ACP1* did not affect in vivo invasion as assessed by fractal index in the same manner as *AURKB* targeting, *ACP1* targeting caused an overall tumor contraction that could reflect broader effects on in vivo invasion. We hypothesized that this tumor contraction seen with *ACP1* or *CTTN* knockdown in vivo might not improve survival if instead of causing sufficient cell death, it increased cell density in a smaller space. These denser tumors could increase brain edema from reduced space between cells and the ECM restricting interstitial fluid movement (39), offsetting potential survival gains from smaller tumor volume. Indeed, tumor cell density was higher in xenografts with *ACP1* (*P* = 0.02) or *CTTN* (*P* = 0.03) knockdown versus control GBM43/sgGAL4 xenografts (Figure 7D). These findings suggested distinct benefits of targeting *AURKB* and *ACP1* in vivo, albeit falling short of affecting survival.

We used immunofluorescence to localize cortactin and actin in xenografts with *AURKB* or *ACP1* knockdown. Consistent with our cultured cell findings, *AURKB* (*P* = 0.003) and *ACP1* (*P* = 0.005) knockdown lowered intracellular cortactin levels in GBM43 xenografts (Figure 7E and Supplemental Figure 7A), with *AURKB* or *ACP1* knockdown not altering intracellular cortactin localization (*P* = 0.7–0.9; Supplemental Figure 7B). We found that xenografts with *AURKB* knockdown (*P* = 0.004), but not with *ACP1* knockdown (*P* = 0.2), exhibited disrupted intracellular actin-cortactin colocalization compared with those with *GAL4* control targeting (Figure 7E), aligning with fractal index analysis that had revealed that *AURKB,* but not *ACP1* knockdown, affected microscopic in vivo invasion at the tumor border.

## Discussion

**Figure 8:**
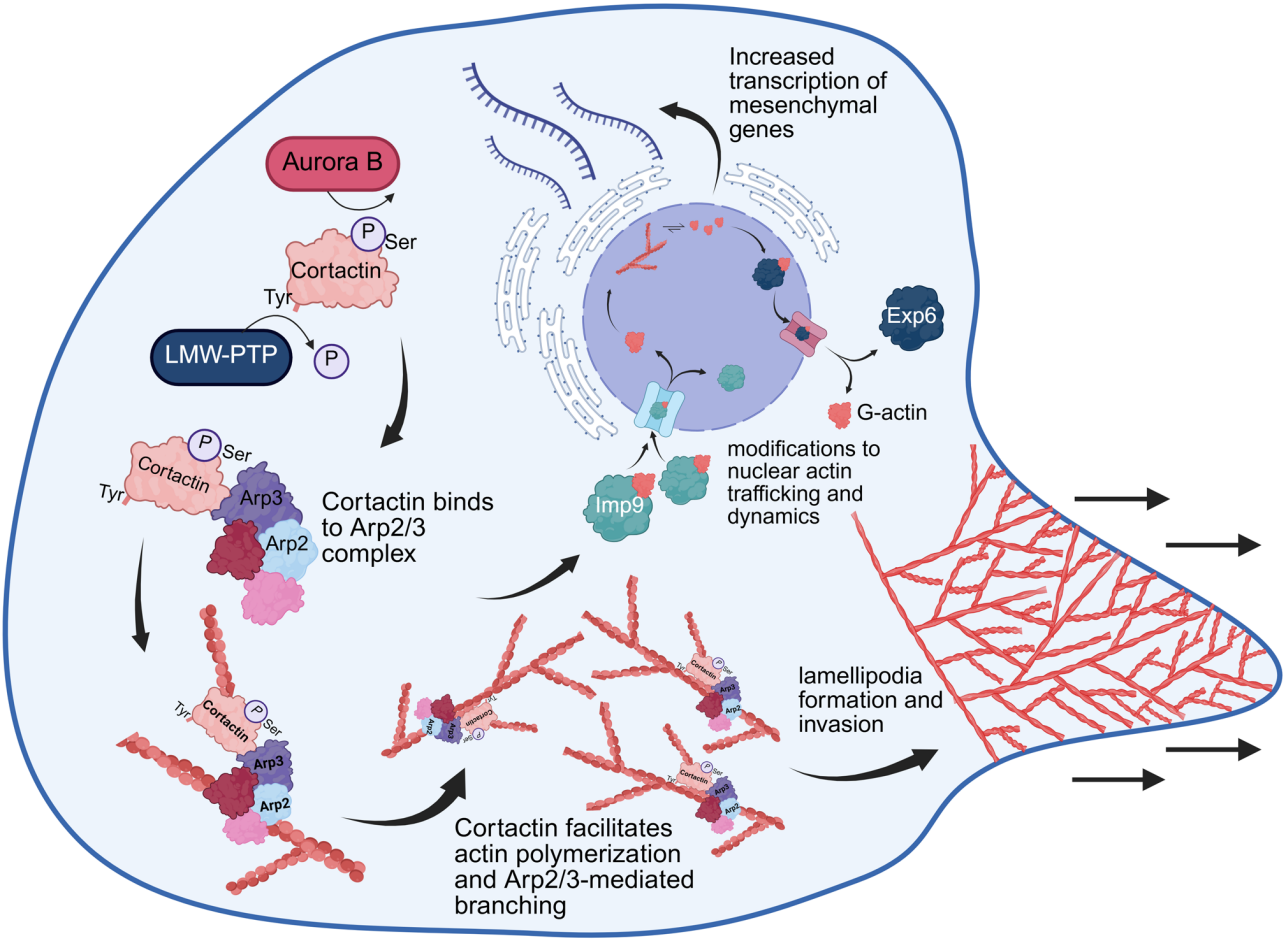
Summary of key findings. We found that aurora B–driven serine phosphorylation and LMW-PTP-driven tyrosine dephosphorylation of cortactin enhance cortactin binding of the Arp2/3 complex, facilitating actin polymerization and branching and altering nuclear actin distribution such that mesenchymal gene expression increases, promoting lamellipodia formation and invasion. Created in BioRender. Lovalvo I. (2026) https://BioRender.com/1nvm0bs

Although a defining contributor to GBM’s poor prognosis is invasion into surrounding white matter, progress in understanding targetable mediators of invasion remains limited. To close this gap, we developed a 3D hydrogel invasion platform for high-throughput screening of invasion mediators. The spatially dissectible nature of our hydrogel-based invasion devices allows us to analyze and perform CRISPR screens of GBM cells in the devices’ invasive front versus noninvasive core. Here, we used this approach, previously employed to define metabolic mediators of invading GBM cells (23), to identify druggable mediators of GBM invasion. We found that *AURKB* and *ACP1*, 2 hits from our druggable genome CRISPRi screen, converge on the serine phosphorylation and tyrosine dephosphorylation of cortactin, an actin-binding protein, caused by their gene products (Figure 8).

Aurora B, the protein encoded by *AURKB*, is a serine/threonine kinase whose best-established function is its role in the chromosomal passenger complex, a series of proteins regulating chromatin remodeling during cell division (40). Dysregulation leading to elevated aurora B activity correlates with cancer progression and poor prognosis. In GBM, targeting of aurora B can overcome temozolomide resistance (15), and a pan-AURK (A and B) targeting agent results in more favorable lipid metabolism and reduced invasion of GBM stem cells (41).

LMW-PTP, the protein encoded by *ACP1*, is implicated in signal transduction and actin cytoskeleton remodeling. Actin polymerization is critical to cancer cell invasion, enabling formation of lamellipodia and invadopodia, necessary for metastasis. In normal cells like osteoblasts, studies have demonstrated LMW-PTP’s role in modulating cell adhesion and migration via focal adhesion dynamics (42); in malignant cells like colorectal cancer cells, LMW-PTP is implicated in cell migration (16), critical for metastasis.

Expression of the actin-binding protein cortactin is higher in glioma specimens than nontumor brain tissue, with expression positively correlating with glioma grade, and targeting of cortactin has been shown to reduce the size and persistence of lamellipodia, migration, and invasion in 2D assays (25). Our work demonstrates the effect of targeting cortactin on GBM invasion in 3D assays and in vivo, providing insights into regulators of cortactin phosphorylation. Human cortactin can be phosphorylated on serines S-113, S-405, and S-418 and on tyrosines Y-421, Y-470, and Y-486 (24). We add to this finding by demonstrating that aurora B can phosphorylate serine(s) in cortactin, potentially S-81 and S-109 from database analysis, and that LMW-PTP can dephosphorylate cortactin tyrosine(s).

Our findings imply that serine phosphorylation and tyrosine dephosphorylation of cortactin promote GBM invasion and cytoskeletal remodeling. These potentially different effects of serine versus tyrosine phosphorylation on cortactin biology are similar to synaptopodin, another actin-binding protein affected differently by serine versus tyrosine phosphorylation. The latter promotes its interaction with calcineurin, facilitating synaptopodin degradation and cell morphology changes reducing invasion. Conversely, synaptopodin is stabilized via serine/threonine phosphorylation, facilitating formation of pro-invasive complex stress fiber networks and lamellipodia (43). For cortactin, the different effects of serine versus tyrosine phosphorylation are less well understood, limited to one cell biology study in which serine-phosphorylated cortactin promoted dendritic actin network assembly, while tyrosine phosphorylation of cortactin appeared to regulate focal adhesion dynamics (44). Further work is needed to determine the precise cellular effects of serine versus tyrosine cortactin phosphorylation and whether their differences can explain our findings, which imply that serine phosphorylation and tyrosine dephosphorylation of cortactin drives GBM invasion through effects on cortactin stability, leading to changes in actin remodeling.

Further work is also needed to explain differences we noted when targeting *CTTN* itself versus targeting *AURKB* or *ACP1*, like the reduced 3D cell volume observed when targeting *AURKB* or *ACP1* but not *CTTN*. A gene knockdown decreasing cell size implicates the targeted gene as a positive regulator of cell growth, proliferation, or structural integrity. Lack of proliferative differences in all 3 knockdowns (*AURKB*, *ACP1*, *CTTN*) argues against the second mechanism, meaning that our data implicates aurora B and LMW-PTP, but not cortactin, as positive regulators of cell growth or structural integrity, suggesting that this regulation is either (a) independent of the effects of aurora B and LMW-PTP on cortactin phosphorylation or (b) dependent on effects of these proteins on cortactin phosphorylation, with total cortactin loss facilitating a compensatory effect offsetting negative effects on cell growth or structural integrity seen when cortactin levels are partially reduced due to altered phosphorylation caused by *AURKB* or *ACP1* loss.

Our finding that only 2 of 8 genes from our CRISPR screen exhibited anti-invasive effects in a functional assay likely reflects our use of different complementary methods for the screen versus validation (large devices containing several million cells versus tumor spheroids containing a few thousand cells, respectively). This is further supported by the high specificity of CRISPR screens, as shown by false-positive rates below 5% when validating transcriptional effects of candidates using individual sgRNAs and qPCR (45), with the dCas9/KRAB/MeCP2 transcriptional repressor we used expected to show even more specificity (46). We used 3D hydrogel devices because of their capacity for the larger number of cells (several million) needed for the screen, the ability to dissect invasive versus noninvasive cells, and the ability of these devices to be used over a longer time (28 days). For validation, we used 3D spheroid invasion assays, enabling shorter-term studies with fewer cells in hydrogel material. Using these complementary methods added stringency to our screen results and increased our confidence as we moved to mechanistic studies based on results.

Beyond being a reasonable way to narrow large pools of targets and facilitate clinical translation, druggable genome screens have the added benefit of identifying mechanisms of biological processes. By performing a druggable genome screen to identify inhibitors of GBM invasion in our hydrogels, we identified aurora B and LMW-PTP as key druggable regulators of GBM invasion that activate a shared downstream target, cortactin, whose promotion of actin assembly was implicated in its invasion-promoting effects.

Although the invasion-promoting effects of *AURKB* were preserved in vivo given fractal analysis of tumor borders and suggested in vivo for *ACP1* via observed volumetric tumor contraction, targeting *AURKB* or *ACP1* did not affect survival in a murine model, underscoring the challenges of translating strategies that target GBM invasion for survival benefits. Further work is needed to determine whether lack of survival benefits reflects murine model limitations or whether anti-invasive therapies must be combined with cytotoxic chemotherapies; the former is supported by autopsy findings revealing that invasion exerts far less of an adverse effect on survival in mouse models than in humans (47). Furthermore, a potential need to combine anti-invasive approaches with cytotoxic chemotherapy is suggested by our finding of increased tumor cell density in smaller tumors when targeting *ACP1*, suggesting contraction without cell death.

## Methods

### Sex as a biological variable

We examined female mice. The relevancy of findings for male mice is unknown.

### Cell culture

GBM43, GBM102, and GBM28 PDX cells originated from Mayo Clinic, and U87MG cells originated from ATCC. PDX lines were cultured in DMEM (Thermo Fisher Scientific) supplemented with 10% (vol/vol) FBS (Corning, MT 35-010-CV), 1% (vol/vol) penicillin-streptomycin (Thermo Fisher Scientific), and 1% (vol/vol) GlutaMAX (Thermo Fisher Scientific, 35-050-061). U87MG cells were cultured in DMEM (Thermo Fisher Scientific) supplemented with 10% (vol/vol) FBS (Corning, MT 35-010-CV), 1% (vol/vol) penicillin-streptomycin (Thermo Fisher Scientific), 1% (vol/vol) sodium pyruvate, and 1% (vol/vol) MEM nonessential amino acids (Thermo Fisher Scientific). Cells were harvested using 0.25% trypsin-EDTA (Thermo Fisher Scientific), passaged under 30 times, screened bimonthly for mycoplasma, and validated every 6 months by short tandem repeat analysis at UC Berkeley or UCSF Cell Culture Facilities. For drug-treatment studies, cells were cultured in varying concentrations of AZD1152-HQPA or LMW-PTP inhibitor I (MLS-0322825 Cmpd23).

### 3D hydrogels

#### Methacrylate–hyaluronic acid synthesis.

Hyaluronic acid hydrogels were synthesized as described (37, 48). Methacrylic anhydride (Sigma-Aldrich, 94%) was used to functionalize sodium hyaluronate (Lifecore Biomedical, Research Grade, LMW: 66–99 kDa, HMW: 1.5 MDa) with methacrylate groups. Methacrylation extent per disaccharide was quantified by ^1^H NMR at approximately 90%–100% for materials. To add integrin-adhesive functionality, methacrylate–hyaluronic acid was conjugated via Michael addition with cysteine-containing RGD peptide Ac-GCGYGRGDSPG-NH2 (Anaspec) at 0.5 mmol/L.

#### Hyaluronic acid hydrogel rheological characterization.

Hydrogel stiffness was characterized by shear rheology via Physica MCR 301 rheometer (Anton Paar) with 8 mm parallel plate geometry for γ = 0.5% and f = 1 Hz. For LMW gels, frequency was controlled from 50 down to 1 Hz for frequency sweep at constant strain (γ = 0.5%); modulus saturation curve with time was obtained under oscillation with constant strain (γ = 0.5%) and frequency (f = 1 Hz). Gel solution temperature was controlled (T = 37°C) with a Peltier element (Anton Paar); the sample remained humidified. Storage modulus was determined as the value at which the curve plateaued; thiol to monomer ratio was that of di-thiol peptide crosslinker to hyaluronic acid monomer for each respective stiffness. HMW gels were incubated in a humidified chamber at 37°C for 2 hours. Hydrogels were trimmed to fit the 8 mm probe; a frequency sweep was performed to determine stiffness, read as the value at 1.256 rad/s (37), and a stress relaxing test (at 15% strain for 5 minutes, measuring stress every 0.5 seconds) determined normalized stress relaxation modulus.

#### Tumorsphere invasion assays.

Tumorspheres were fabricated using Aggrewell Microwell Plates (Stemcell Technologies). First, 1.2 × 10^5^ cells were seeded into each well, forming spheroids with 100 cells. After 48 hours, spheroids were resuspended in phenol red–free, serum-free DMEM (Thermo Fisher Scientific, 21-063-029) at 1.5 spheroids/μL and used as solvent for hyaluronic acid hydrogel crosslinking. To form hydrogels, methacrylate–hyaluronic acid (6 wt.%) was crosslinked in phenol red–free, serum-free DMEM (Thermo Fisher Scientific, 21-063-029) with a protease-cleavable peptide (KKCG-GPQGIWGQ-GCKK, Genscript). Hyaluronic acid–RGD gels were crosslinked with peptide crosslinkers at varying ratios to yield hydrogels with a shear modulus of approximately 300 Pa and a final methacrylate–hyaluronic acid concentration of 1.5 wt (Supplemental Figure 1B). After 1 hour crosslinking in a humidified 37°C chamber, media was added to hydrogels and replenished every 2 days. To analyze spheroid invasion assays, spheroids were imaged every 2 days using an Eclipse TE2000 Nikon microscope with a Plan Fluor Ph1 ×10 objective. Images were acquired using NIS-Elements software. Invasion was calculated as (A_f_–A_i_) / A_i_) where A_f_ = final spheroid area and A_i_ = initial spheroid area. Area was measured in ImageJ (NIH), and invasion was normalized to the invasion area of control spheroids.

### Invasion devices

#### Large devices.

The base, lid, and spacers were laser-cut from 1.5 mm thick CLAREX acrylic glass (Astra Products). Pieces were assembled and fastened with epoxy, UV-treated for 10 minutes, and stored in a cold room. On the day of the experiment, devices were brought to room temperature and a 22G × 1.5” bevel needle (BD Precision Glide) was inserted into the device as a channel mold. The hyaluronic acid hydrogel solution was cast around the wire and incubated for 1 hour in a humidified 37°C chamber. After crosslinking, devices were submerged in culture media for at least 10 minutes before removing the needle, leaving an open channel. Afterward, 4 million cells were seeded into the open channel, and ends plugged with vacuum grease. Devices were cultured for 28 days and media replenished every 3 days; experiments revealed 28 days as a timeline for full hydrogel invasion of GBM43 cells. Small devices were manufactured as described (49). The device base and lid were laser cut from 1.5 mm thick acrylic. Devices were assembled, and needle threading wires provided channel structure (Thomas Scientific). Polydimethylsiloxane (PDMS) (Ellsworth Adhesive, 184 SIL ELAST kit) was poured and cooled: channels were cut into PDMS and the laser cut top glued. Before seeding, small devices were treated with UV light for 20–30 minutes.

### CRISPRi knockdown screen

Lenti-X 293T cells were transfected using a lentiviral plasmid containing a dCAS9/KRAB/MeCP2 cassette (Addgene), from which virus was generated and titers determined. GBM43 cells were transduced with the virus and selected using 5 mg/mL blasticidin to obtain a pure dCAS9/KRAB/MeCP2-positive population. To validate dCAS9/KRAB/MeCP2 integration, positive GBM43 cells were transduced with a plasmid containing an sgRNA against *ITGB1*. Cells were harvested after 48 hours; knockdown efficiency was measured using Western blot.

Druggable genome sgRNA libraries were designed and screening was performed as described (50). The druggable gRNA library consisted of 13,250 sgRNAs against 2,550 genes (5 sgRNAs/gene) and 250 sgRNAs against 50 genes as negative controls. sgRNA oligonucleotides were synthesized by Integrated DNA Technologies, amplified by PCR, and cloned into pLG1 library vector containing red fluorescent protein (RFP) for selection. Lenti-X 293T cells were transfected using this cloned gRNA library plasmid. Virus was generated and titered. The sgRNA library was introduced into GBM cells as a lentiviral pool. GBM43 cells expressing the CRISPRi machinery (dCAS9/KRAB/MeCP2) were infected with the virus and selected for RFP using flow cytometry. So that only 1 sgRNA was introduced per cell, infection was optimized to 30%–40% transduction efficiency.

To study invasive differences, we incorporated 25 million GBM cells expressing this library into hydrogels. The top and bottom 10% of most and least invasive cells, measured by invasion distance through the 3D matrix, were visualized using fluorescent imaging for RFP. Invasive cells were defined as invading over 200 μm from the channel wall. Genomic DNA (gDNA) was extracted from harvested cells using Monarch Genomic DNA Purification kit (New England BioLabs, T3010S) per the manufacturer’s protocol. gDNAs from 6 devices were pooled and amplified by PCR and sequenced using Illumina HiSeq 4000. Sequenced reads were aligned to CRISPR library gRNAs and quantified using a previously described Screen Processing pipeline (https://github.com/mhorlbeck/ScreenProcessing) (51). Negative controls included nonfunctional and nontargeting gRNAs. We used the reference CRISPRi_v1_human.trim_1_35.fa with trim settings of –trim_start 1 –trim_end 35. Subsequent analysis assigned read counts generated for each sequencing file to the appropriate sample (core versus invasive) to generate phenotypes. Filtering and normalization techniques were otherwise the default in the template file: either or equal to 50 minimum reads; add 1 pseudo-count to 0 number values; combined sgRNAs by transcripts; auto-matching of pseudogenes to targeting library table; pseudogene size 10; 16,000 pseudogenes; average calculations from best 3 sgRNAs; calculate Mann-Whitney *P* values set to true; calculate Kolmorgorov-Smirnov *P* values set to false; and separate scoring based on *n* best sgRNAs set to false. Sequencing counts from samples were summed, normalized (count/million), and analyzed as single conditions. Fitness scores for each guide were calculated as the log_2_ ratio of normalized counts. The median of the guides was used as the fitness score for each gene, and a 2-tailed *t* test was used to assess whether guides significantly deviated from 0.

To validate positive screening hits using single-gene CRISPRi, a lentiviral plasmid containing a dCAS9/KRAB/MeCP2 cassette was obtained from Addgene (122205). Lenti-X 293T cells were transfected, virus generated, and appropriate titers determined. GBM43 cells were transduced and selected using 5 μg/mL blasticidin to obtain pure dCAS9/KRAB/MeCP2^+^ cells. GBM43 cells expressing dCAS9/KRAB/MeCP2 were transfected with the plasmid containing sgRNAs (Supplemental Table 4) and a puromycin resistance gene, then selected using 5 μg/mL blasticidin and 5 μg/mL puromycin. Gene knockdown was confirmed using qPCR and Western blot.

### TurboID and proteomics

Vector C1(1-29)-TurboID-V5_pLX304 (Addgene, 107175), containing aa 1–29 of cytochrome P450 fused to the TurboID sequences, was the control for TurboID assays. For experimental groups, coding sequences for *ACP1* and *AURKB* were PCR-amplified from expression plasmids obtained from Origene (*ACP1*: RC212653; *AURKB*: RC210288) and cloned into this vector using Gibson Assembly (NEB Gibson Assembly Master Mix, E2611S) Cells lentivirally transduced with these TurboID vectors underwent 10 μg/mL blasticidin selection. Before collection, GBM43 cells were treated with 10 μM biotin and incubated at 37°C for 10 minutes. Protein lysates were prepared using RIPA buffer. After calibrating concentrations, streptavidin magnetic beads (Thermo Fisher Scientific, 88816) were added to lysates and incubated at room temperature for 1 hour, permitting bead-protein conjugation without unspecific binding. After washes (RIPA, KCL, Na_2_CO_3_, and urea) (52), proteins eluted from beads were collected for Western blot. Protein lysates were verified using antibodies against V5 protein tag and biotin, and streptavidin beads were sent for MS as per the Supplemental Methods.

### Immunofluorescence of cultured cells

GBM43 cells were cultured on coverslips coated with 1 μg/mL fibronectin in PBS for 4 hours and placed in 6-well plates. At 50% confluency, they were fixed in 3.7% formaldehyde and 0.1% Triton X-100 in PBS for 10 minutes, then stained with antibodies. Primary antibodies (Supplemental Table 5) for LMW-PTP (R&D Systems, MAB5075-SP), aurora B (EnCor Biotechnology, MCA-6G2), cortactin (Proteintech, 11381-1-AP), phospho-serine (Santa Cruz Biotechnology, sc-81514), phospho-threonine (Santa Cruz Biotechnology, sc-5267) and p-tyrosine (Santa Cruz Biotechnology, sc-7020) were used to characterize distribution and quantity. DAPI was used to stain nuclei; phalloidin (594 nm) stained the actin cytoskeleton.

### Sandwich ELISA to detect phosphorylated cortactin

Anti-cortactin rabbit polyclonal (Proteintech, 11381-1-AP) diluted in PBS at 2 μg/mL was left overnight in a high-attachment, 96-well plate. Subsequently, the plate was blocked for 1 hour at room temperature (5% BSA, 0.05% Tween-20). Next, 200 ng of Cortactin (Origene, SKU TP710315) was combined with 200 ng aurora B (Abcam, AB51435-10UG) or 220 ng c-Src (Sino Biologicals, 10755-H20B) and suspended in kinase buffer (Cell Signaling Technology, 98025, 25 mM Tris-HCl pH 7.5, 5 mM beta-glycerophosphate, 2 mM dithiothreitol, 0.1 mM Na_3_VO_4_, 10 mM MgCl_2_) to 150 μL. ATP was added to a concentration of 100 μM in tubes incubated at 30°C and 500 rpm in a heated shaker for 40 minutes. After some c-Src kinase reactions, samples were centrifuged (18,000*g*, 4 rounds, 10 minutes each) in 10 kDa spin columns (Abcam, AB93349) with PBS to remove kinase buffer, then resuspended in 150 μL acid phosphatase buffer (50 mM sodium acetate (Invitrogen AM9740) with250 ng LMW-PTP (Sino Biologicals, 10957-H09E). Tubes were incubated in a heated shaker at 37°C and 500 rpm for 45 minutes. Samples were incubated on ELISA plates for 1 hour. After washing, 100 μL of detection antibody (Thermo Fisher Scientific, MA191608, or Santa Cruz Biotechnology, SC7020) at 2.2 μg/mL was added and incubated 1 hour. Plates were washed 5 times. Next,100 μL of secondary goat anti-mouse HRP Affinity (Invitrogen, A16066) was added at 0.2 μg/mL and incubated for 1 hour. The plate was washed 5 times. Afterward, TMB substrate (Abcam, AB171523) was added at 100 μL. The reaction was stopped with 50 μL of 1M H_2_SO_4_. A plate reader measured OD at 450 nm and background was subtracted.

### Actin polymerization assay

Cell lysates were prepared in a non-denaturing form using IP lysis buffer (Thermo Fisher Scientific, 87787) and spun in 10 kDa purification columns (Abcam, ab93349) at 9,391*g* for at least 30 minutes. Concentrations were measured with a BCA assay to equalize input (100 mg protein in Figure 5, A–C, 50 mg protein in Supplemental Figure 5A). Records occurred in 96-well optical-bottom plates with solid polystyrene black upper structures and scanned using a BioTek synergy neo2 hybrid multimode reader. Fluorometer and kinetic measurement programs were set according to manual. Fluorescent signals over 1-hour were normalized to initial signals; linear regression generated slopes of the time courses.

### Murine intracranial xenograft tumors

Animal experiments were approved by UCSF IACUC (approval AN105170-02). First, 20,000 cells (GBM43/sgGAL4, GBM43/sgAURKB, GBM43/sgACP1, or GBM43/sgCTTN) at 10,000 cells per milliliter were implanted intracranially into right frontal lobes of athymic mice (6–8 weeks, female; The Jackson Laboratory, strain 002019) stereotactically. Tumors were explanted when mice met institutional euthanasia criteria.

### Tissue processing and sectioning

After reaching institutional euthanasia criteria, cardiac perfusion occurred with PBS. Whole brains were put on ice, transferred to 4% PFA for 24 hours, placed into 15% (weight/vol) sucrose in PBS until infiltration occurred, then transferred to 30% sucrose (weight/vol). Afterward, brains were embedded in OCT (Sakura Tissue Tech), snap-frozen using dry ice and 100% ethanol, then sectioned via cryostat at 10 μm thickness, and transferred onto glass slides stored at –80°C.

### Tumor immunostaining

Sectioning slides were thawed, fixed with 4% PFA for 10 minutes, blocked, and permeabilized with 2.5% FBS (vol/vol), 2.5% BSA (weight/vol), 0.1% Triton-X-100 (vol/vol) in PBS for 1 hour. Cortactin antibody (Proteintech 11381-1-AP; Supplemental Table 4) was diluted 1:1,000 to 650 ng/mL in blocking solution and incubated overnight at 4°C. Slides were then stained with secondary antibody, Invitrogen goat anti-rabbit IgG (H+L) cross-adsorbed secondary antibody, and Alexa Fluor 488 with phalloidin (Abcam Phalloidin iFluor 594), both diluted 1:1,000 in blocking solution, for 2 hours in the dark at room temperature. Afterward, nuclear counterstaining occurred using NucRed Dead 647 (Thermo Fisher Scientific) at 2 drops/mL in PBS, for 20 minutes at room temperature. Slides were washed, air dried, mounted to coverslips with glycerol, and stored in the dark at 4°C. Images were taken via the BC43 spinning disk confocal microscope at ×60 original magnification. The green, red, and far-red channels imaged cortactin, actin, and nuclear counterstain. ImageJ (NIH) was used to quantify IHC analysis.

### Image analysis

Cytoplasmic and nuclear actin distribution was analyzed using a previously described Fiji Macro (53) using images of cells at identical exposure and laser power at 60× magnification. On ImageJ (NIH), they were identically thresholded and converted to binary format, where remaining filled space was quantified. For colocalization, multichannel images at 60× magnification were split and converted to produce red or green signals. Two channels were combined to produce yellow signal indicating overlap, then color-thresholded to only yellow areas converted to binary format and quantified.

### Statistics

Assays had at least 3 technical and biological replicates. To compare multiple groups, 1-way ANOVA (parametric) or Kruskal-Wallis (nonparametric) tests were used for continuous outcome variables, with χ^2^ and Fisher’s exact tests for categorical outcome variables. One-way ANOVA, unless otherwise specified, or Kruskal-Wallis tests were followed by Tukey’s or pairwise Wilcoxon’s post hoc tests for comparisons between groups, respectively. Parametric 2-tailed *t* tests compared 2 groups. Kaplan-Meier analysis occurred for in vivo survival studies. *P* values of less than 0.05 were considered significant.

### Study approval

Animal experiments were approved by UCSF IACUC (approval AN105170-02).

### Data availability

MS proteomic data have been deposited to the ProteomeXchange Consortium and made publicly available via the PRIDE (54) partner repository with the dataset identifier PXD077507. Values for all data points in graphs are reported in the Supporting Data Values file.

## Author contributions

MH, AW, IML, EAA, and SJ designed the project, conducted experiments, processed data, interpreted results, and edited the manuscript. AC, AS, ML, AL, AR, AK, and EAD conducted experiments, processed data, and interpreted results. JAOP, PE, and ALB performed MS experiments and analysis. SK and MKA procured funding, designed experiments, interpreted results, and wrote and edited the manuscript.

## Conflict of interest

The authors have declared that no conflict of interest exists.

## Funding support

This work is the result of NIH funding, in whole or in part, and is subject to the NIH Public Access Policy. Through acceptance of this federal funding, the NIH has been given a right to make the work publicly available in PubMed Central.

- National Science Foundation Graduate Research Fellowship and the NIH T32 Biotechnology of Cell and Gene Therapy Predoctoral Fellowship (to AW).
- NIH grants S10OD020054, S10OD021741, and S10OD026881 (supporting the UCSF CryoEM Core and David Bulkley).
- National Science Foundation Graduate Research Fellowship (to EAA).
- NIH grants R01CA227136, R01CA260443, and R01GM122375 (to SK) and R01CA227136, R01NS079697, and R01CA260443 (to MKA).
- UCSF Laboratory for Cell Analysis (grant P30CA082103) supported fluorescence microscopy/flow cytometry studies.
- Howard Hughes Medical Institute (supporting the UCSF CryoEM Core and David Bulkley).
- Dr. Miriam and Sheldon G. Adelson Medical Research Foundation (to ALB, JAOP, and PE).

## Supplementary Material

Supplemental data

Unedited blot and gel images

Supplemental table 1

Supplemental table 2

Supplemental table 3

Supplemental table 4

Supplemental table 5

Supplemental table 6

Supplemental video 1

Supporting data values

## Version 1 08/10/2026

Electronic publication
