# Supplementary material for: Druggable genome CRISPRi screen in hydrogels reveals regulators of cortactin-driven actin remodeling promoting glioblastoma invasion
Source: JCI Insight. 2026 Aug 10;11(15):e196484. doi: 10.1172/jci.insight.196484 (PMC13463607; doi:10.1172/jci.insight.196484)
Supplement: Supplementary file 1 [file jciinsight-11-196484-s138.pdf]

SUPPLEMENTAL METHODS

Mass Spectometry

In preparation for digestion, sample-incubated streptavidin magnetic beads were resuspended in 9.4 µL of 5 mM Tris(2-carboxyethyl)phosphine (TCEP) 20 mM triethylammonium bicarbonate (TEAB) pH 8.5, and incubated for 30 min at 56°C. Iodoacetamide was added to 7.5 mM and samples incubated for 30 minutes at room temperature. 1 µg of MS-grade trypsin (Thermo Scientific) was added to each sample and incubated at 37°C overnight. Supernatants were recovered in clean 0.6 mL low protein binding vials, and 10 µl of TEAB containing 1 µg of trypsin was added to each sample and incubated at 37°C for 4 hours. Supernatants of the beads were recovered into the same set of low protein binding tubes as before, and acidified with 5% formic acid. Peptides recovered by solid phase extraction using C18 ZipTips (Millipore) were resuspended in 0.1% formic acid for analysis by LC-MS/MS. Peptide digests were subjected to chromatography using a 15-cm EasySpray C18 column (Thermo Scientific). 60 min water-acetonitrile gradients (2–25% in 0.1% formic acid) were used to separate peptides, at 200 nL/min, for analysis in a Orbitrap Exploris 480 (Thermo Scientific) in positive ion mode. MS spectra were acquired between 375 and 1400 m/z with resolution of 120000. For each MS spectrum, multiply charged ions over the selected threshold (2E4) were selected for MS/MS in cycles of 3 seconds with an isolation window of 1.6 m/z. Precursor ions with a charge state of 2+ or higher were fragmented by HCD using a relative collision energy of 30. MS/MS spectra were acquired in centroid mode with resolution 30000 from m/z=120. A dynamic exclusion window was applied which

prevented the same m/z from being selected for 30s after its acquisition. Peak lists generated using PAVA software (1) were searched against the human subset of the SwissProt database (SwissProt.2019.07.31) using Protein Prospector (2). The database search was performed with the following parameters: mass tolerance of 10 ppm for precursor masses; 30 ppm for MS/MS, cysteine carbamidomethylation as a fixed modification and acetylation of the N terminus of the protein, pyroglutamate formation from N terminal glutamine, and oxidation of methionine as variable modifications. A refined search was performed based on results of this first search, correcting the systematic error of the distribution (3.65 ppm) and allowing mass tolerance of 10 ppm for precursor masses All spectra identified as matches to peptides of a given protein were reported, and the number of spectra (Peptide Spectral Matches, PSMs) used for label-free quantitation of protein abundance in the samples. Peptide spectral matches were analyzed for differential expression by DESeq2 package in R.

Gene Ontology Enrichment

For Gene Ontology (GO) enrichment analysis, we leveraged the Molecular Signatures Database (MSigDB) to collect KEGG gene set libraries and identify significantly enriched items. Initially, we compiled a list of genes whose encoded proteins were effective hits ($\log_2 FC>1$) for both ACP1 and AURKB. We then mapped these genes to their respective terms using annotations from the KEGG database. The KEGG library facilitated the enrichment analysis by calculating the enrichment scores for each gene set based on the frequency of genes within the term compared to a background set. Statistical significance was determined using Fisher's exact test, and a

Benjamini-Hochberg correction was applied to control for false discovery rates, with a threshold set at FDR < 0.01. Correlation of gene sets was computed using shared content genes before hierarchical clustering.

Cytoscape

To visualize the networks of ACP1 and AURKB-affected targets that regulate key processes involved in cell invasion, we utilized Cytoscape, an open-source platform for complex network analysis and visualization. We imported the effective hits from TurboID proteomic data for ACP1 and AURKB, followed by performing gene ontology enrichment analysis on all genes. We then filtered the large global networks to focus on subsets of genes based on identified biological processes most relevant to cell invasion. *CTTN*, a hub gene among these processes, was highlighted, with nodes representing individual proteins and edges denoting their interactions. Visual attributes like node size were adjusted to reflect the enrichment of genes from TurboID proteomic data.

qPCR

RNA was generated from knockdown cells using Qiagen's RNeasy Plus Mini Kit (Cat. No 74134) and was converted to cDNA using qScript XLT cDNA Supermix (Quanta Bio), according to standard protocol. Quantitative PCR was performed using Power Syber Green Master Mix, primers (**Supplemental Table 6**) and an Applied Biosystems QuantStudio3 cycler using the following parameters: Holding stage – 95°C, 2 minutes. PCR Stage (40 cycles) - 95°C/15 seconds, followed by 63°C/ 30 seconds. For *FOXC1* primers only, PCR Stage parameters were 95°C/15 seconds, then 65°C/30 seconds.

Electron Microscopy

Two μL of actin polymerization assay sample at a concentration of 0.05 mg/mL was applied to glow-discharged 300 mesh copper formvar carbon grids and allowed to dry for 30 seconds. The grids were then washed in sterile ddH2O before being negatively stained with 2% uranyl acetate for 30 seconds and left to dry. Montage images were then taken on a *Tecnai T12* transmission electron microscope at 6500X magnification with 15% overlap and analyzed using ImageJ.

Immunoblots and Immunoprecipitation

GBM43 cells, including GBM43/sgACP1 and GBM43/sgAURKB cells, were lysed in RIPA buffer supplemented with 10% protease inhibitor cocktail (Roche #05892970001) and 10% phosphatase inhibitor cocktail (Roche #4906845001). Lysates were quantified using the Pierce BCA kit (Thermo Fisher), and 10-30 μg of total proteins were used per loading. Protein samples were subjected to SDS-PAGE, denatured at 95°C, electrotransferred to membranes, and immunoblotted. For immunoprecipitation, 1000-3000 μg of cell lysate proteins were incubated with specific primary antibodies at 2 μg/ml overnight at 4°C. The immunoprecipitated complexes were verified by western blotting using a Licor Odyssey system.

Adhesion Assay

Clear 96 well plates were coated with 100 μL of either 40 μg/mL Bovine Serum Albumin (Fraction V) (Fisher) or 10 ug/mL human fibronectin (Corning) in PBS and incubated overnight at 4°C. Cells were trypsinized, counted to 2.5 x 10^5 cells/ml, and serum starved in DMEM without phenol red (Fisher) for 2 hours at 37°C.The plate was concurrently blocked using 0.5% BSA (weight/vol) in DMEM without phenol red for 1 hour at 37°C, then chilled on ice. 2 x 10^4 cells in serum-free media solution were added to each well and the plate was incubated at 37°C for 90 minutes. Wells were then fixed with 100 μL of 4% paraformaldehyde (Fisher) for 15 minutes at room temperature, washed with PBS, then stained with Crystal Violet (Sigma Aldrich) for 10 minutes. The plate was washed and fully submerged in tap water, then left upside down to dry completely overnight. 50 μL of Sorenson's Phosphate Buffer was added to each well the following morning and left to incubate at room temperature for 30 minutes. The optical density of each well was read at 550 nm via a BioTek Epoch Microplate Reader.

Pharmacological Cell Viability

For 2D kill curves, cells were seeded at 20k cells/mL in a 48-well plate and allowed to adhere overnight. Wells were then treated with media and varying concentrations of the drug (in triplicate) and allowed to incubate for 48 hours. Cells were then fixed using 4 % PFA for 20 minutes and incubated with DAPI (1:1000) for 1 hour. Fluorescent images were taken on a Nikon Eclipse Ti-E epifluorescence microscope. Cells were counted using ImageJ, and total counts were normalized to the average number of cells in the vehicle condition to yield a percent viability value.

3D Live/Dead Assay

Cells were encapsulated following the spheroid protocol and allowed to invade for 3 days. Afterwards, cells were incubated with components from the Live/Dead Cell Imaging Kit (ThermoFisher, R37601) for 45 minutes. During the last ten minutes of incubation, Hoechst (ThermoFisher, H1399, 5 ug/mL) was added to visualize nuclei. Live, confocal fluorescence imaging was performed on a Zeiss LSM880 FCS confocal microscope. Using ImageJ, viability was quantified from the z-stack using the following formula: Viability (%) = $100*[(Area_{total} - Area_{dead})/ Area_{total}]$, where $Area_{total}=Area_{live}+Area_{dead}$.

Apoptosis Assay

Cells were plated at 100,000 per well in triplicate and left to grow for 42 hours. Cells were trypsinized and washed in stain buffer at 300 rcf for 5 minutes. Apotracker Green (Biolegend Cat No. 472402) was diluted 1:10 in stain buffer and added to washed cells to produce a 400 nM staining solution. Cells were left to incubate at room temperature for 15 minutes, then washed and stained in PBS with Zombie Red Fixable Viability Dye (Biolegend Cat No. 423109) for 20 minutes. Cells were washed twice before being run on the Attune Nxt Flow Cytometer and analyzed for viable and apoptotic populations using FlowJo v10.8.1.

Atomic Force Microscopy

Falcon Bacteriological Petri Dishes (Corning 351006) were briefly coated with Poly-D-Lysine hydrobromide (Millipore Sigma, 27964-99-4) dissolved in PBS (0.1 mg/mL) to assist with cell adhesion. Dishes were then washed with PBS for 3 minutes to remove residual poly-d-lysine. Cells were then plated at 100k cells per dish and allowed to adhere overnight. Cells were measured at 37°C under a HEPES-buffer medium with an MFP-3D-BIO AFM (Oxford Instruments) using pyramidal probes of nominal spring constant 0.08 N/m (PNP-TR-AU, Nanoworld) and a trigger threshold of 1 nN. Cantilevers were calibrated by the thermal method before using. Raw data was exported to MATLAB and Young's modulus for each measurement was extracted by fitting the contact region to a pyramidal Hertz model with a Poisson ratio of 0.5.

Tumor Fractal Analysis

Unstained slides containing cross sections from the widest area of the tumor were allowed to thaw at room temperature for 10 minutes. Images were taken using the BC43 spinning disk confocal benchtop microscope at 20X magnification of the tumor's entirety and the surrounding region of brain tissue, using the confocal blue channel to observe areas containing BFP, indicating tumor tissue. Images were then converted to binary and analyzed for fractal complexity based on Box Counting Dimension (D_B) using the automated FracLac plugin on ImageJ.

REFERENCES

1. Guan S, Price JC, Prusiner SB, Ghaemmaghami S, and Burlingame AL. A data processing pipeline for mammalian proteome dynamics studies using stable isotope metabolic labeling. *Mol Cell Proteomics.* 2011;10(12):M111 010728.
2. Clauser KR, Baker P, and Burlingame AL. Role of accurate mass measurement (+/- 10 ppm) in protein identification strategies employing MS or MS/MS and database searching. *Anal Chem.* 1999;71(14):2871-82.

Supplemental Figure 1. Overview of the multi-omic workflow used to identify targetable mediators of invasion in GBM.

A. Schematic for how the druggable genome CRISPRi screen was done in GBM43 cells invading 3D HA-RGD hydrogels.

B. Hydrogel storage modulus (G') (in Pascals), which represents energy stored during deformation, vs. time after the HA-RGD gel is cast. Time=0 represents the intersection of storage modulus (G') and loss modulus (energy dissipated during heat deformation=G'') (n=3 hydrogels). For our CRISPRi screen, HA-RGD gels were crosslinked with peptide crosslinkers at varying ratios to yield hydrogels with a shear modulus (a measure of rigidity) ~300 Pa and a final 1.5 wt.% Me-HA as shown on the left.

A

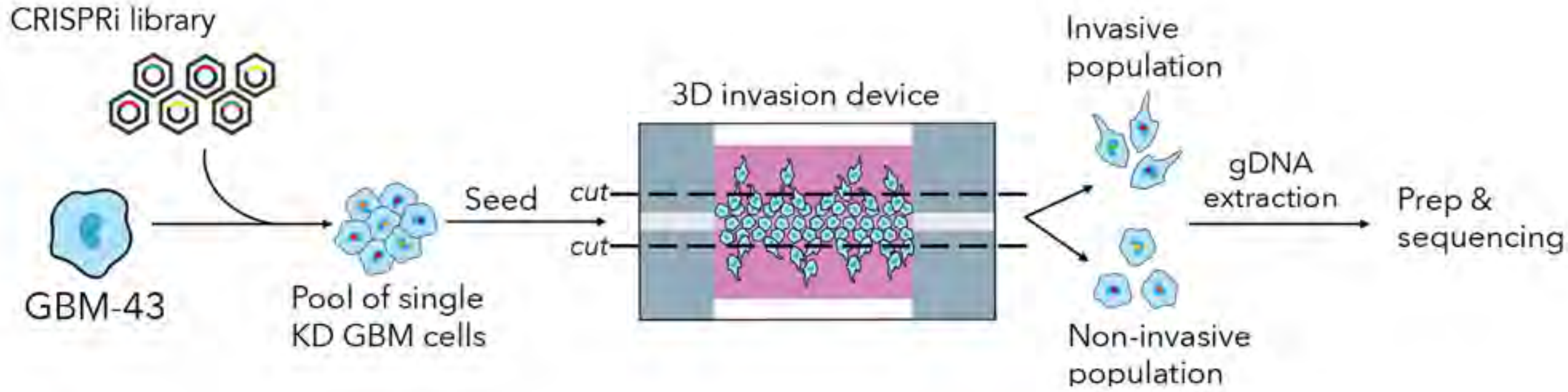

B

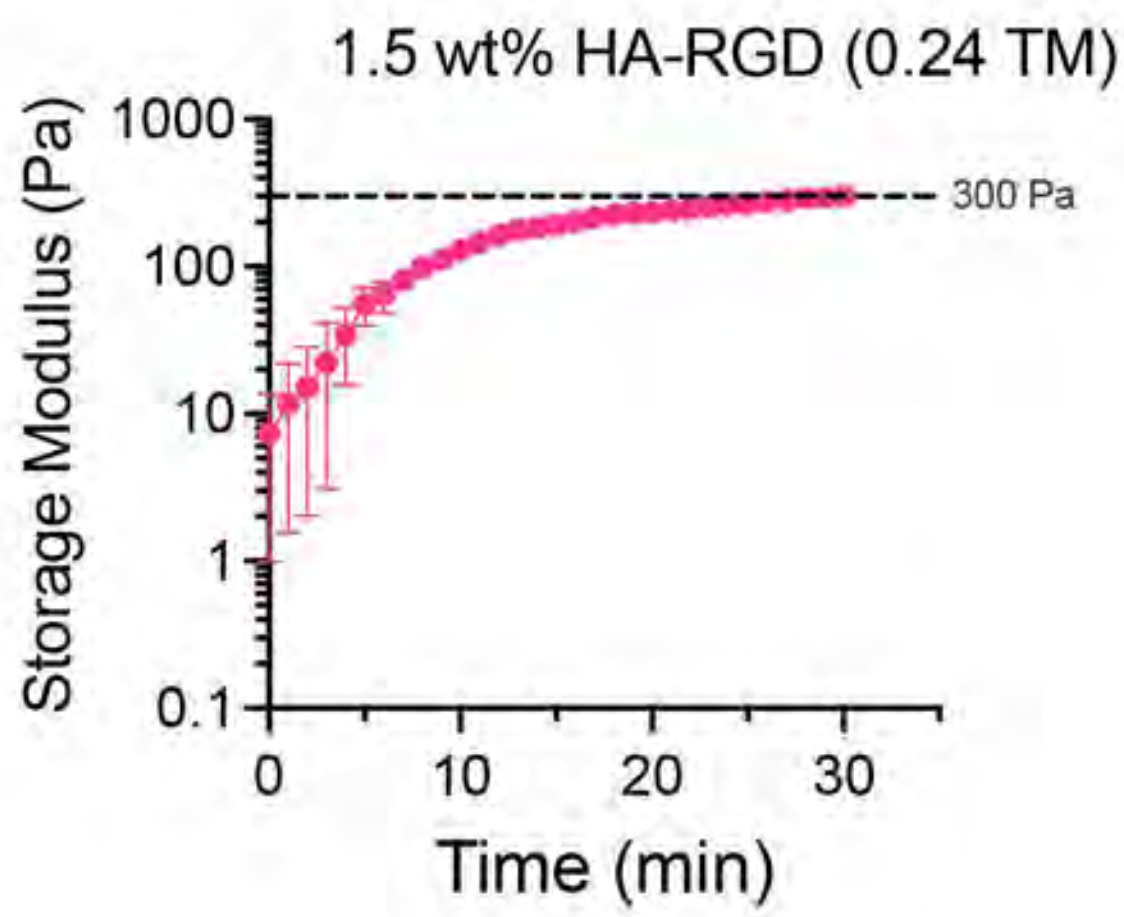

Supplemental Figure 1

Supplemental Figure 2. CRISPRi screen identifies druggable genes mediating GBM invasion in 3D hydrogels.

A. Results of qPCRs confirming degree of knockdown relative to GBM43/sgGAL4. All knockdowns had P<0.001 for expression of the gene being targeted relative to GBM43/sgGAL4 (n=3/group).

B. Proliferation, as assessed by DAPI staining after 48 hours in culture, was comparable between GBM43/sgGAL4, GBM43/sgAURKB, and GBM43/sgACP1 cells (P=0.5-0.9, n=3/group).

C. Cell viability for GBM43 cells exposed to increasing concentrations of AURKB inhibitor AZD1152-HQPA or ACP-1 Inhibitor LMW-PTP Inhibitor I.

D. Results of GBM43 spheroid invasion assay done in the presence of AURKB inhibitor AZD1152-HQPA, ACP1 inhibitor LMW-PTP Inhibitor I, or both inhibitors combined for 4 days (n=54-67 spheres per condition across 3 biological replicates). Both inhibitors combined disrupted invasion than either inhibitor alone (P<0.0001). Scale bar: 100 μm.

E. *Left*: Cell viability for GBM102 cells exposed to increasing concentrations of AURKB inhibitor AZD1152-HQPA and ACP1 inhibitor LMW-PTP Inhibitor I. *Right*: Results of neurosphere invasion assay for GBM102 cells grown in the presence of the highest nontoxic concentration of AURKB inhibitor AZD1152-HQPA (P<0.0001) or ACP1 inhibitor LMW-PTP Inhibitor I (P=0.02) for 9 days (n=47-61 spheres across 3 separate biological replicates). Scale bar: 100 μm.

F. *Left*: Cell viability for GBM28 cells exposed to increasing concentrations of AURKB inhibitor AZD1152-HQPA or ACP1 inhibitor LMW-PTP Inhibitor I. *Right*: Results of

neurosphere invasion assay for GBM28 cells grown in vehicle vs. the presence of the highest nontoxic concentration of AURKB inhibitor AZD1152-HQPA (P=0.0004) or ACP1 inhibitor LMW-PTP Inhibitor I (P=0.02) for 11 days (n=32-49 spheres across 3 separate biological replicates). Scale bar: 100 μm.

ns=not significant, *P<0.05; **P<0.01; ***P<0.001; ****P<0.0001. One-way ANOVA with Tukey pairwise post-hoc comparisons. Dose-response curves and EC50 values were derived from nonlinear regression and the EC50 equation in Prism.

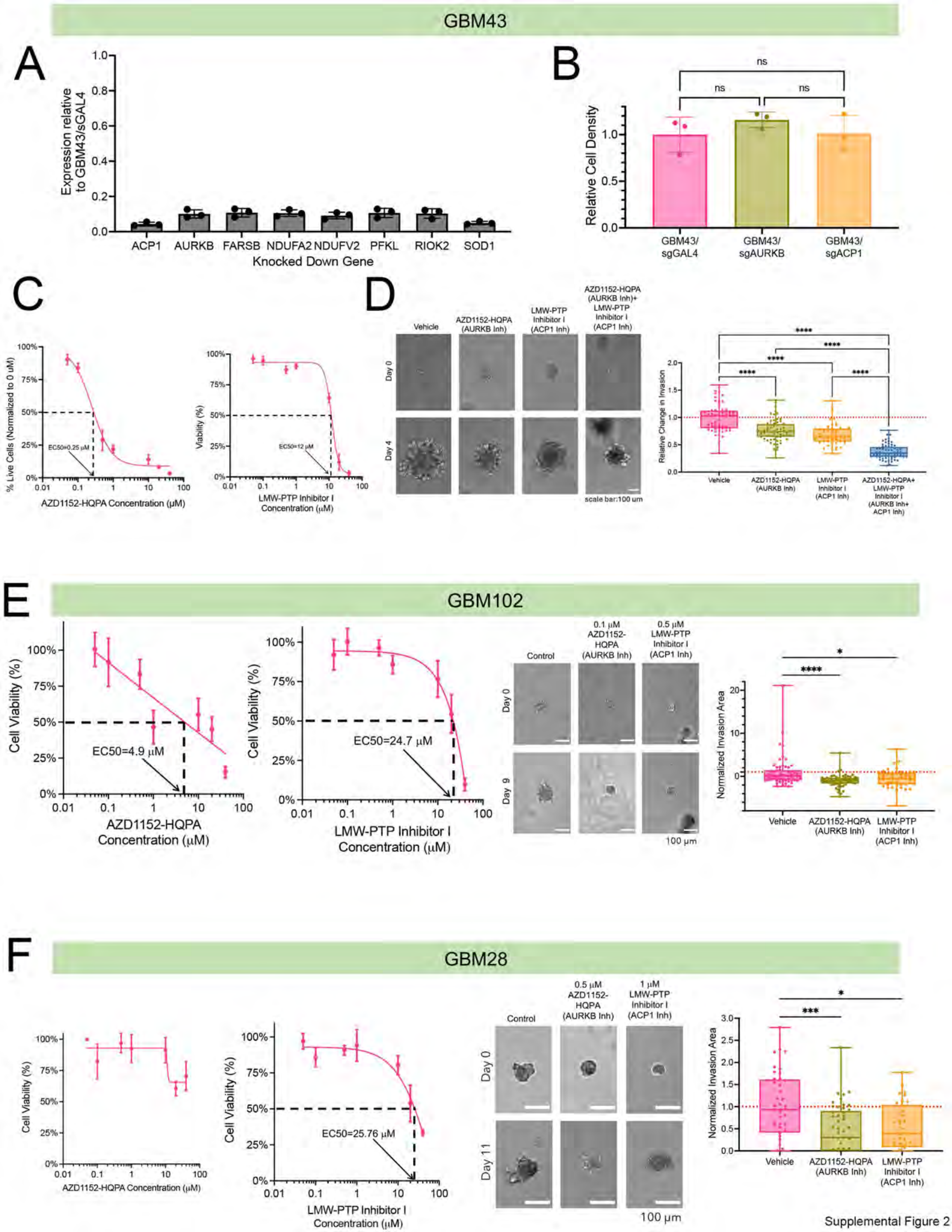

Supplemental Figure 2

Supplemental Figure 3. Proximity labeling identifies cortactin as a binding partner for AURKB and ACP1 with a role in GBM invasion and whose expression increases in invasive GBM cells.

A. Venn diagram showing that, of 2,814 different proteins identified by MS, 903 and 1,437 bound to Turbo-ID Aurora B and LMW-PTP fusion proteins, respectively, with greater than 2-fold increase compared to the control Turbo-ID cytochrome P450 fusion protein, with 703 proteins shared between the two.

B. The top 10 most enriched proteins binding both Turbo-ID Aurora B and LMW-PTP fusion proteins compared to control Turbo-ID cytochrome P450 fusion protein. Error bars represent standard errors of the mean.

C. KEGG pathways shared amongst binding partners of both Aurora B and LMW-PTP plotted based on fold enrichment (x-axis) and $-\log_{10}$(FDR) (y-axis) with pathways related to invasion highlighted. Three pathways concerning cancer invasion are highlighted along with correlation between their gene sets: proteoglycans in cancer, regulation of the actin cytoskeleton, and focal adhesion.

D. Strength of cortactin binding to Turbo-ID Aurora B and LMW-PTP fusion proteins compared to control Turbo-ID cytochrome P450 fusion protein.

E. Results of qPCR confirming knockdown of *CTTN* in GBM43/sgCTTN relative to GBM43/sgGAL4 (n=3 replicates). P=0.007 for *CTTN* expression in GBM43/sgCTTN compared to GBM43/sgGAL4 cells.

F. Neurosphere invasion assay results in GBM43/sgCTTN relative to GBM43/sgGAL4. Scale bar: 100 μm; n=38-50 spheres/condition; P<0.0001 by t-test.

G. Cell viability, as assessed by an MTS assay after 48 hours in culture, was comparable between GBM43/sgGAL4 and GBM43/sgCTTN cells (P=0.4; t-test; n=10/group).

ns=not significant, *P<0.05; **P<0.01; ***P<0.001; ****P<0.0001

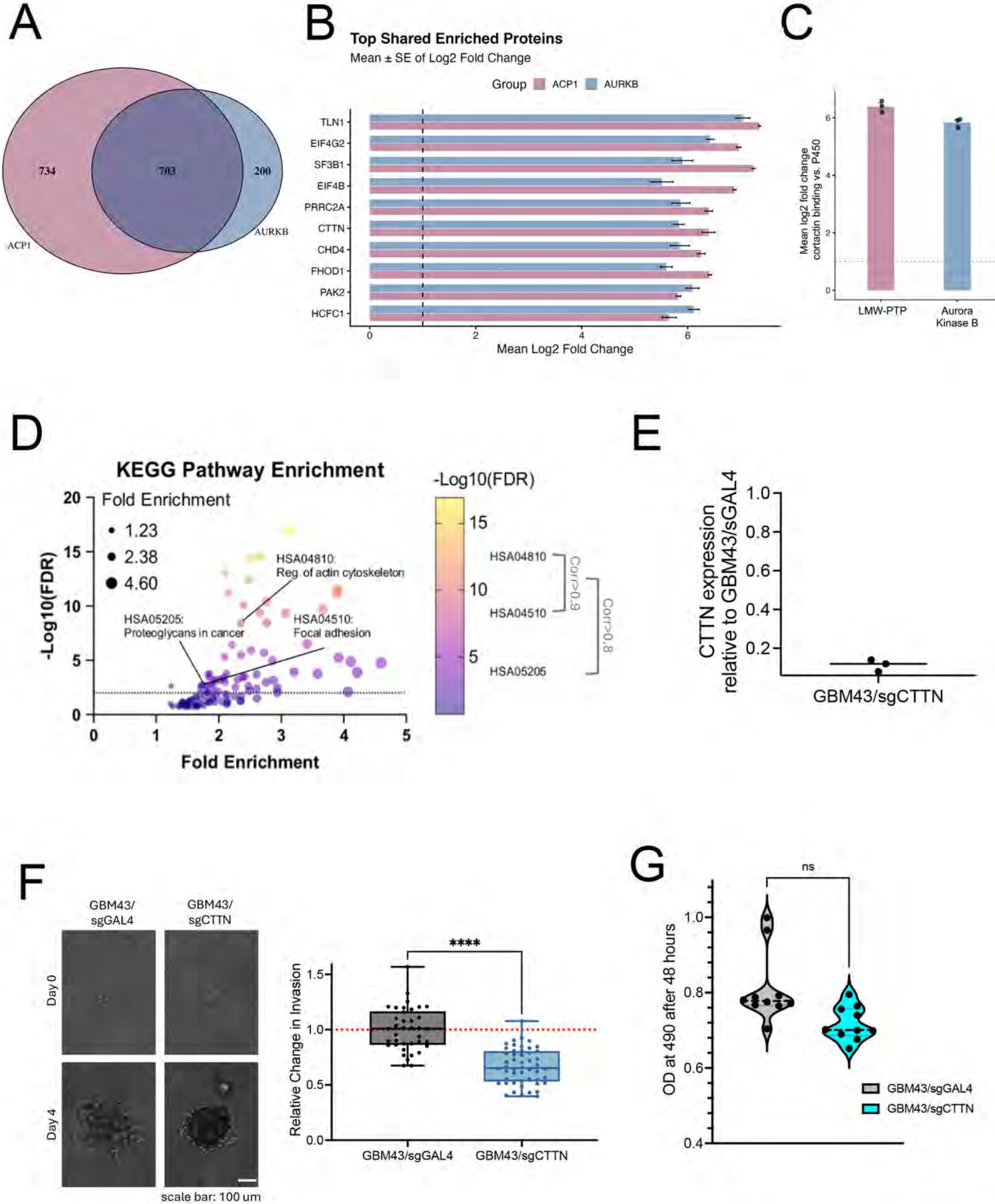

Supplemental Figure 3

Supplemental Figure 4. Serine phosphorylation of cortactin by AURKB and tyrosine dephosphorylation of cortactin by ACP1.

A. A sandwich ELISA was established using an anti-cortactin antibody for capture and an antibody against phosphorylated tyrosine for detection. Incubating recombinant cortactin with c-Src and ATP led to increased tyrosine phosphorylation of cortactin ($P<0.0001$) compared to reactions with cortactin and ATP but no c-Src.

B. Shown are the three technical replicates of immunoprecipitation in which cortactin was immunoprecipitated from GBM43 cell lysates followed by blotting for phosphorylated serine/threonine (row one), blotting for cortactin (row two), and blotting the non-precipitated lysates for β-actin (row three).

C. Shown are the three technical replicates of immunoprecipitation in which phosphorylated tyrosine was immunoprecipitated from GBM43 cell lysates followed by blotting for cortactin (row one) and blotting the non-precipitated lysates for cortactin (row two) and β-actin (row three).

D. Quantification of band intensities from 3 technical replicates of a Western blot for total cortactin levels confirmed *CTTN* knockdown in GBM43/sgCTTN cells relative to GBM43/sgGAL4 control cells and also revealed decreased cortactin levels in GBM43/sgAURKB cells relative to GBM43/sgGAL4 control cells, with a representative blot shown in **Figure 4F**.

ns=not significant, *$P<0.05$; **$P<0.01$; ***$P<0.001$; ****$P<0.0001$. One-way ANOVA with Tukey pairwise post-hoc comparisons.

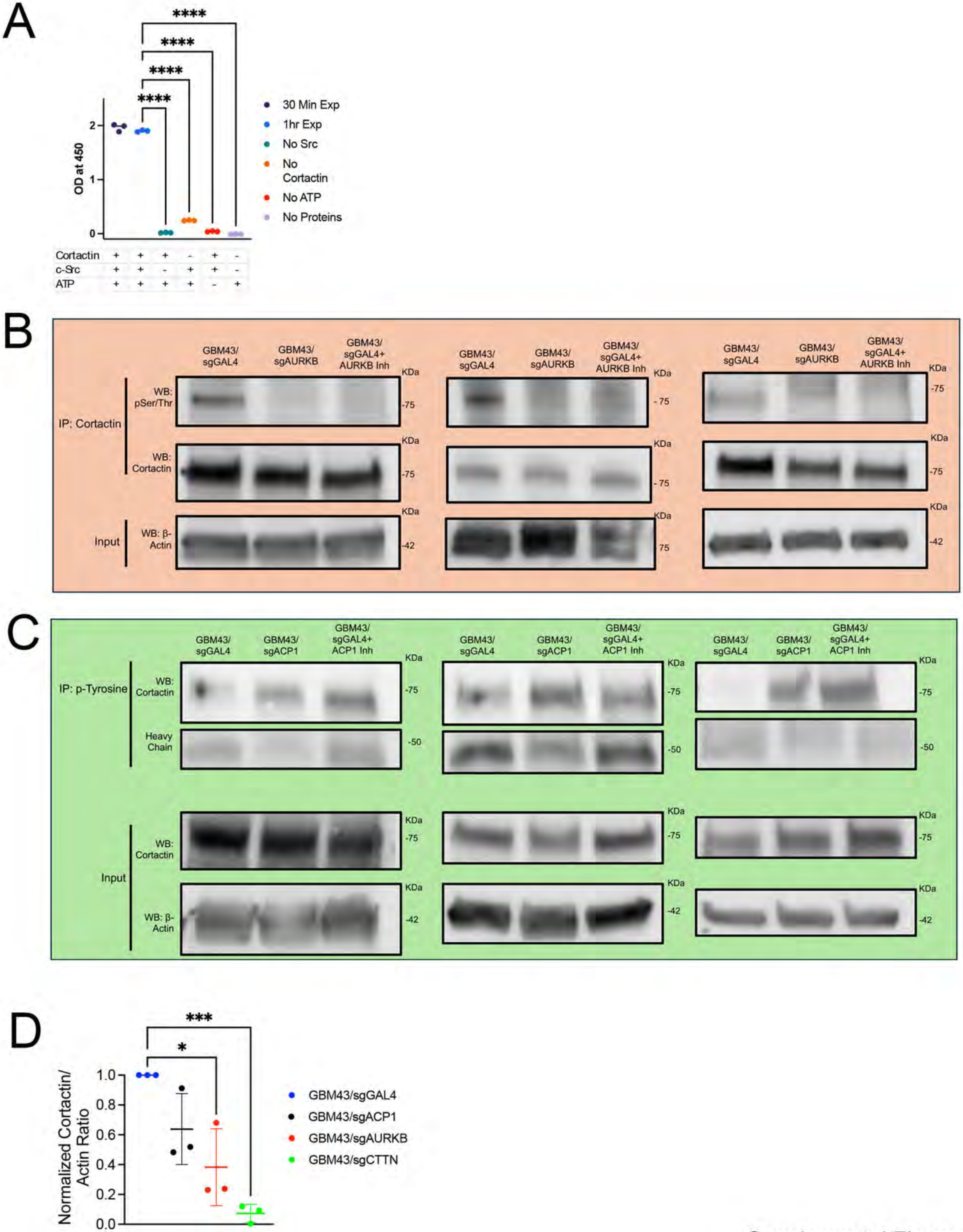

Supplemental Figure 4

Supplemental Figure 5. AURKB and ACP1 effects on cortactin phosphorylation alter cell morphology, actin polymerization, and mesenchymal gene expression.

A. Lysates from GBM43/sgGAL4 cells (left), GBM43/sgCTTN cells (left), GBM43/sgAURKB cells (middle), and GBM43/sgACP1 cells (right) were used in actin polymerization assays in the presence or absence of 1 ng/μL recombinant cortactin protein. The addition of cortactin protein elevated the levels and rate of actin polymerization seen in GBM43/sgCTTN, GBM43/sgAURKB, and GBM43/sgCTTN cells albeit not quite to the level seen in GBM43/sgGAL4 cells, whose levels and rate of actin polymerization were not affected by adding cortactin protein. Differences in polymerization levels in GBM43/sgCTTN, GBM43/sgAURKB, and GBM43/sgCTTN cells with or without adding cortactin protein are shown by blue asterisks below the x-axis, while differences in actin polymerization rates with or without cortactin protein are shown to the right of the corresponding curves. n=3/group. Points represent means, error bars represent standard deviations.

B. Targeting AURKB and ACP1 in GBM43/sgAURKB and GBM43/sgACP1, respectively, creates a more circular cell morphology ($P<0.0001$) that is less conducive to invasion, as assessed by measuring the aspect ratio using images (not shown) taken of cells grown in two-dimensional culture. n=63-67 cells/group.

C. *Left*: GBM43/sgAURKB ($P<0.0001$) and GBM43/sgACP1 ($P=0.002$) cells had smaller 3D volumes when placed into 3D HA-RGD devices than GBM43/sgGAL4 cells, while GBM43/sgCTTN cells were unchanged in size ($P=0.08$) (n=26-66 cells per condition). *Right*: GBM43/sgAURKB ($P=0.0001$), GBM43/sgACP1 ($P<0.0001$), and

GBM43/sgCTTN (P<0.0001) cells had smaller 2D areas compared to GBM43/sgGAL4 cells (n=78-84 cells per condition) when placed into 3D HA-RGD devices.

D. GBM43/sgAURKB (P=0.007) or GBM43/sgACP1 (P<0.0001) cells exhibited a reduction in the percentage of total cell area in the nucleus compared to GBM43/sgGAL4 cells. n=29 cells/group.

E. Quantification of Apotracker Green and Zombie Red Fixable Viability Dye staining performed 42 hours after plating cells revealed no difference in the percentage of total cells that are viable and non-apoptotic amongst GBM43/sgAURKB (P=0.1), GBM43/sgACP1 (P=0.7), or GBM43/sgCTTN (P=0.5) cells compared to GBM43/sgGAL4 cells. n=3/group.

F. Targeting AURKB with CRISPRi or drug (AZD1152-HQPA) in cultured GBM43 cells leads to reduced cortactin serine phosphorylation (based on overlapping area of cortactin and p-Ser/p-Thr staining) normalized to cell area in the total cell (P<0.0001 with CRISPRi or with drug), in the cell core (P<0.0001 with CRISPRi or with drug), and within 3 μm of the cell edge (P=0.008 with CRISPRi, P=0.007 with drug).

G. Targeting ACP1 with CRISPRi or drug (LMW-PTP Inhibitor I) in cultured GBM43 cells leads to increased cortactin tyrosine phosphorylation (based on overlapping area of cortactin and p-Tyr staining) normalized to cell area in the total cell (P=0.01 with CRISPRi, P=0.0005 with inhibitor) and cell core (P=0.01 with CRISPRi, P<0.0001 with inhibitor), but with no change within 3 μm of the cell edge (P=0.1 with CRISPRi, P=0.06 with inhibitor) due to higher background cortactin tyrosine phosphorylation at the cell edge.

H. Immunostaining for Arp3, the component of the Arp2/3 complex that binds cortactin, revealed (leftmost graph) decreased intracellular Arp3 levels in GBM43 cells with *AURKB* (P=0.03 whole cell; P=0.02 cytoplasm; P=0.1 nuclei) or *ACP1* (P<0.0001 in whole cell, cytoplasm, or nuclei) knockdown compared to control GBM43/sgGAL4 cells. GBM43 cells with *AURKB* or *ACP1* knockdown also exhibited (middle graph) decreased Arp3-cortactin overlap (P<0.0001) and (rightmost graph) decreased Arp3-actin overlap (P=0.009 *AURKB*, P=0.0003 *ACP1*) compared to control GBM43/sgGAL4 cells. One-way ANOVA with Tukey pairwise post-hoc comparisons.

I. GBM43/sgAURKB (P=0.004) and GBM43/sgACP1 (P=0.002) cells shifted their actin from the cytoplasm to the nucleus in a manner not seen with GBM43/sgGAL4 cells whose actin was evenly distributed between the cytoplasm and nucleus (P=0.9). n=5 cells/group.

J. qPCR revealed that *AURKB* or *CTTN* knockdown in GBM43 cells lowered the expression of mesenchymal genes *FoxC1* (P=0.0007 *AURKB*, P=0.04 *CTTN*), *FoxC2* (P<0.0001 *AURKB*, P=0.0007 *CTTN*) *SLUG* (P<0.0001 *AURKB* and *CTTN*), *SNAIL* (P=0.0008 *AURKB*, P=0.0003 *CTTN*), and *Zeb2* (P=0.0003 *AURKB*, P=0.01 *CTTN*) compared to control GBM43/sgGAL4 cells, while *ACP1* knockdown lowered the expression of *SLUG* (P<0.0001) and *FOXC1* (P=0.009) compared to control GBM43/sgGAL4 cells. n=3/group.

ns=not significant, *P<0.05; **P<0.01; ***P<0.001; ****P<0.0001. Linear regression for slope comparisons (A), t-test for timepoint comparisons (A), and one-way ANOVA with Tukey pairwise post-hoc comparisons (B-J).

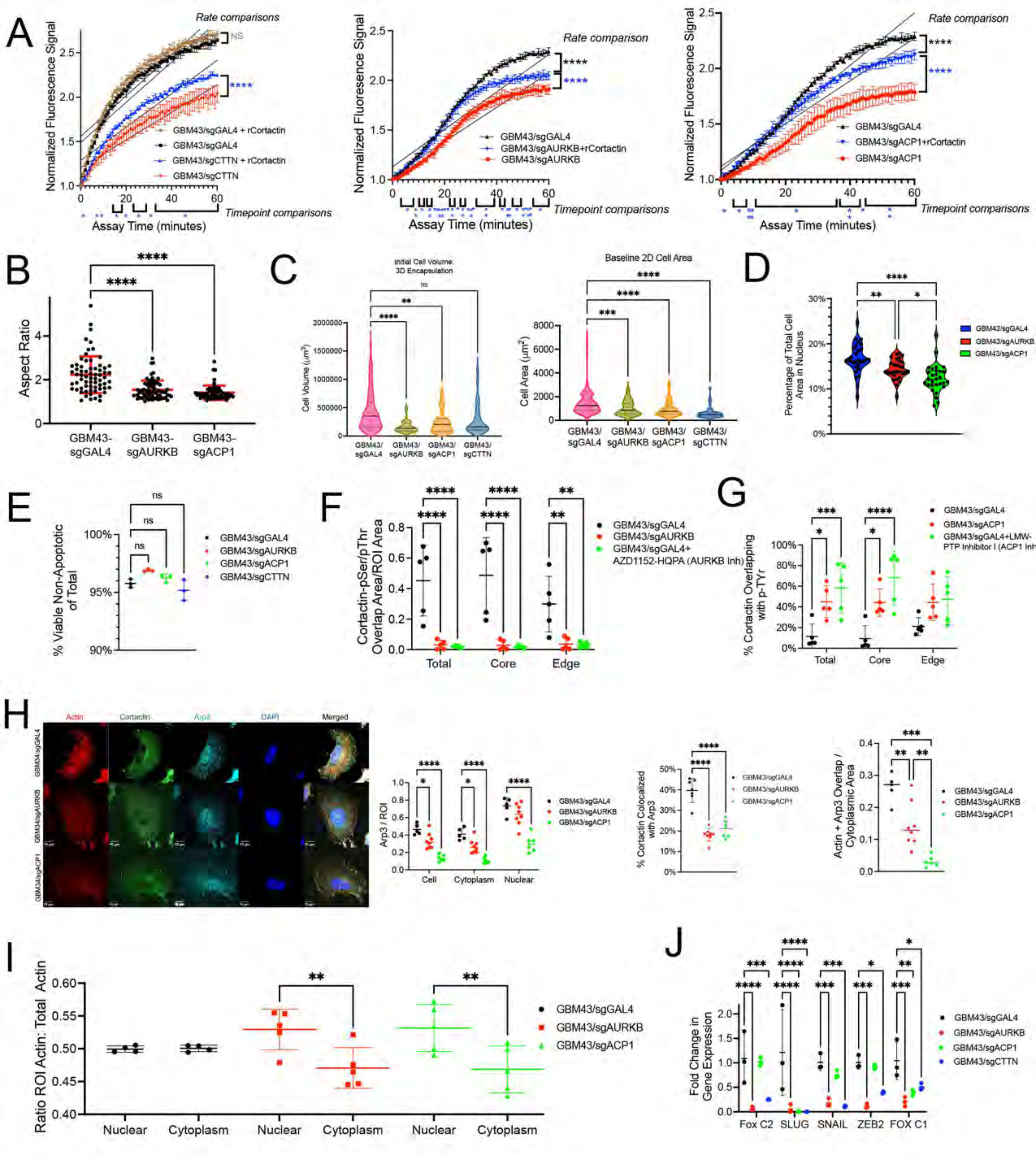

Supplemental Figure 5

Supplemental Figure 6. AURKB and ACP1 exert distinct effects on different aspects of GBM invasion.

A. Hydrogel storage modulus (G') (in Pascals) of HMW precast gels. Individual data points (n=3/group) for each frequency sweep are shown in red around a line connecting their means.

B. Stress relaxation curve showing degree of stress relaxation of material. The normalized relaxation modulus is obtained by normalizing points at all times to the modulus at 0 seconds. Individual data points (n=3/group) for each timepoint are shown in red around a line connecting their means.

C. U87 cell survival after treating cultured cells with varying concentrations of AURKB inhibitor AZD1152-HQPA (left) or ACP1 inhibitor LMW-PTP Inhibitor I (right) (n=3 biological replicates per concentration).

ns=not significant, *P<0.05; **P<0.01; ***P<0.001; ****P<0.0001

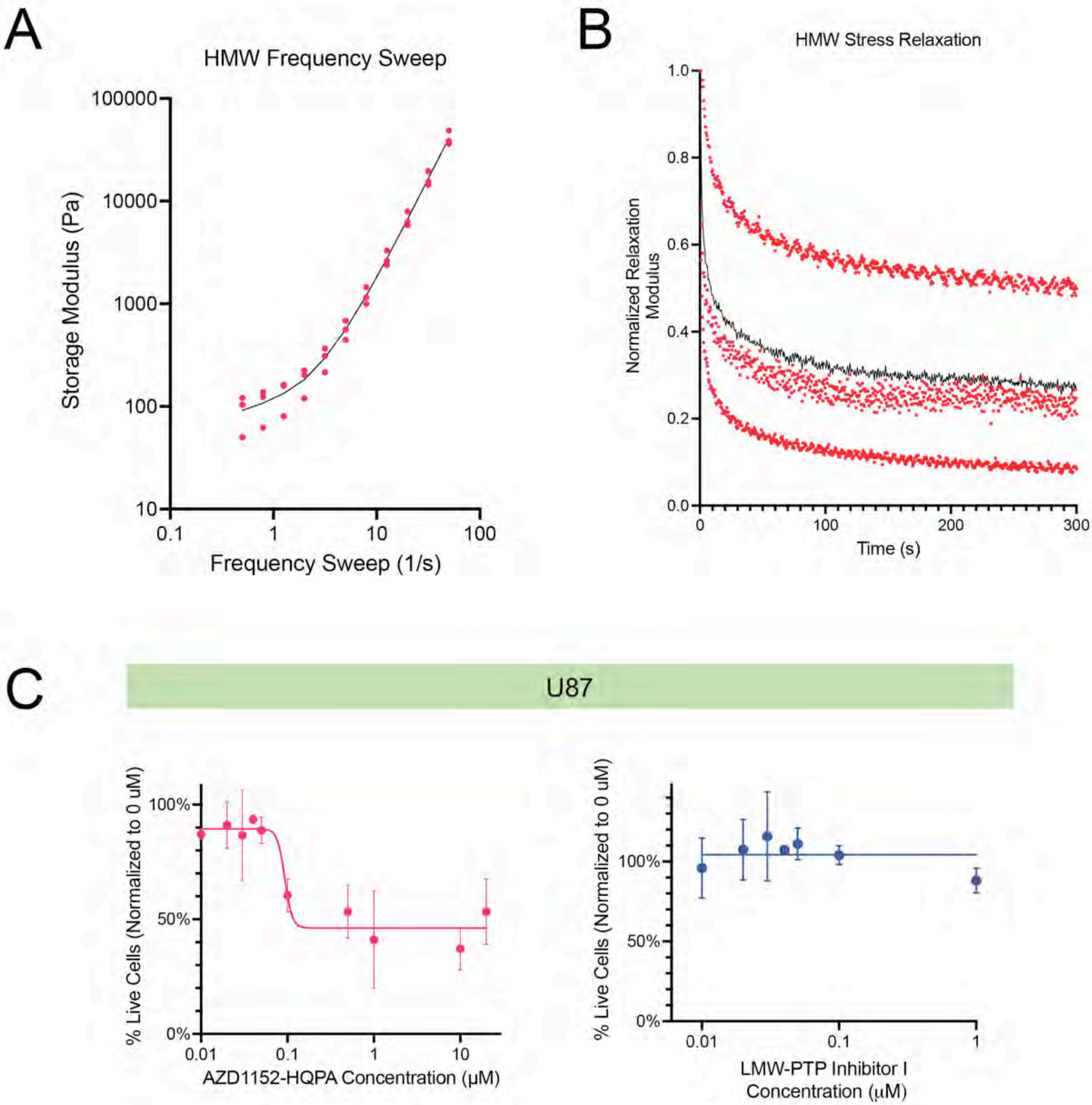

Supplemental Figure 6

Supplemental Figure 7. Targeting AURKB and ACP1 in orthotopic GBM xenografts *in vivo* uncouples cortactin from actin in tumor cells.

A. Immunofluorescence of xenografts at endpoint revealed decreased immunopositive cortactin area normalized to total area of cellular tumor in GBM43/sgAURKB (P=0.003, n=7) and GBM43/sgACP1 (P=0.005, n=6) xenografts relative to GBM43/sgGAL4 (n=6) xenografts, and virtually undetectable decreased immunopositive cortactin area normalized to cell number in GBM43/sgCTTN xenografts (n=4) relative to the other three groups (P=0.003 vs. GBM43/sgACP1, P=0.0007 vs. GBM43/sgAURKB, P=0.004 vs. GBM43/sgGAL4). Shown are images from **Figure 7D** alongside images form GBM43/sgCTTN xenografts that were not included in **Figure 7D**. 60x magnification, scale bar: 20 μm.

B. Tumor cells in GBM43/sgGAL4, GBM43/sgAURKB, and GBM43/sgACP1 xenografts had comparable intracellular cortactin distribution (P=0.7-0.9), with the portion of cortactin at the cell edge unchanged across all 3 groups.

ns=not significant, *P<0.05; **P<0.01; ***P<0.001; ****P<0.0001. One-way ANOVA with Tukey pairwise post-hoc comparisons.

A

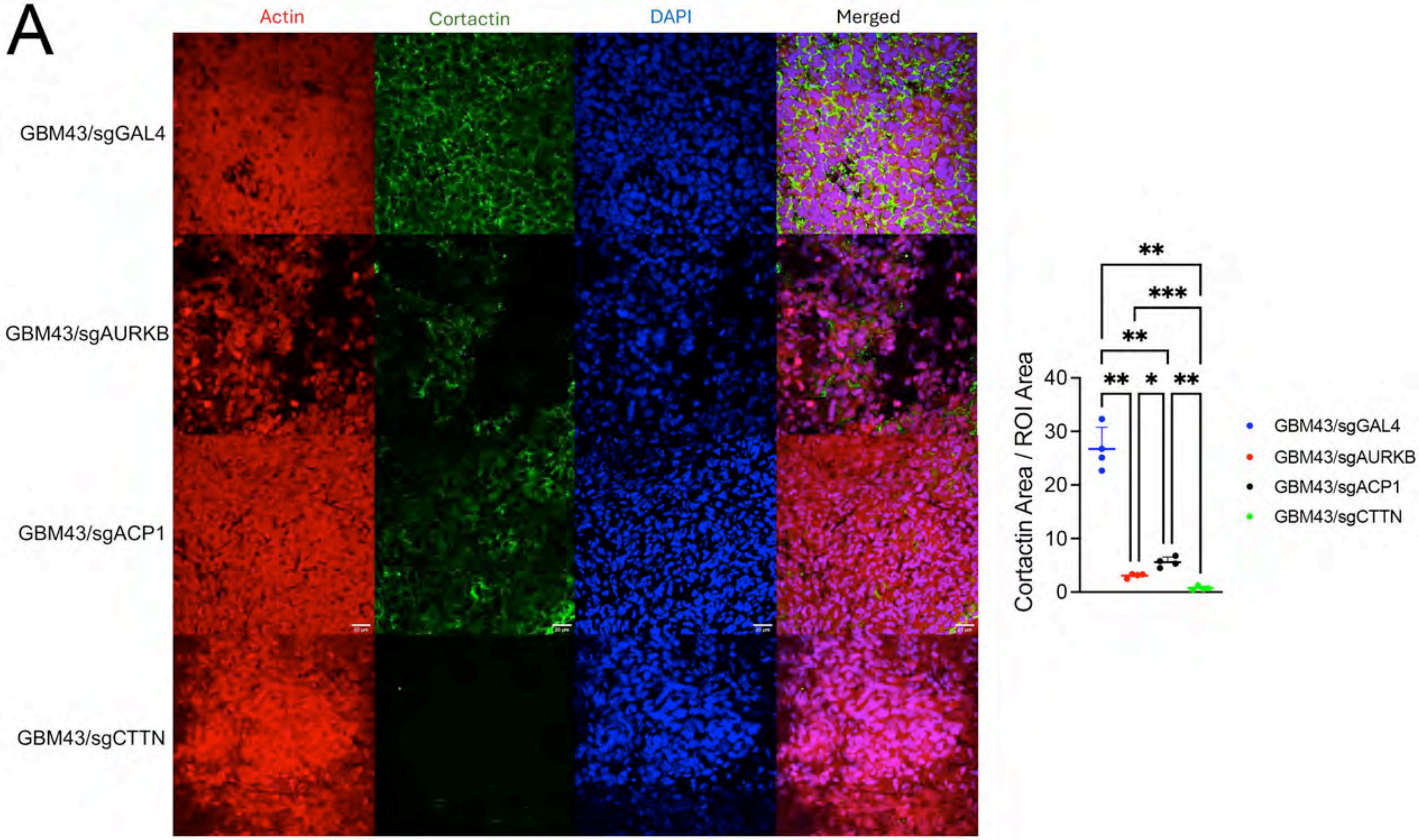

B

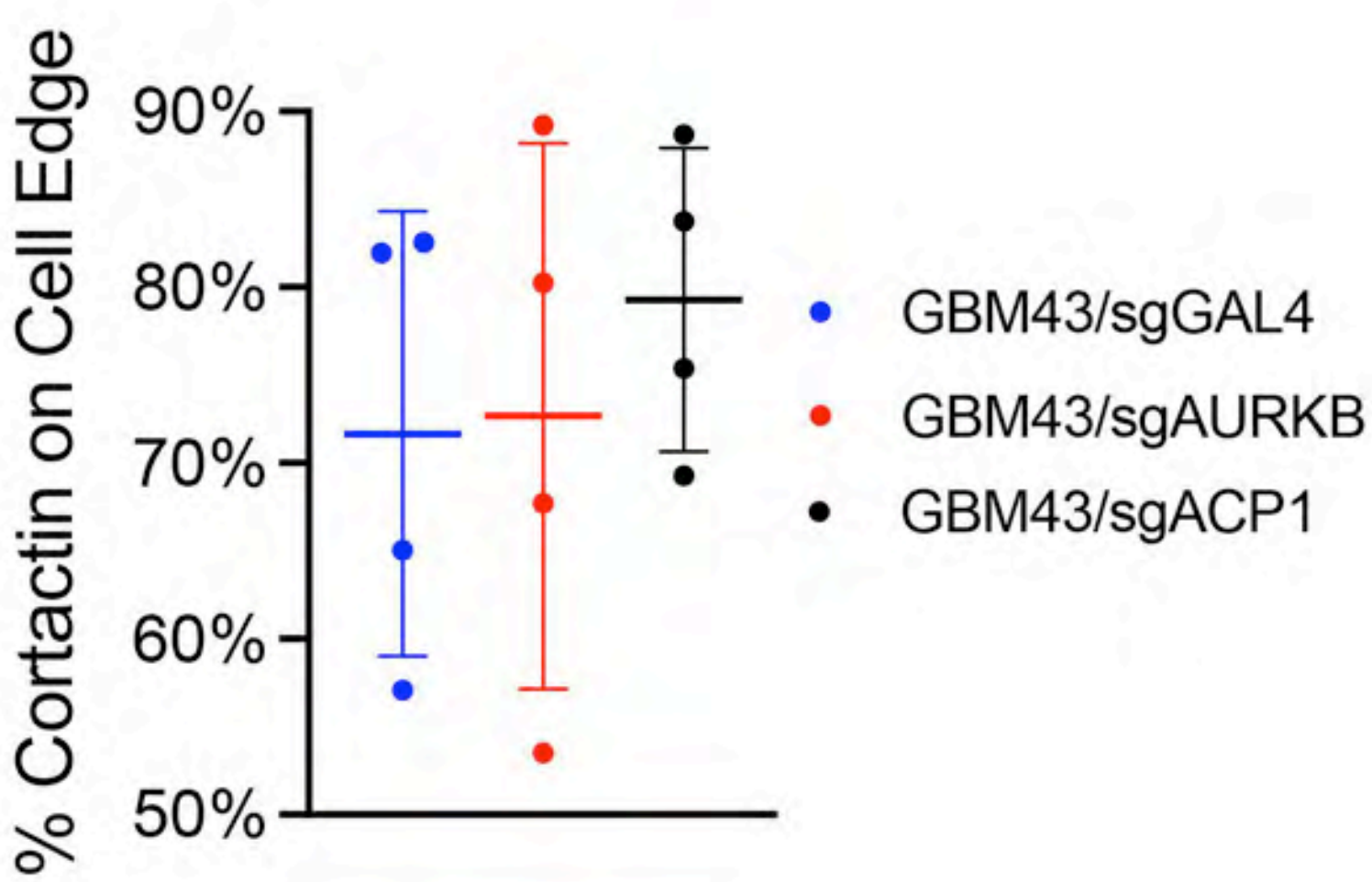

Supplemental Figure 7

Supplemental Video 1. Time-lapse videos of GBM43 cells under various treatment conditions migrating into high molecular weight hydrogels. Shown are videos of GBM43 cells migrating into high molecular weight (HMW) hydrogels while being treated with vehicle, 0.25 μM AURKB inhibitor AZD1152-HQPA, or 2 μM ACP1 inhibitor LMW-PTP Inhibitor I. While AZD1152-HQPA does not disrupt the leader-follower dynamics observed with vehicle treatment, LMW-PTP Inhibitor I reduces the number of leader cells, in turn leading to fewer followers and fewer cases escaping the channel (which represents the tumor core). This finding is consistent with the quantification of leader cells under all three conditions presented in **Figure 6D**. Scale bar 500 μm. Imaging was done over 18 hours with an image taken every 10 minutes.
