# Supplementary material for: Druggable genome CRISPRi screen in hydrogels reveals regulators of cortactin-driven actin remodeling promoting glioblastoma invasion
Source: JCI Insight. 2026 Aug 10;11(15):e196484. doi: 10.1172/jci.insight.196484 (PMC13463607; doi:10.1172/jci.insight.196484)

# Figure 4A IP: Cortactin WB-AURKB

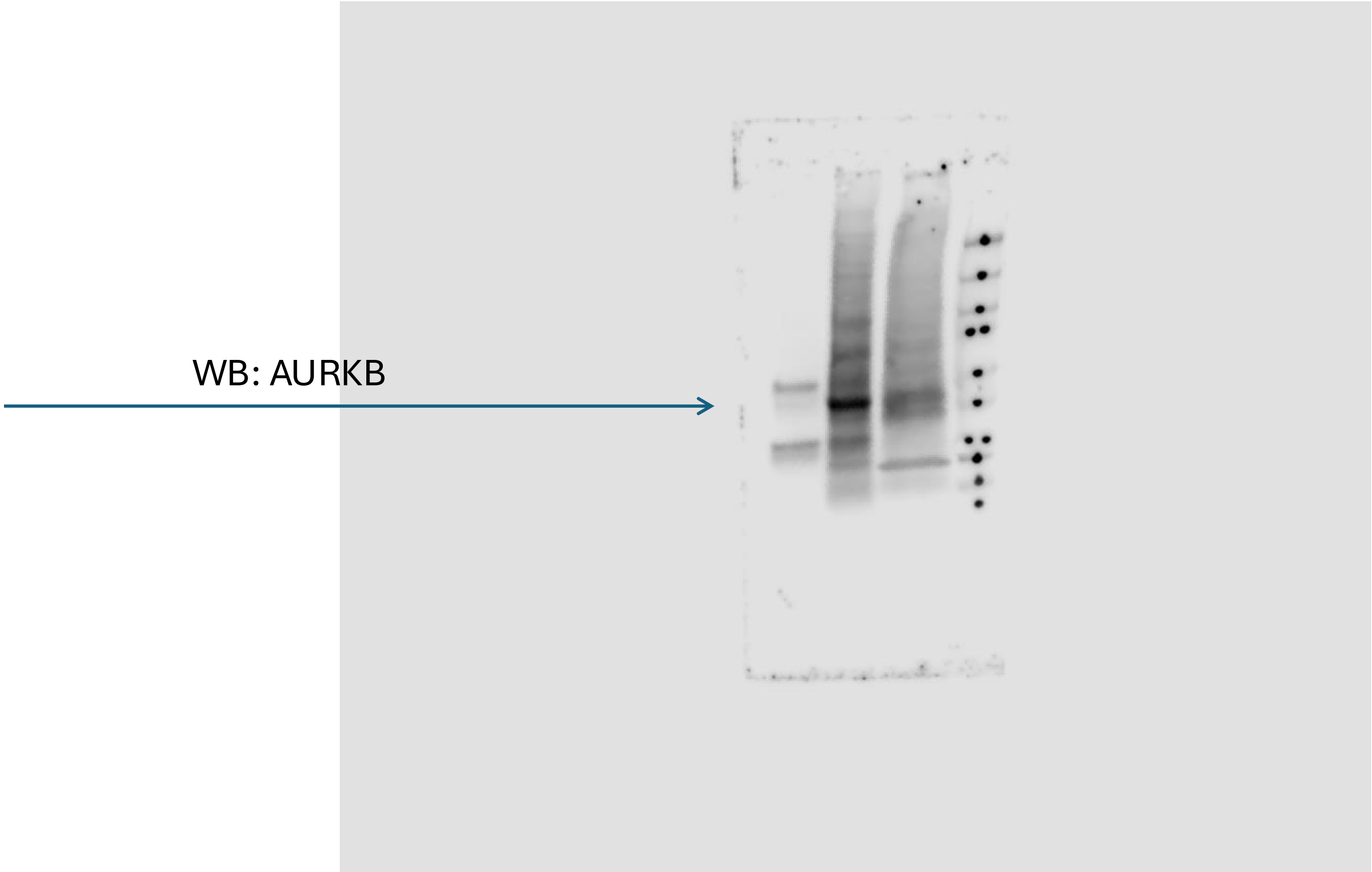

Figure 4A IP: Cortactin WB-ACP1

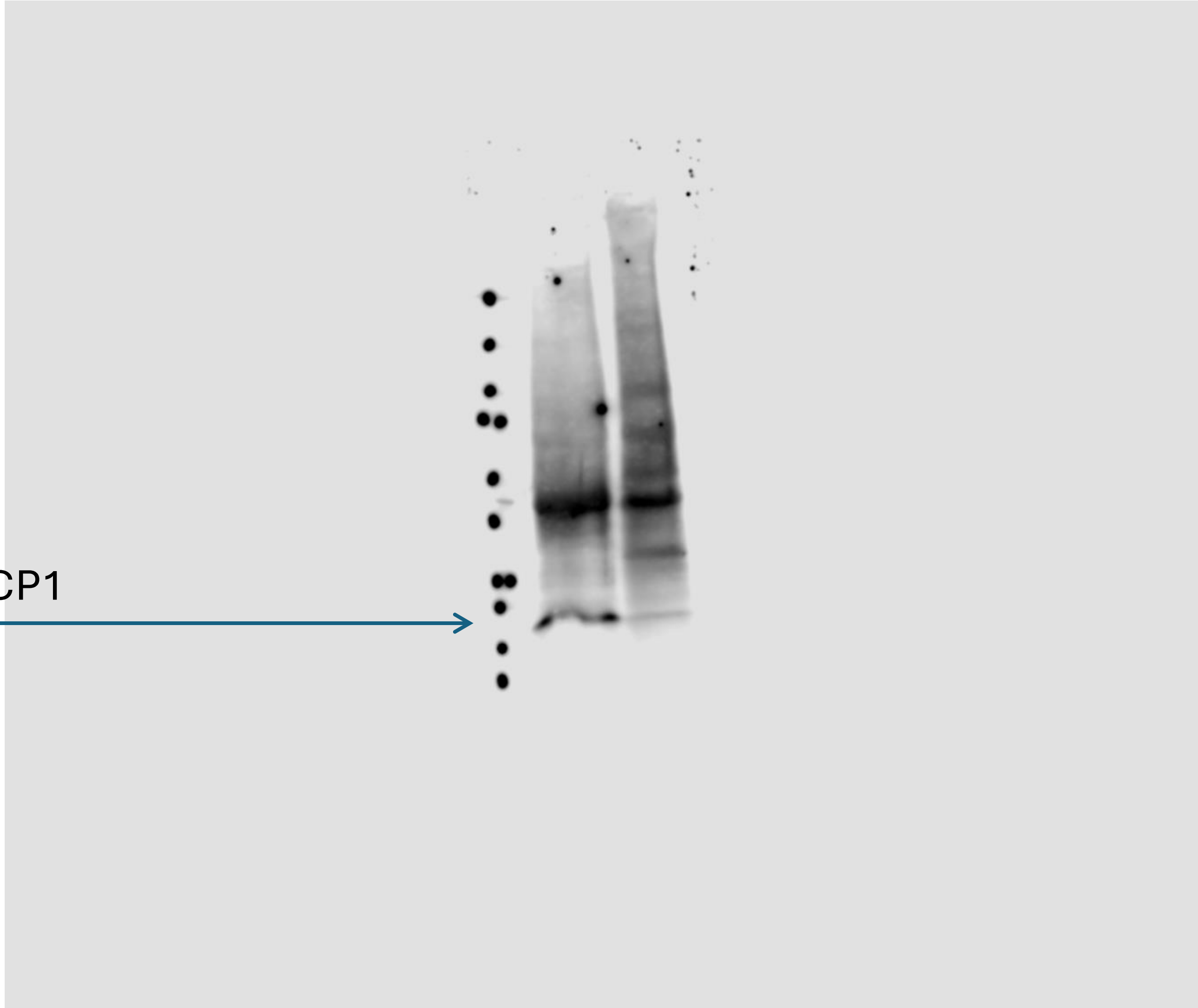

WB: ACP1

Figure 4A IP: ACP1-WB: Cortactin

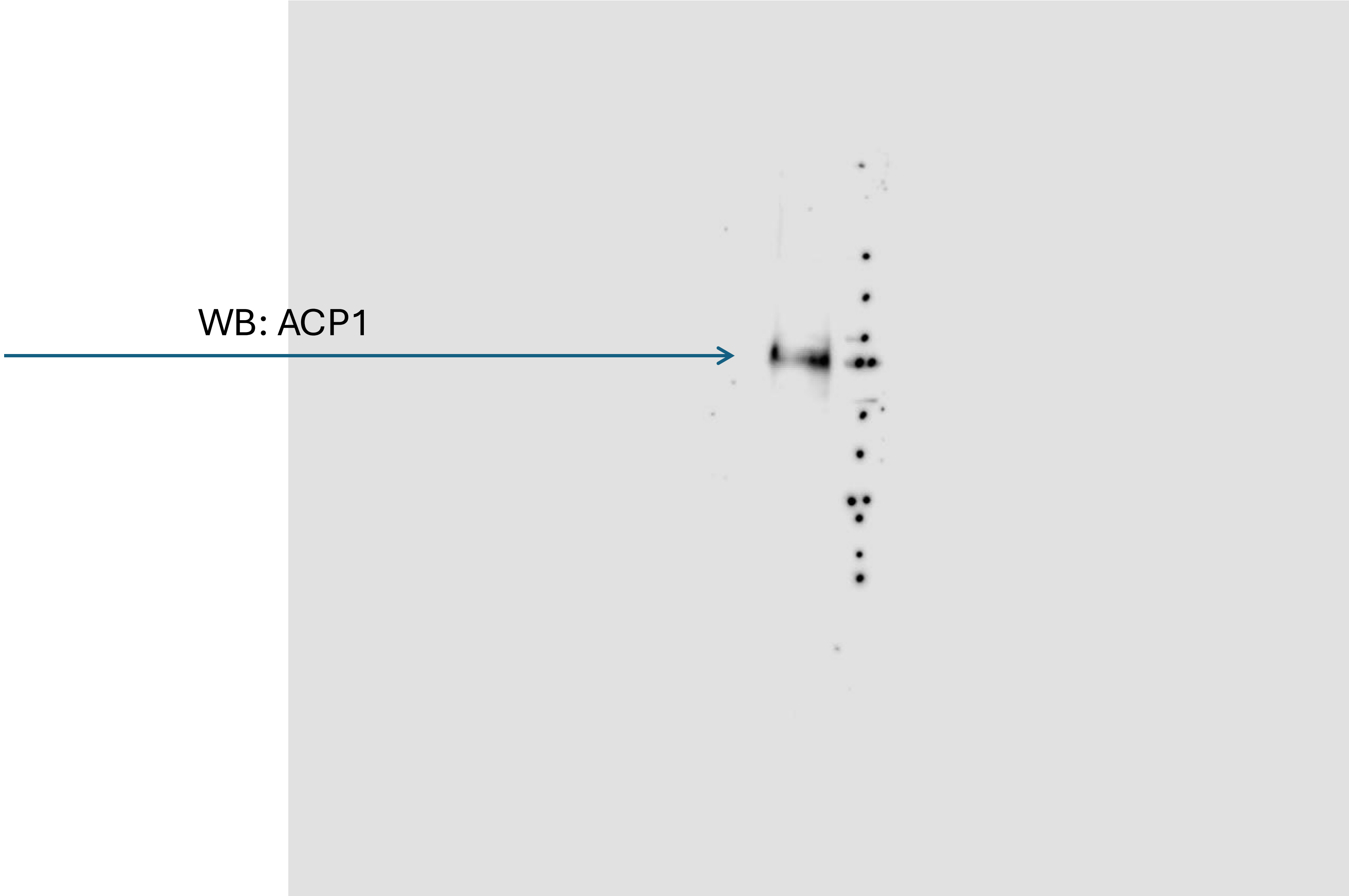

Fig4D row 1
(also Supp Fig 4B middle blots row 1)

IP: Cortactin
WB: pSer/pThr
Replicate #2

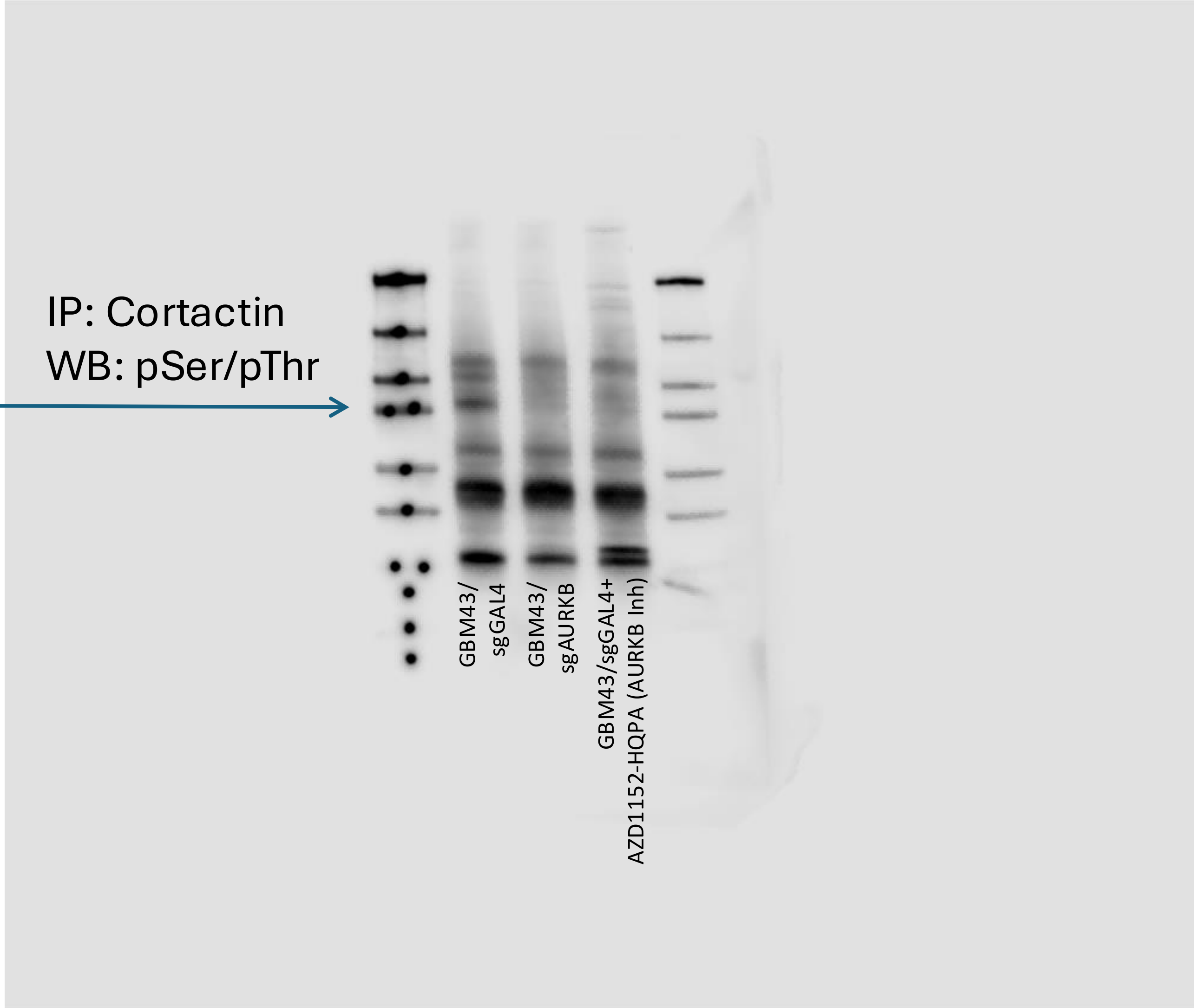

IP: Cortactin
WB: Cortactin
Replicate #2

Fig4D second row
(also Supp Fig 4 B middle blots row 2)

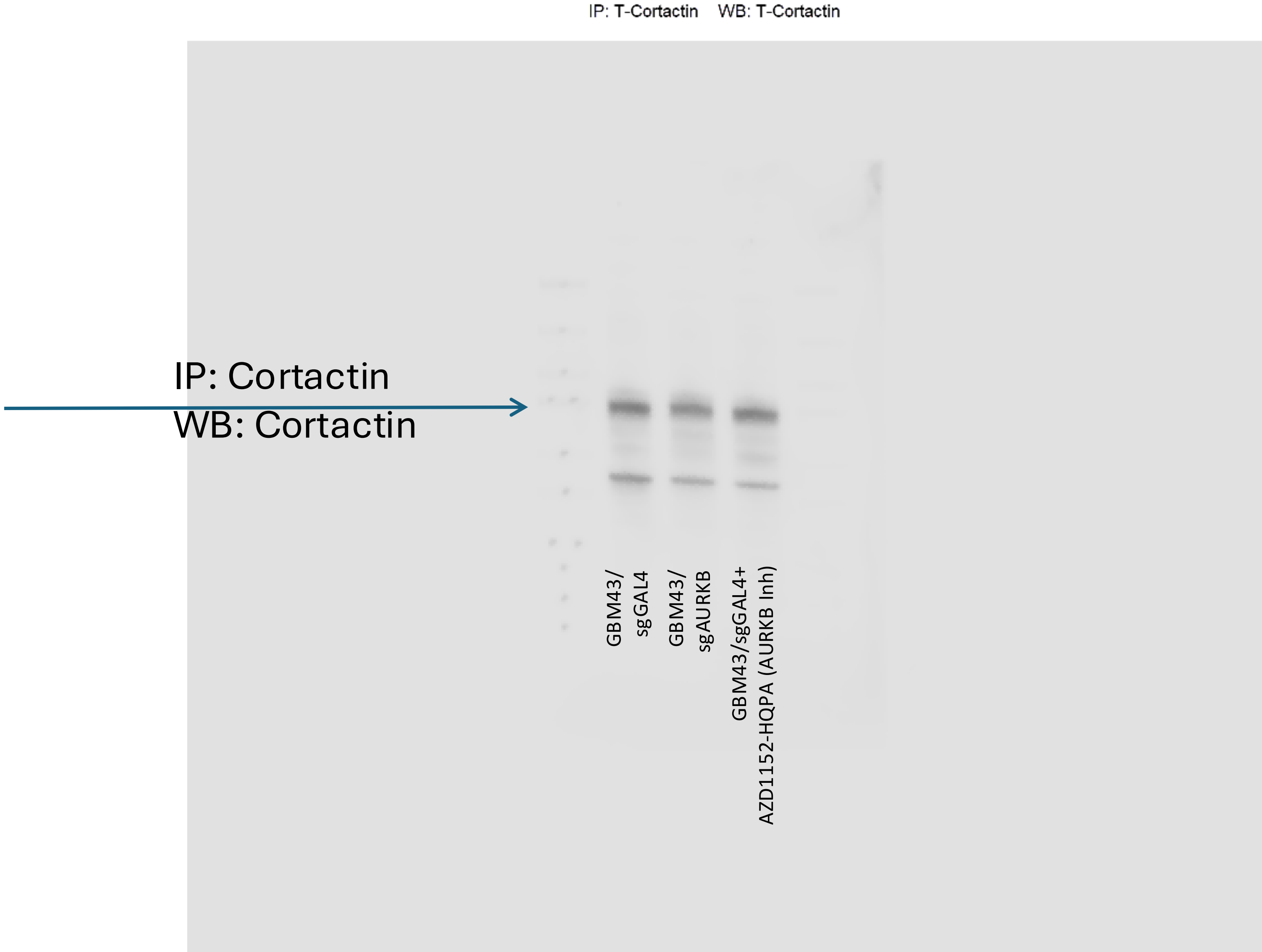

Figure 4D third row

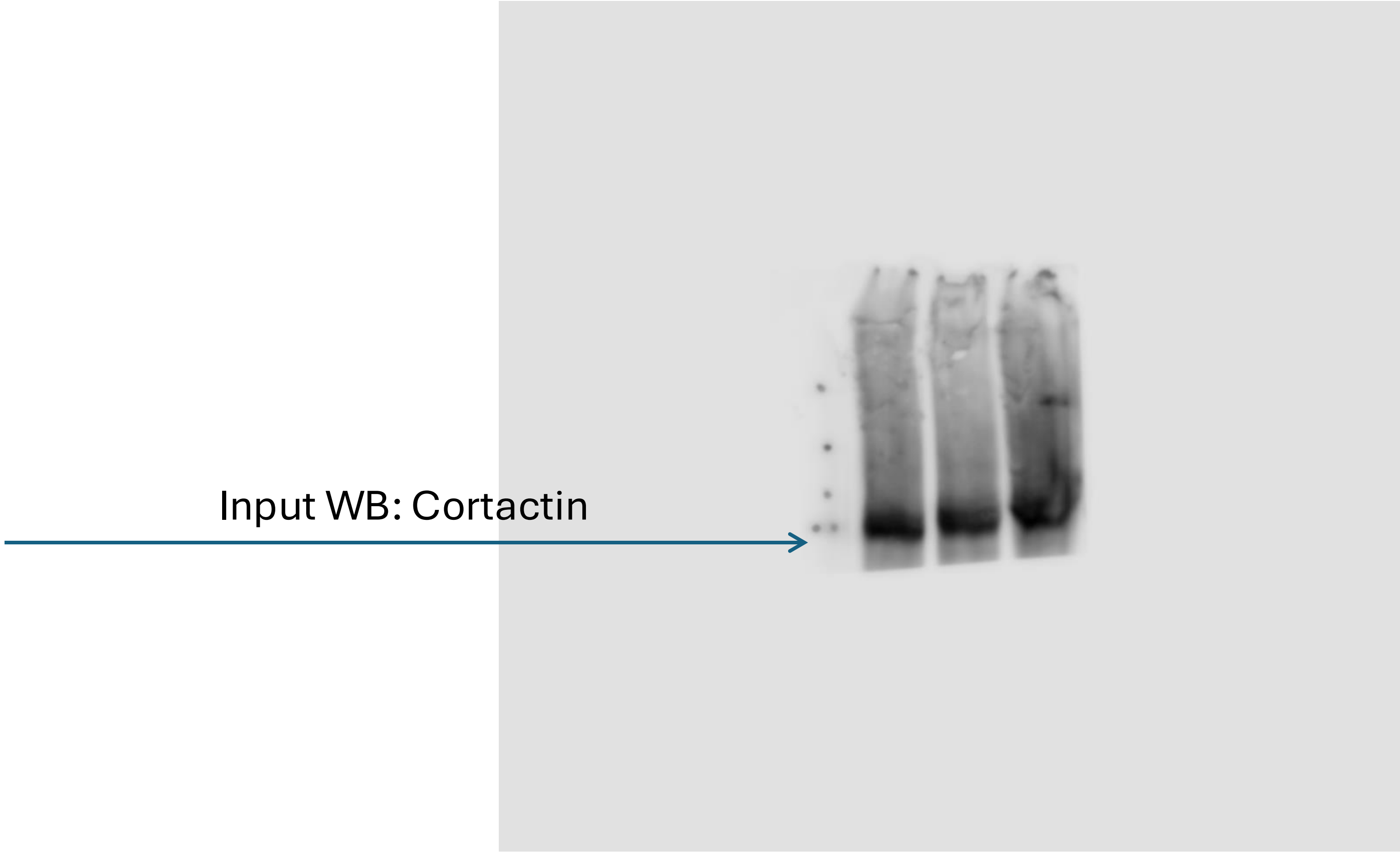

Input
WB: β-actin
Replicate #2

Fig4D fourth row
(also Supp Fig 4 B middle blots row 3)

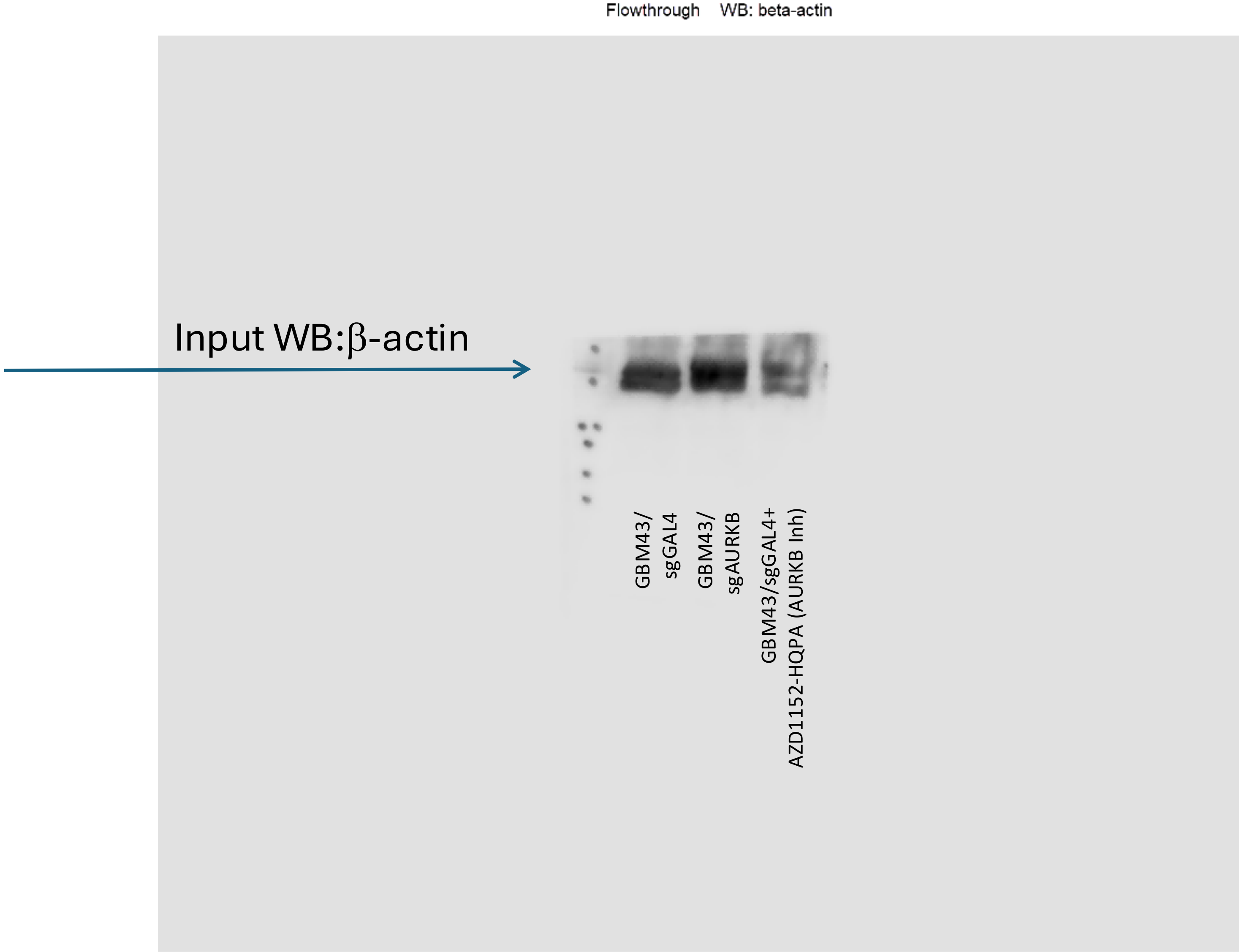

IP: p-Tyrosine
WB: Cortactin and Heavy Chain
Replicate #1

Fig 4E rows 1 and 2
(also Supp Fig 4 C left blots rows 1 and 2)

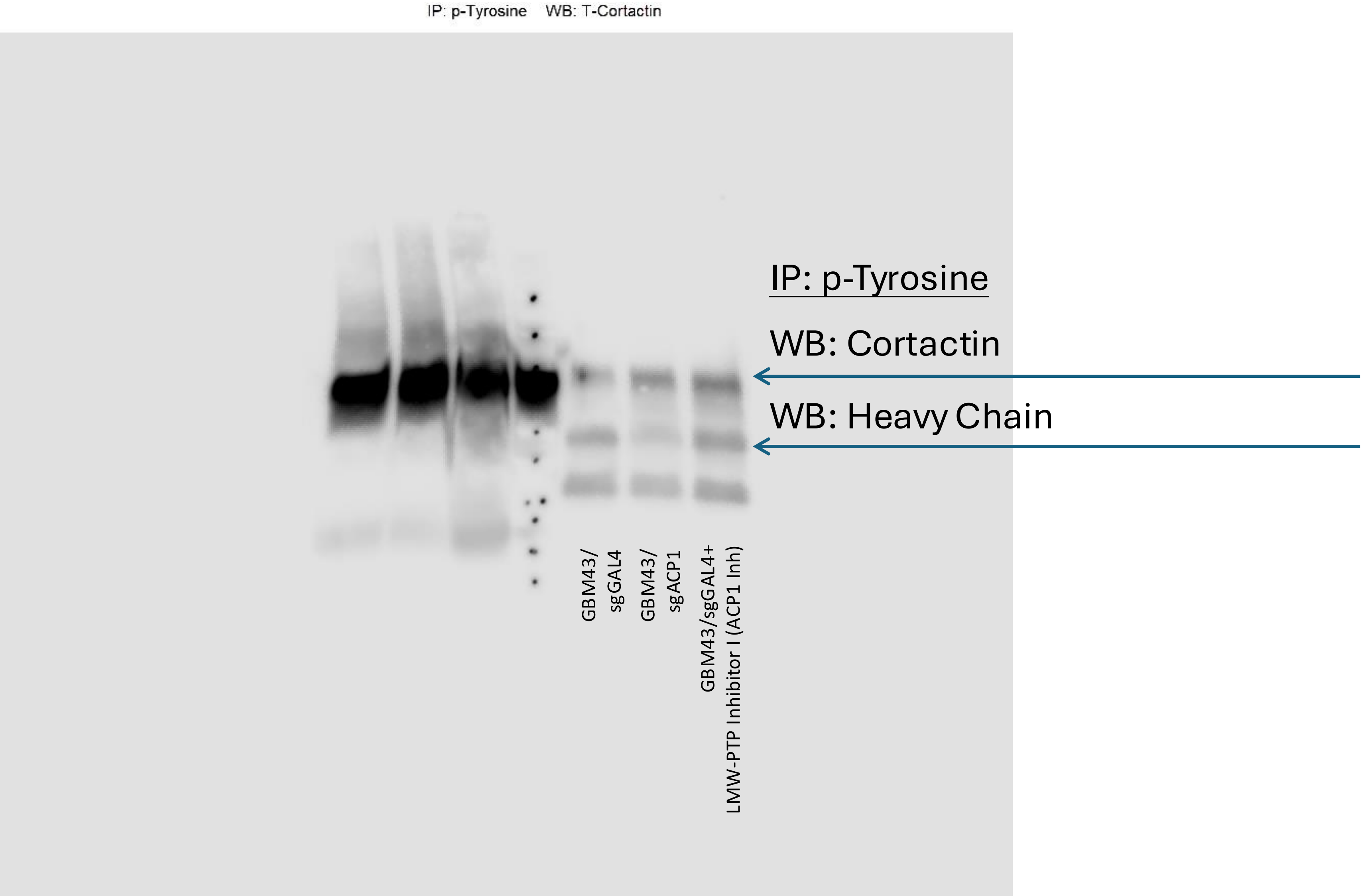

Input
WB: Cortactin
Replicate #1

Fig 4E row 3
(also Supp Fig 4 C left blots row 3)

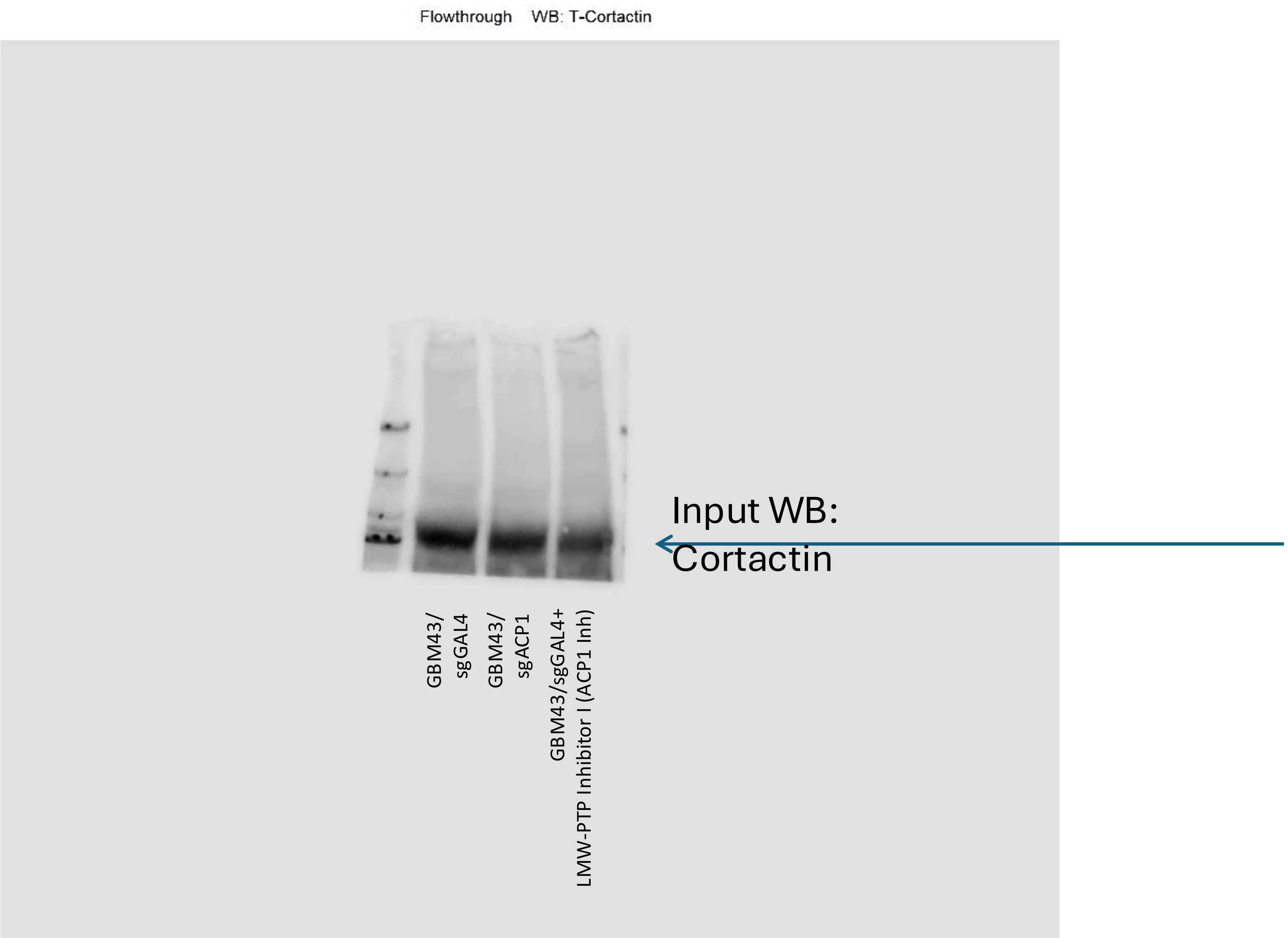

Fig 4E row 4
(also Supp Fig 4 C left blots row 4)

Input
WB: β-actin
Replicate #1

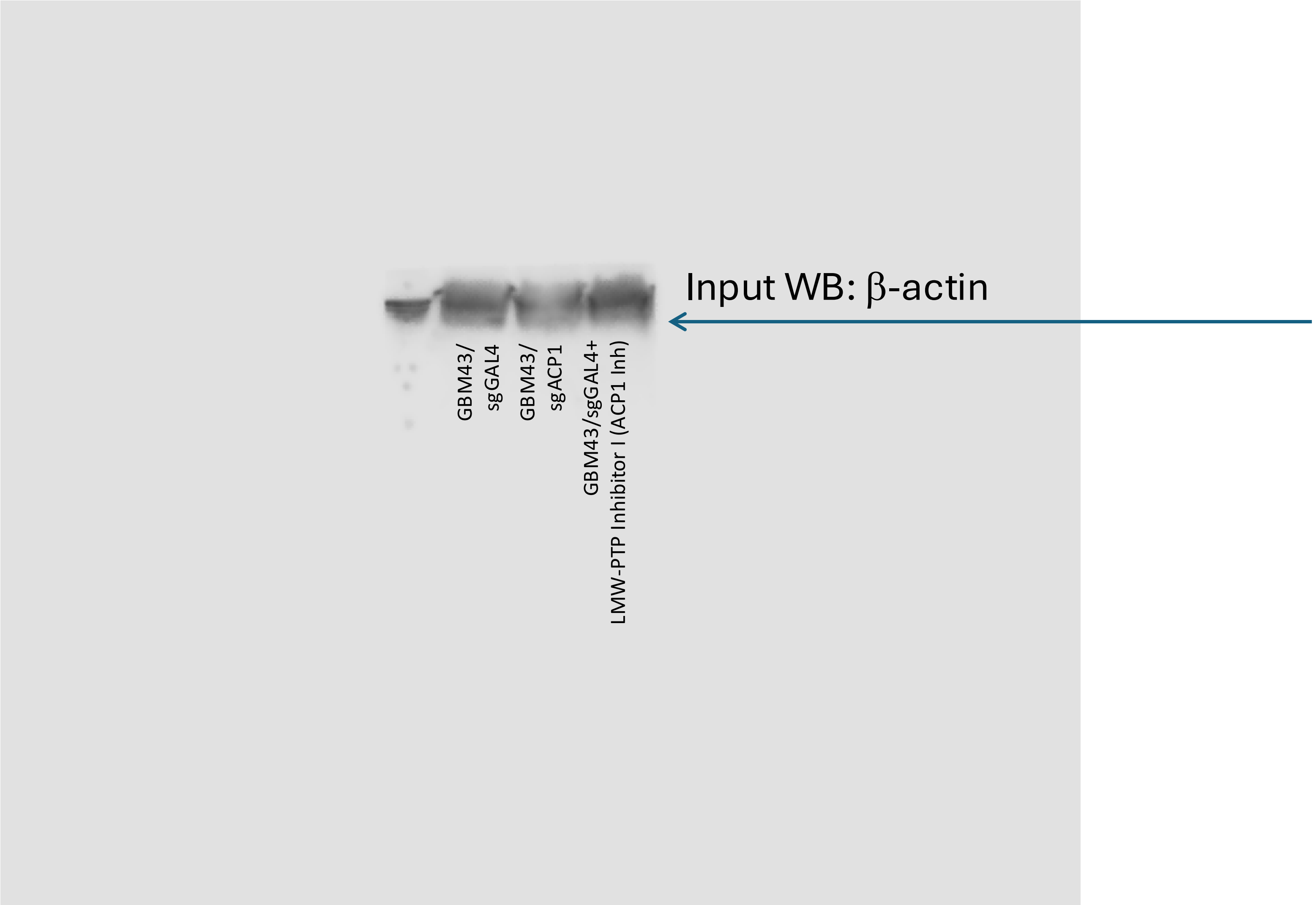

Figure 4F

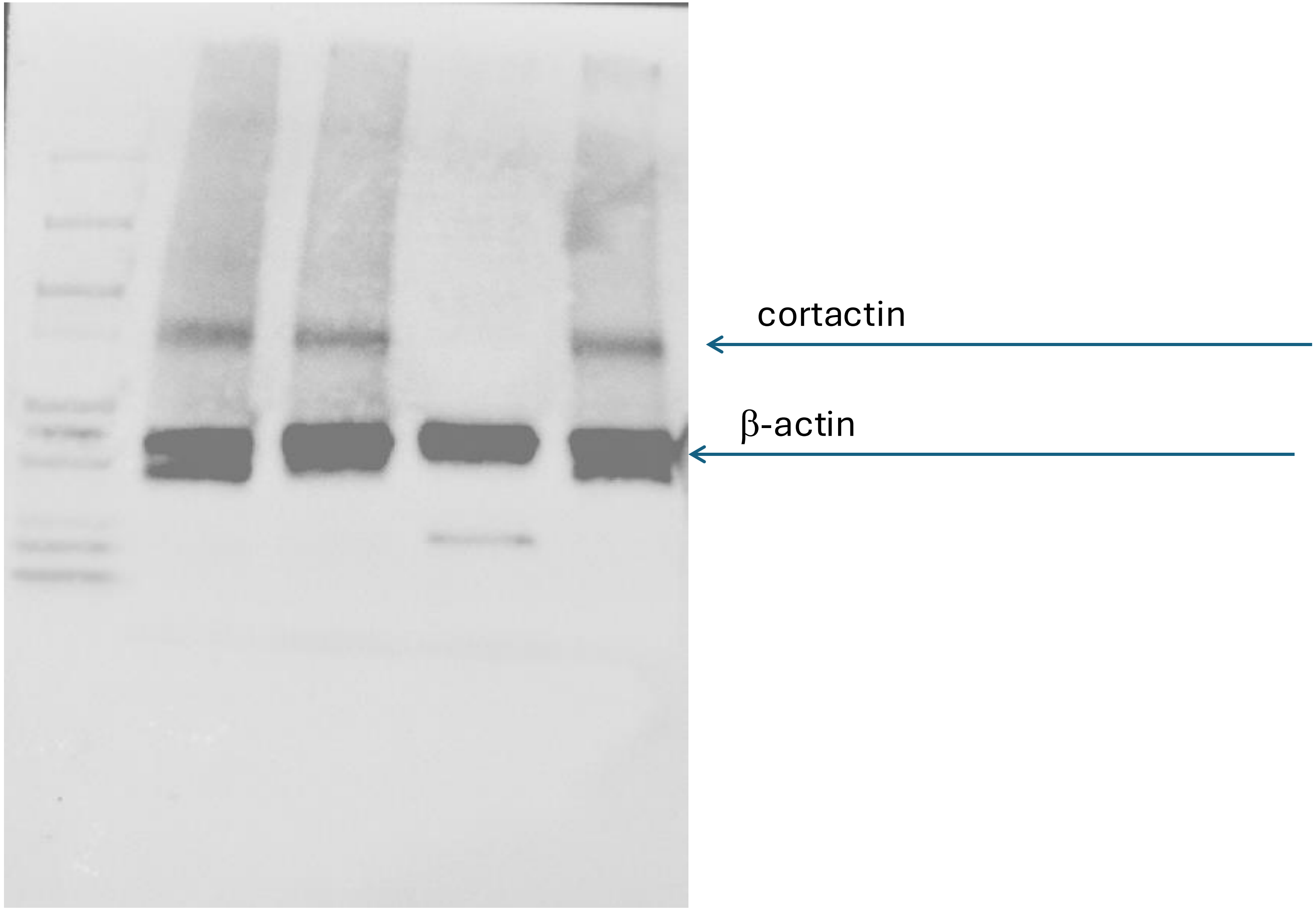

Supp Fig 4 B left blots row 1

IP: Cortactin
WB: pSer-pThr
Replicate #1

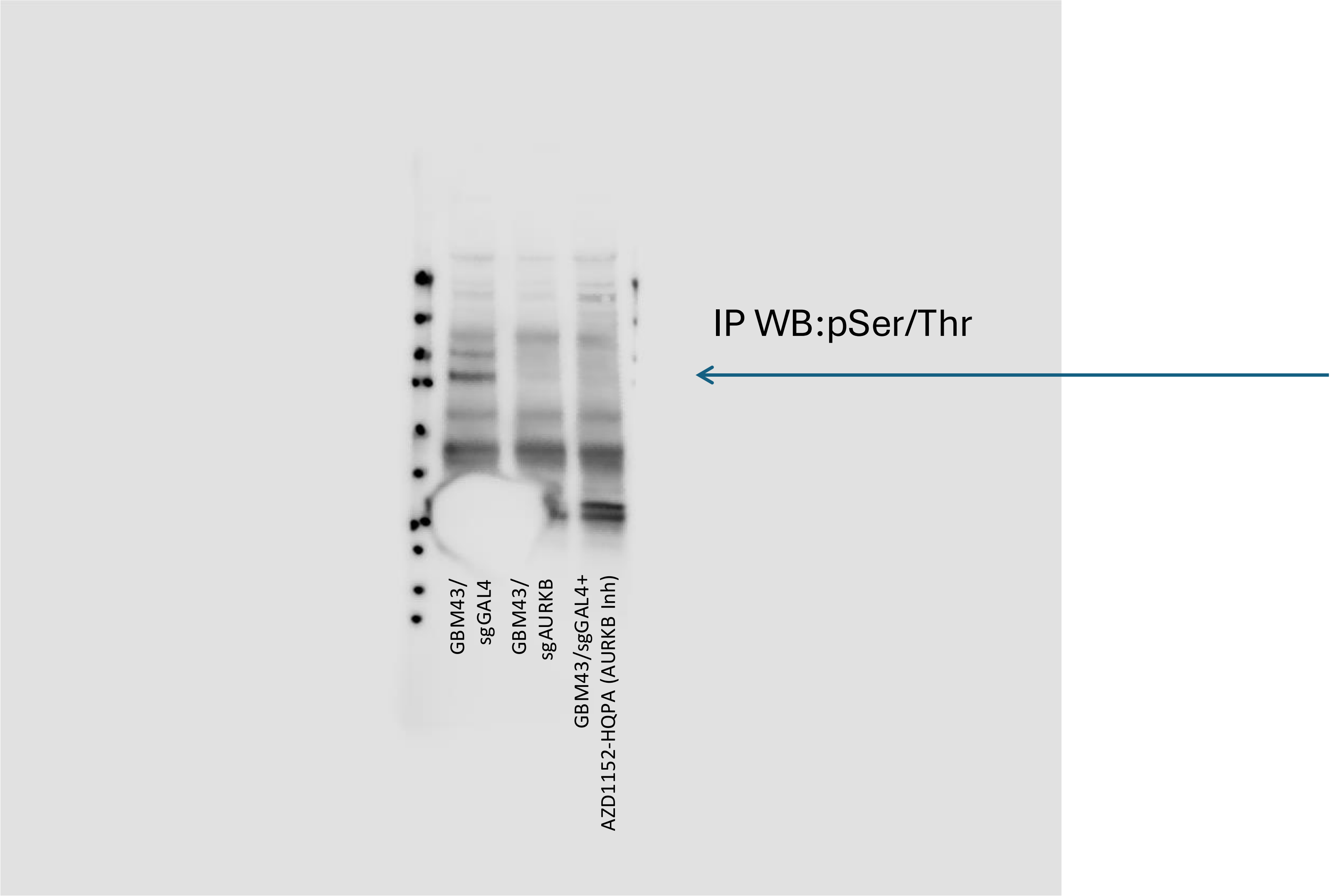

Supp Fig 4 B left blots row 2

IP: Cortactin
WB: Cortactin
Replicate #1

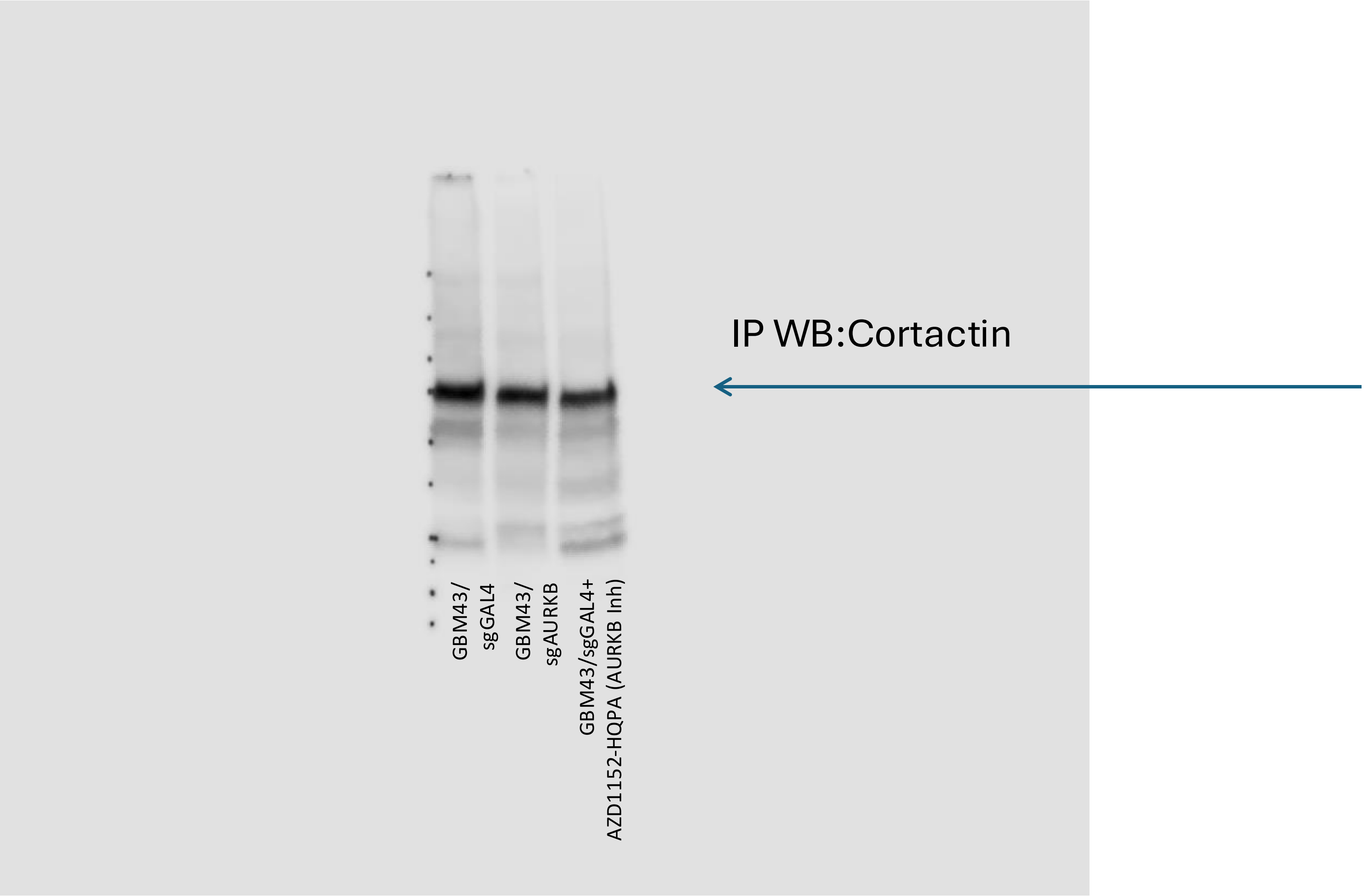

Supp Fig 4 B left blots row 3

Input
WB: β-actin
Replicate #1

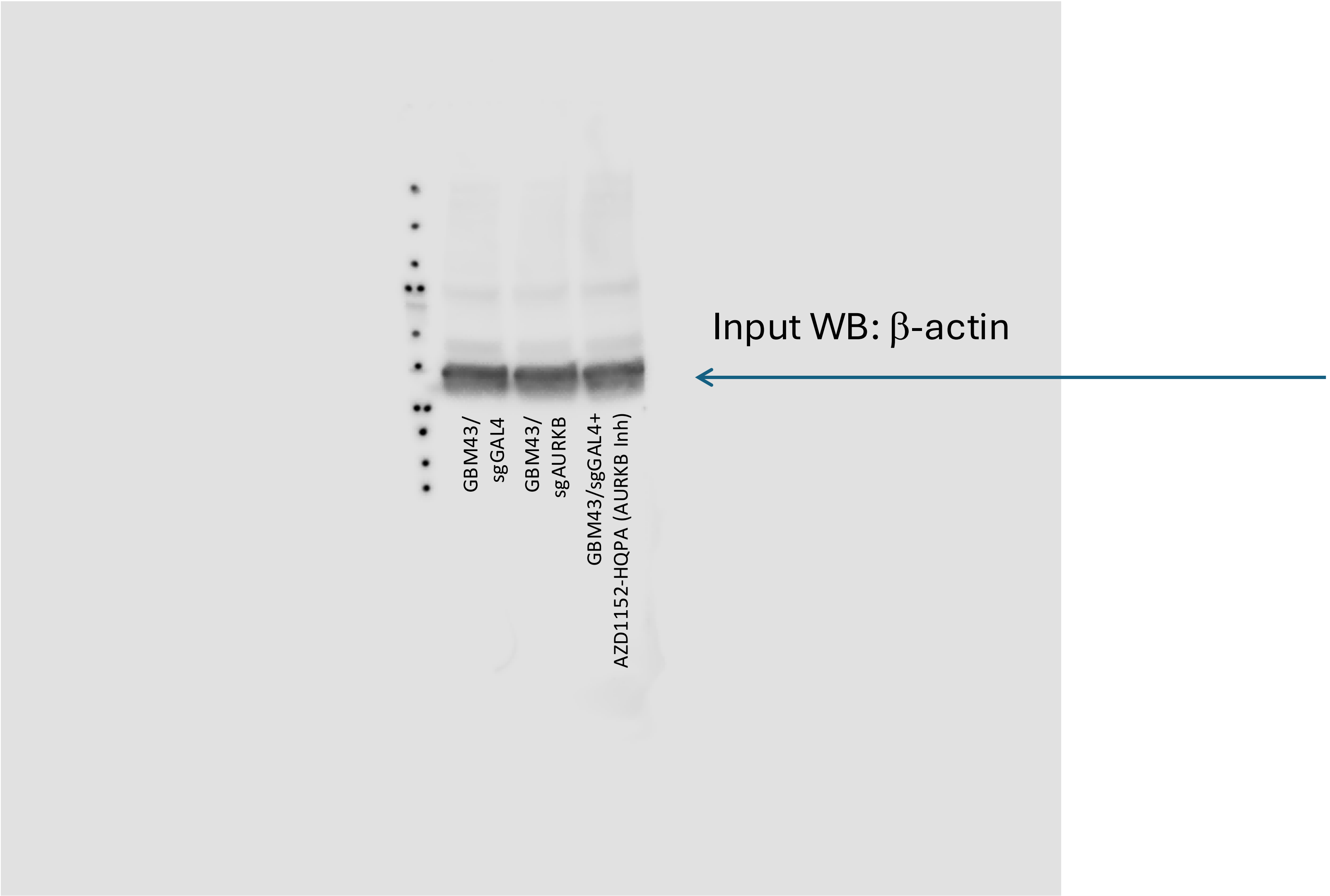

Supp Fig 4 B right blots row 1

IP: Cortactin
WB: pSer-pThr
Replicate #3

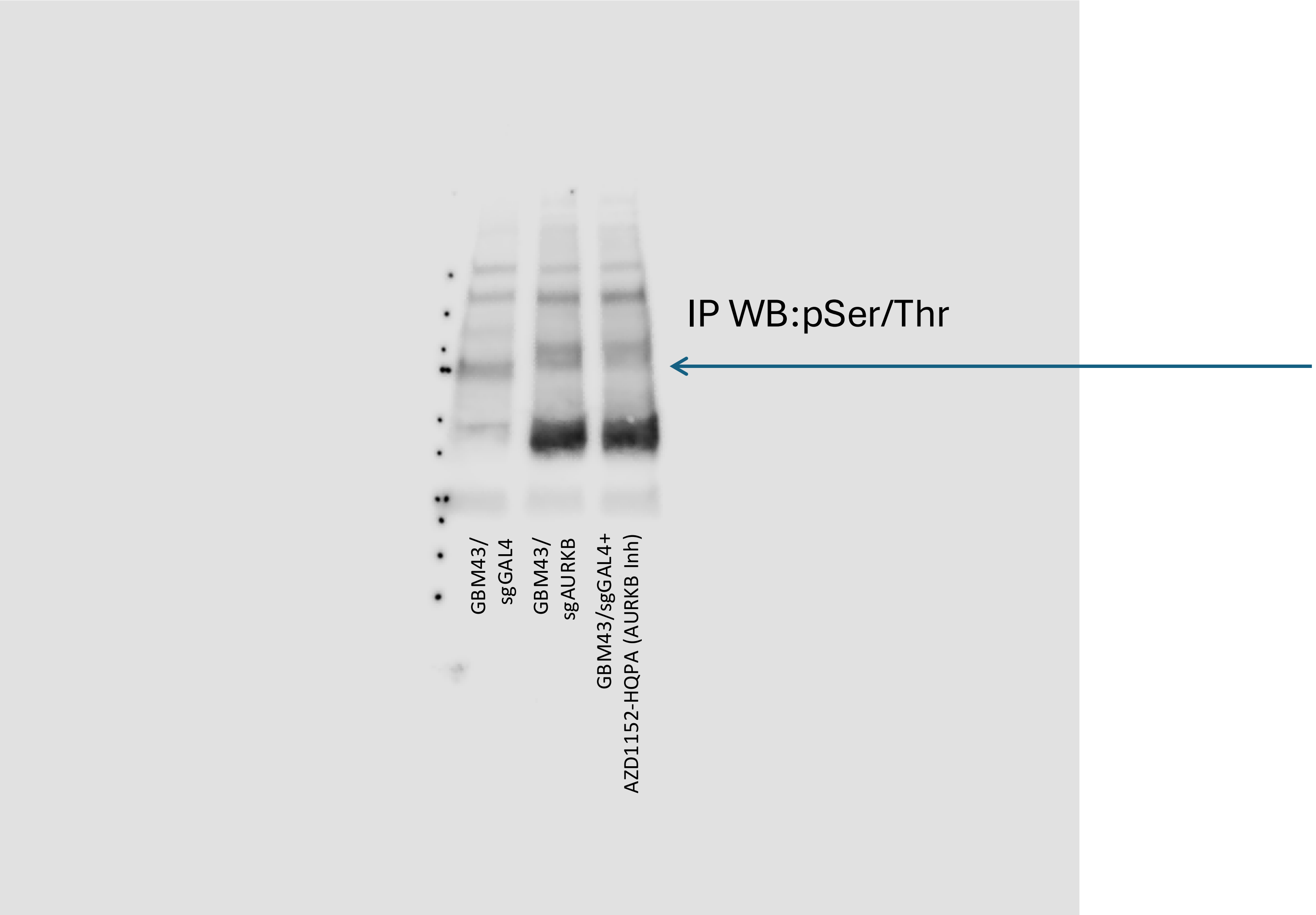

Supp Fig 4 B right blots row 2

IP: Cortactin
WB: Cortactin
Replicate #3

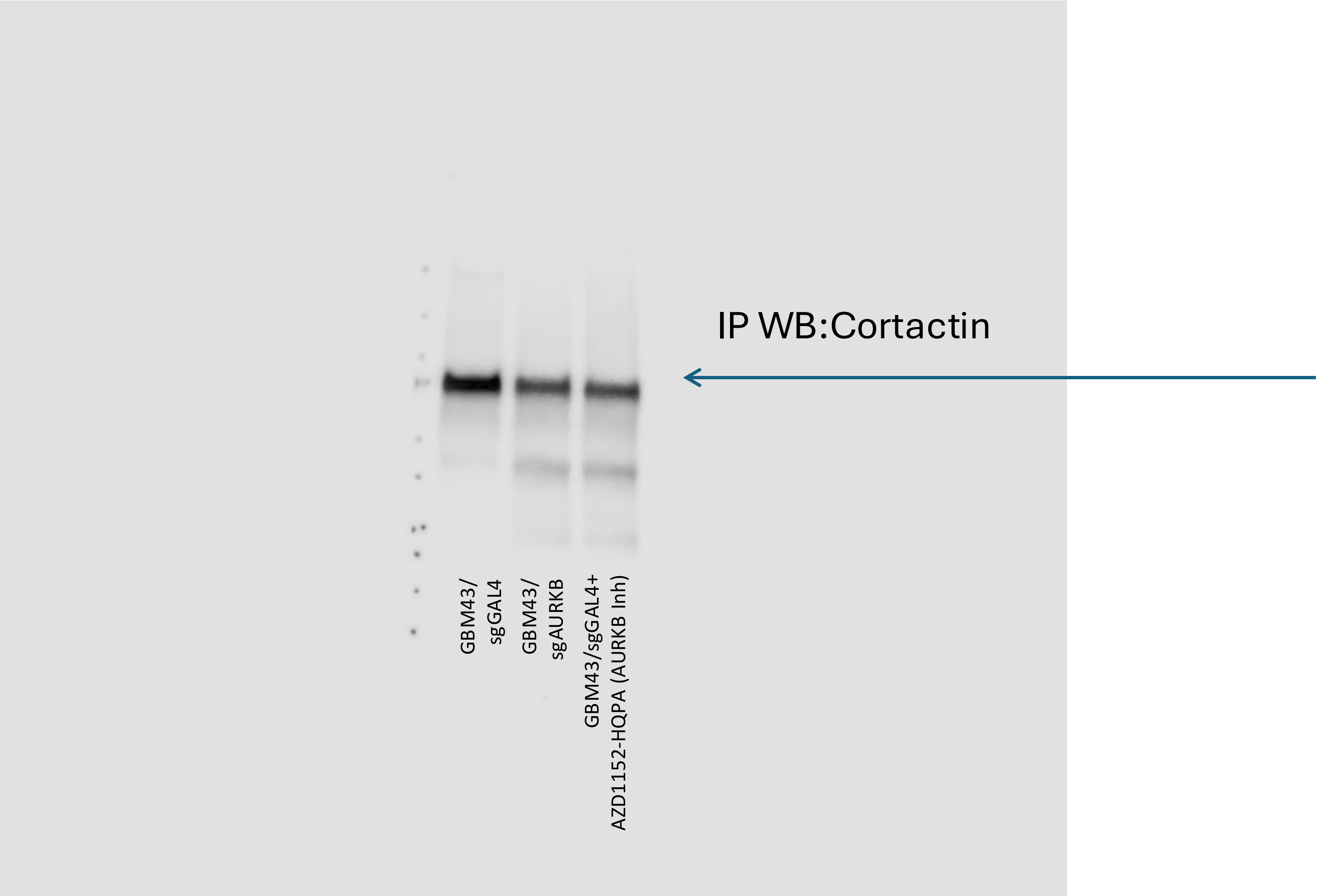

Supp Fig 4B right blots row 3

Input
WB: β-actin
Replicate #3

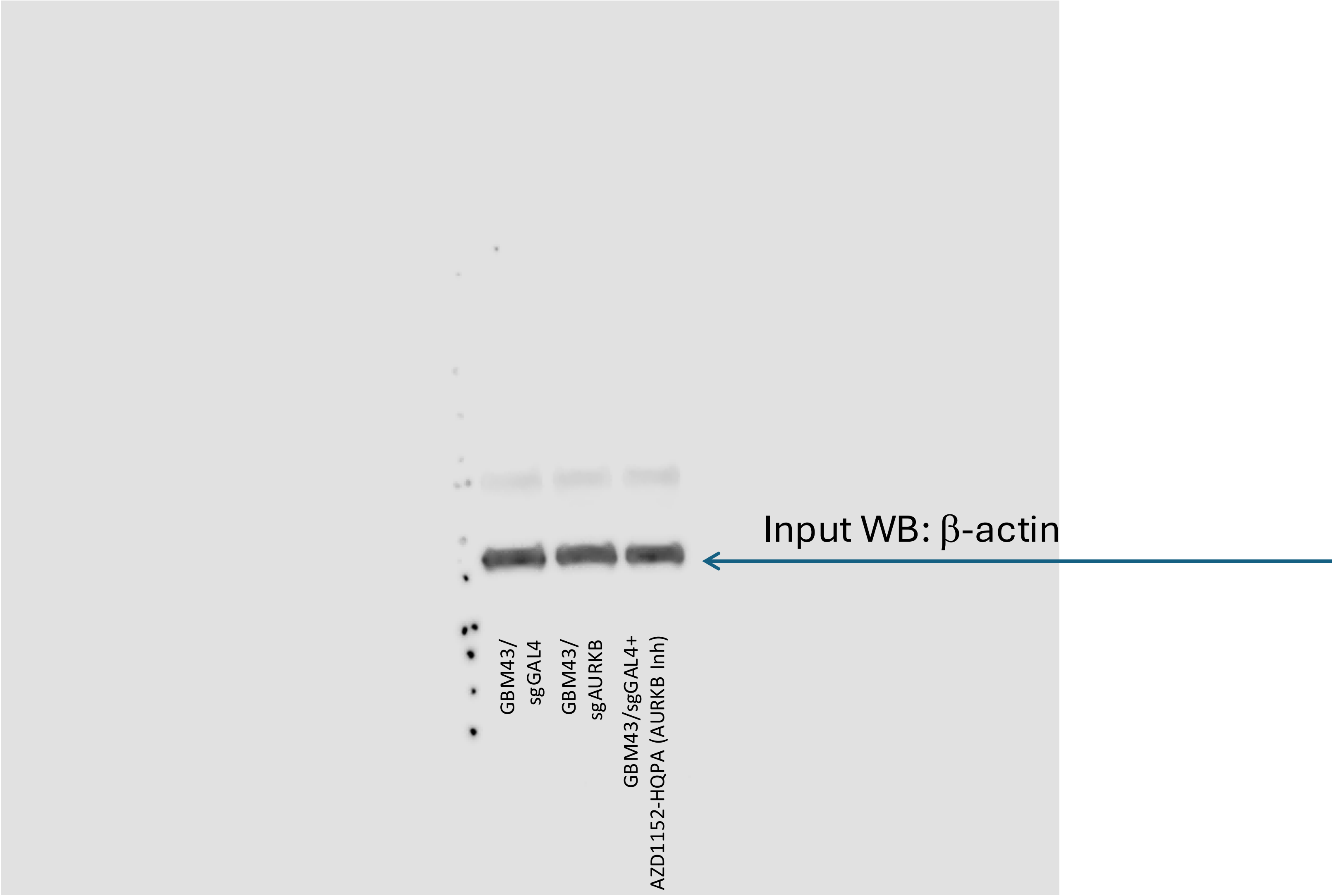

Supp Fig 4 C middle blots rows 1 and 2

IP: pTyr
WB: Cortactin and Heavy Chain
Replicate #2

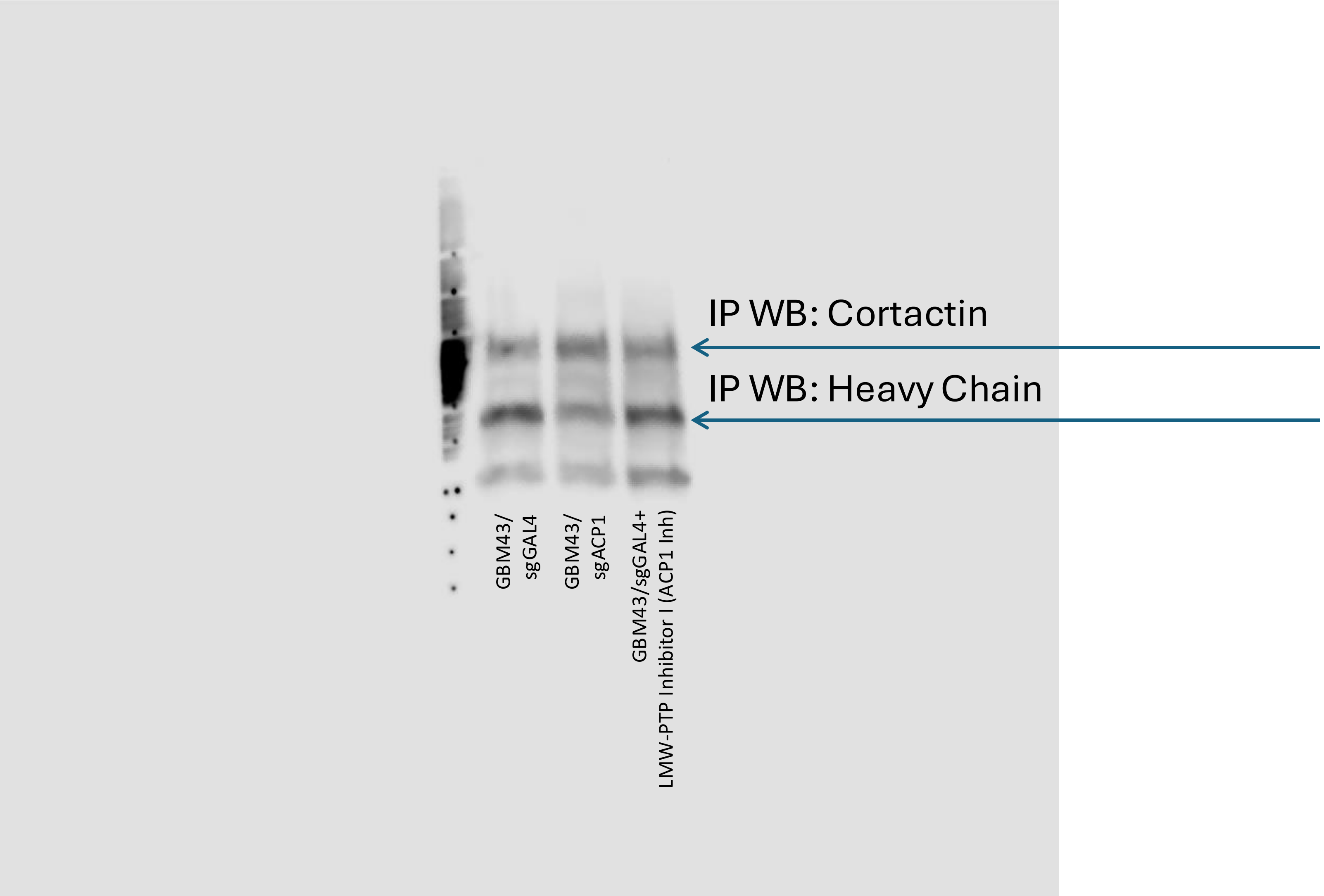

Supp Fig 4 C middle blots row 3

Input
WB: Cortactin
Replicate #2

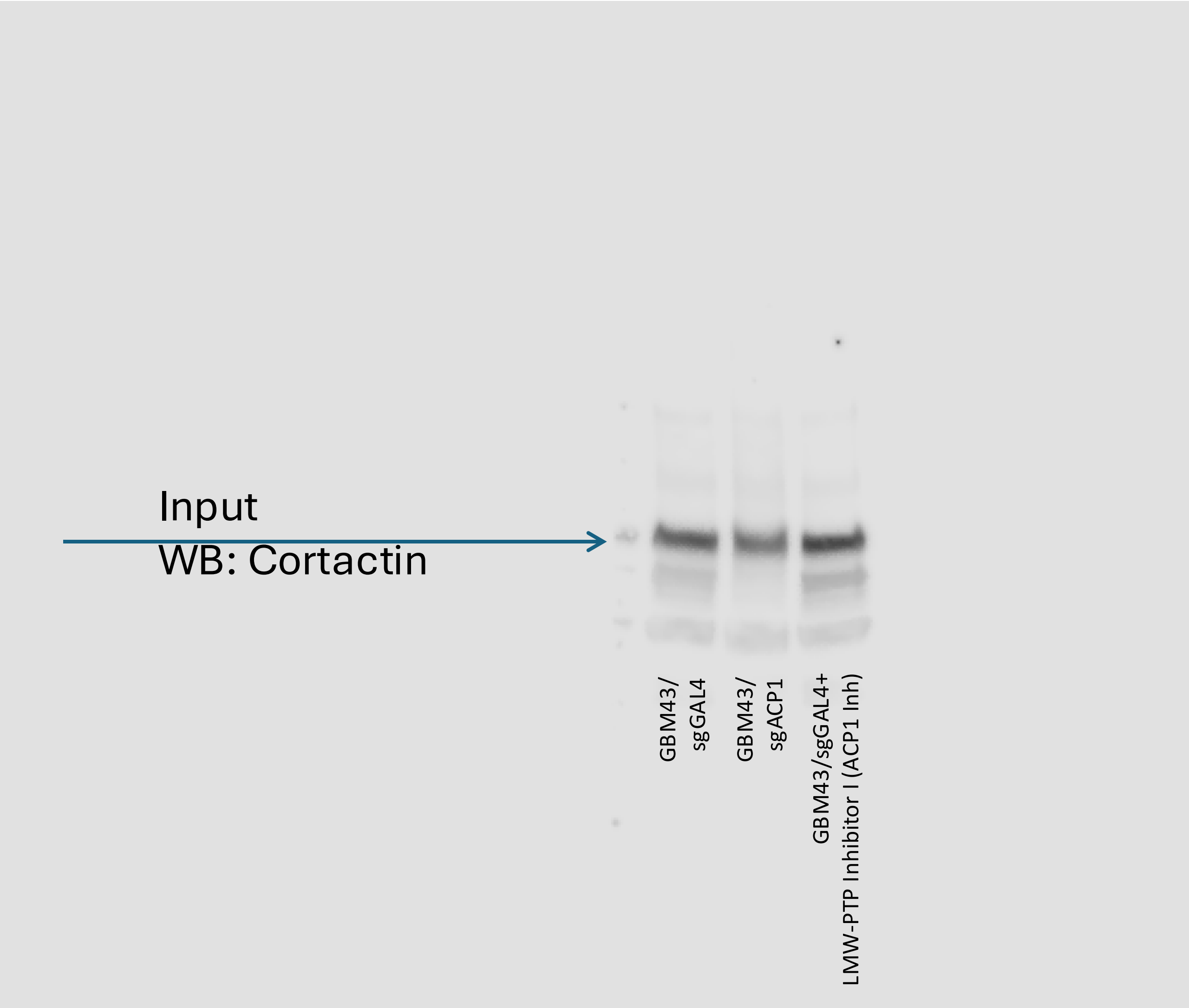

Supp Fig 4C middle blots row 4

Input
WB: β-actin
Replicate #2

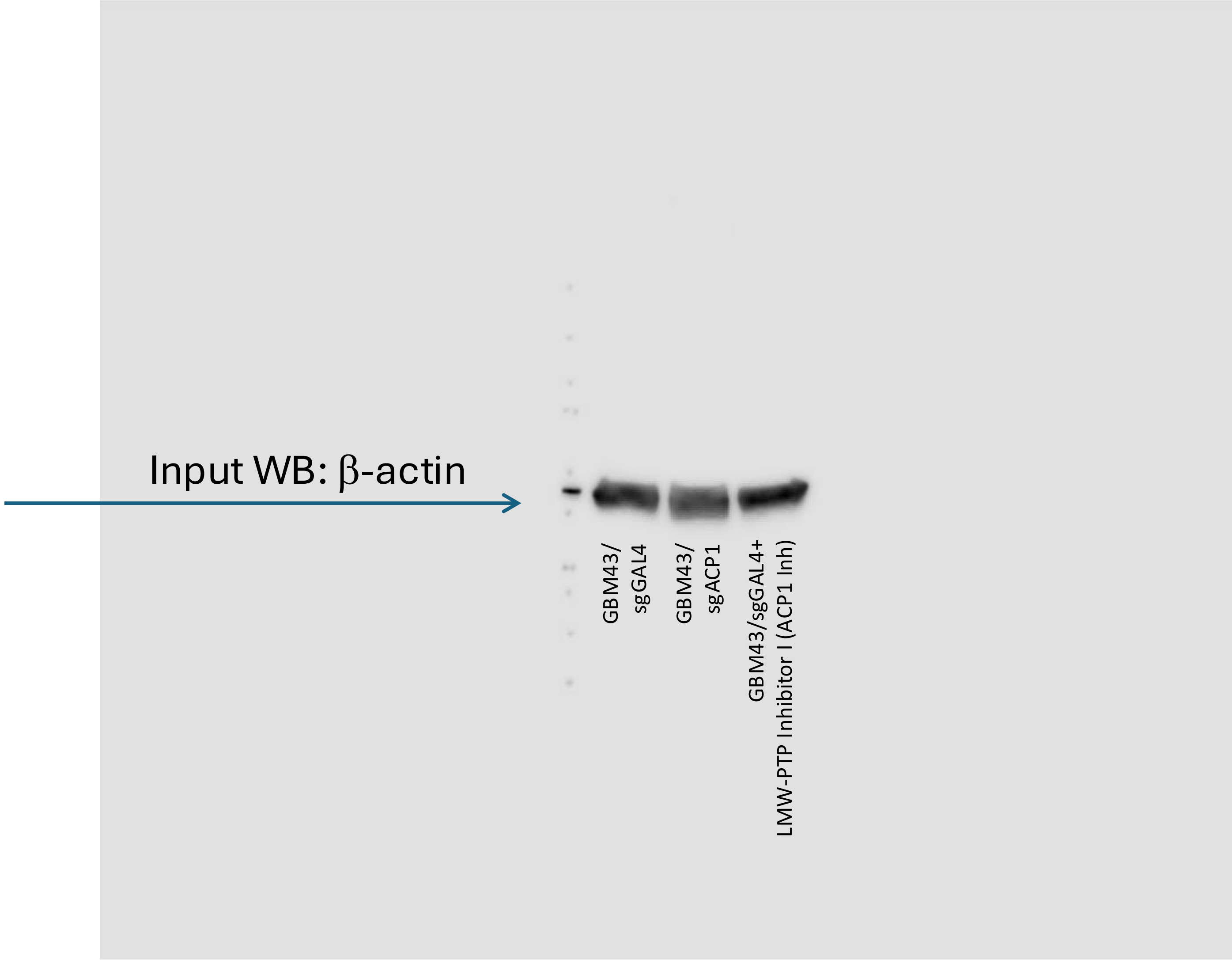

Supp Fig 4 C right figure rows 1 and 2

IP: pTyr
WB: Cortactin and Heavy Chain
Replicate #3

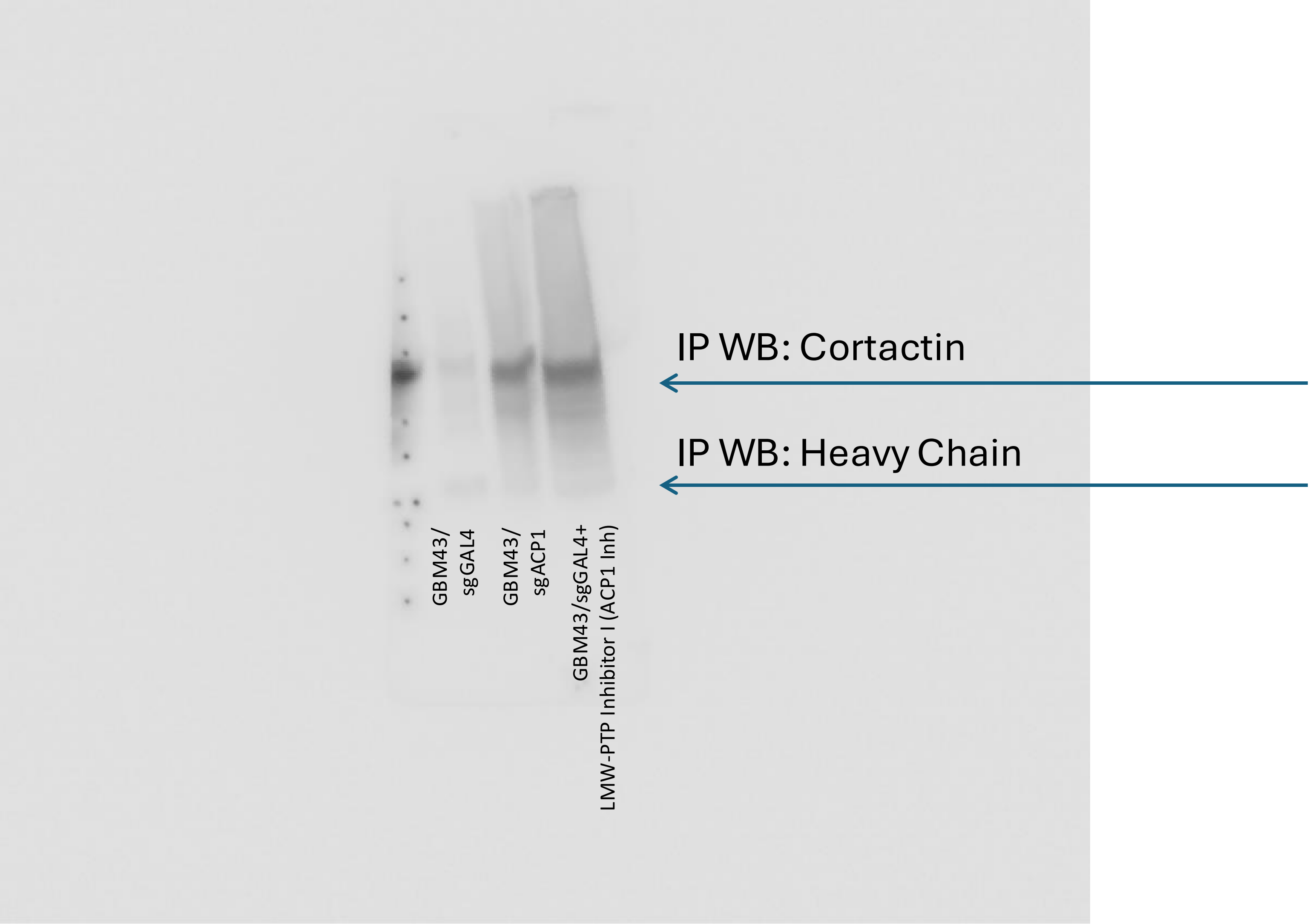

Supp Fig 4 C right figure row 3

Input
WB: Cortactin
Replicate #3

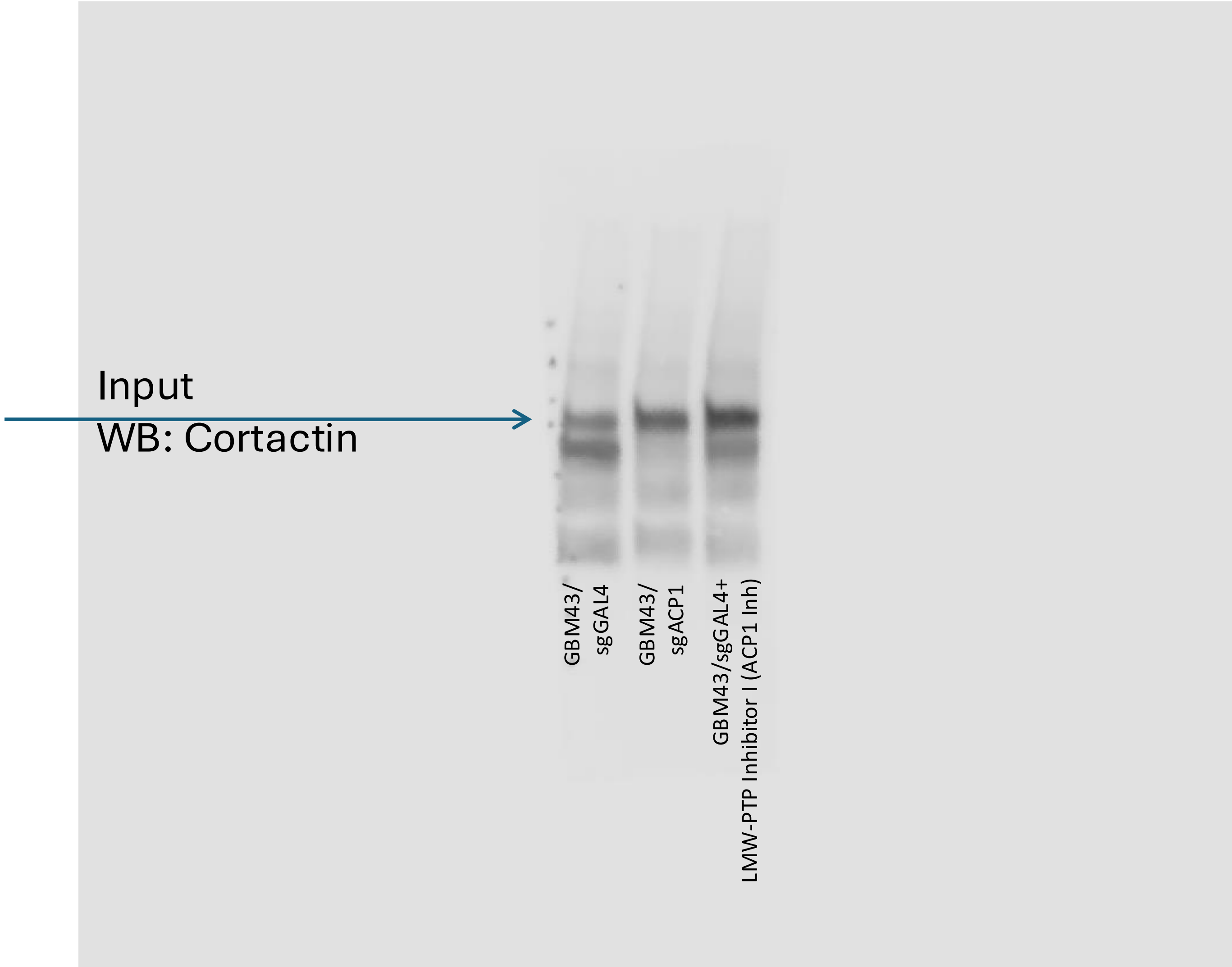

Supp Fig 4 C right figure row 4

Input
WB: β-actin
Replicate #3

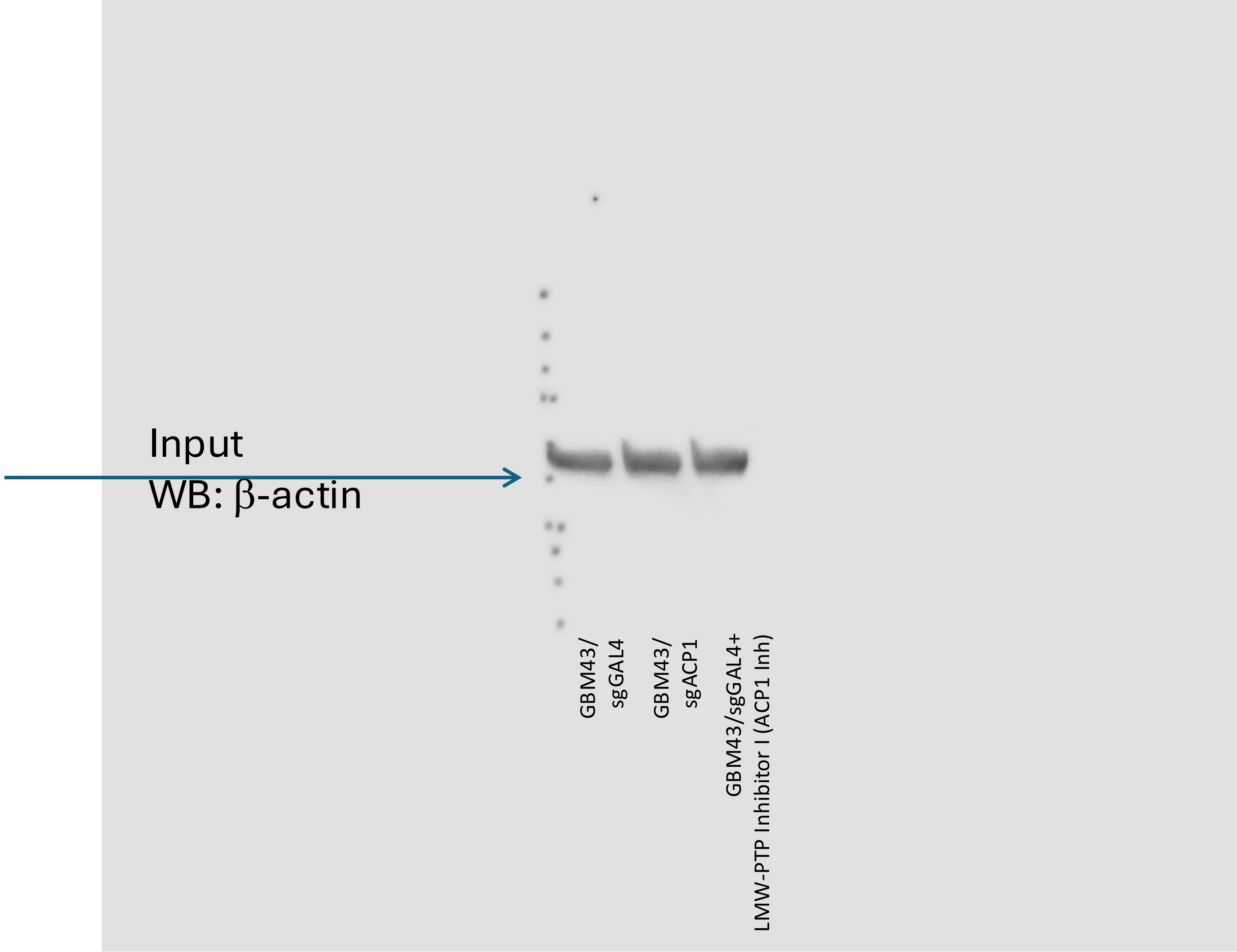

Supplement: Supplementary file 2 [file jciinsight-11-196484-s139.pdf]
